# Supplementary material for: Vascular and lymphatic dysregulation via non-EndoMT Col2a1 signaling in bisphosphonate-related osteonecrosis of the jaw
Source: Nat Commun. 2026 Sep 10;17:10715. doi: 10.1038/s41467-026-77692-w (PMC13658242; doi:10.1038/s41467-026-77692-w)
Supplement: Supplementary file 1 [file 41467_2026_77692_MOESM1_ESM.pdf]

Supplementary Information

Vascular and Lymphatic Dysregulation via Non-EndoMT *Col2a1* Signaling in Bisphosphonate-Related Osteonecrosis of the Jaw

Supplementary Figures

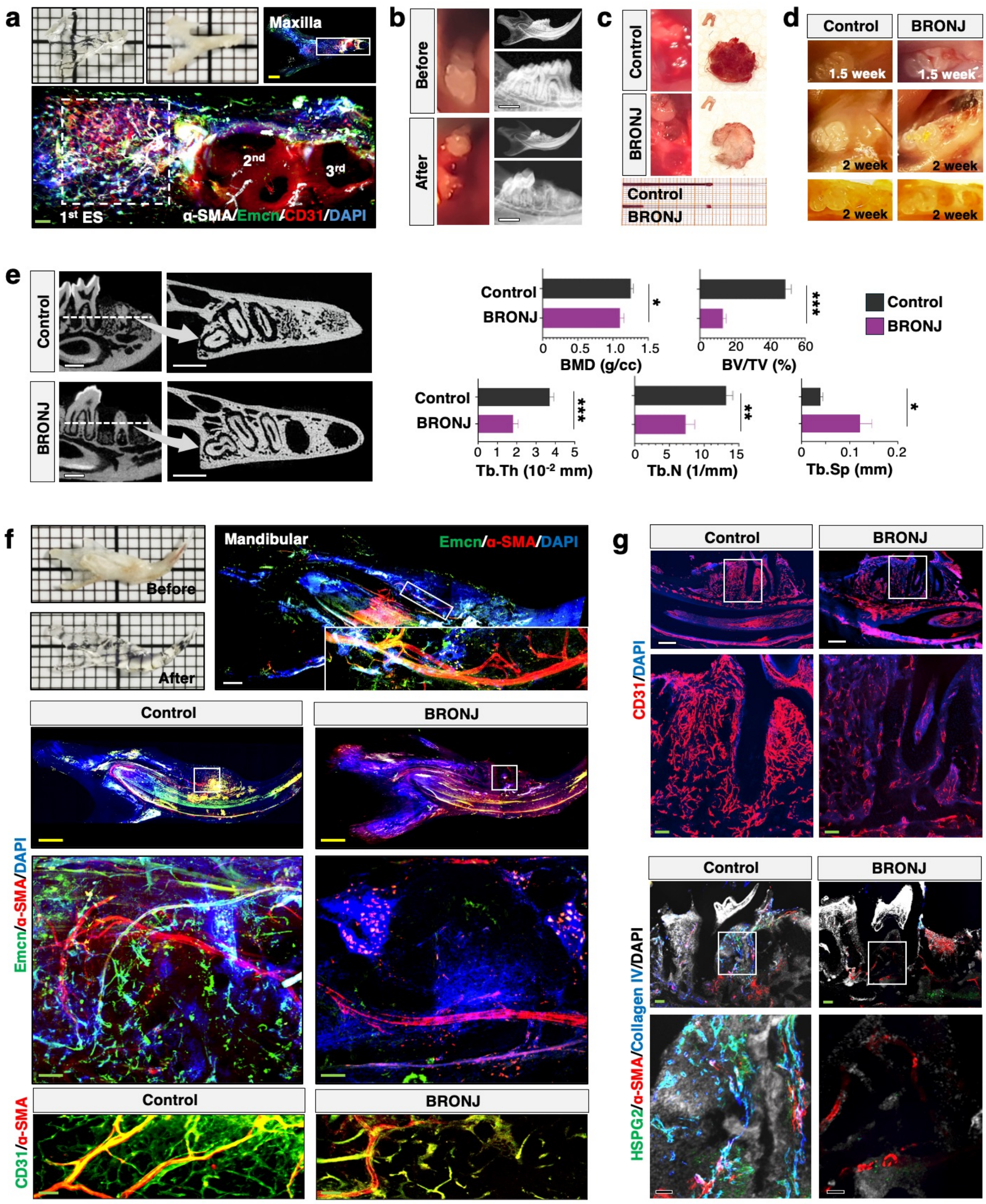

Supplementary Fig. 1: Establishment and characterization of the BRONJ model.
a Light-sheet imaging of cleared maxillae 2 weeks after first molar extraction, immunostained for α-SMA, Emcn, and CD31, with nuclear counterstaining by DAPI. **b** Intraoral and X-ray images of mandibles in control and BRONJ groups before and after extraction. **c** Intraoperative bleeding during extraction in control and BRONJ groups. **d** Intraoral images of extraction sockets (ES) in control and BRONJ groups. **e** MicroCT images and quantitative analysis of mandibular sockets at 2 weeks post-extraction *(n = 3)*. **f** Lightsheet imaging of cleared mandibles from control and BRONJ groups, immunostained for α-SMA, Emcn, and CD31 with DAPI counterstaining. **g** Representative 3D reconstructions of ES from control and BRONJ groups, showing CD31, HSPG2, α-SMA, Collagen IV, and DAPI. **Scale bars:** yellow, 1000 µm; white, 500 µm; green, 100 µm; black, 50 µm. *P < 0.05; ***P < 0.001. n represents biological replicates.

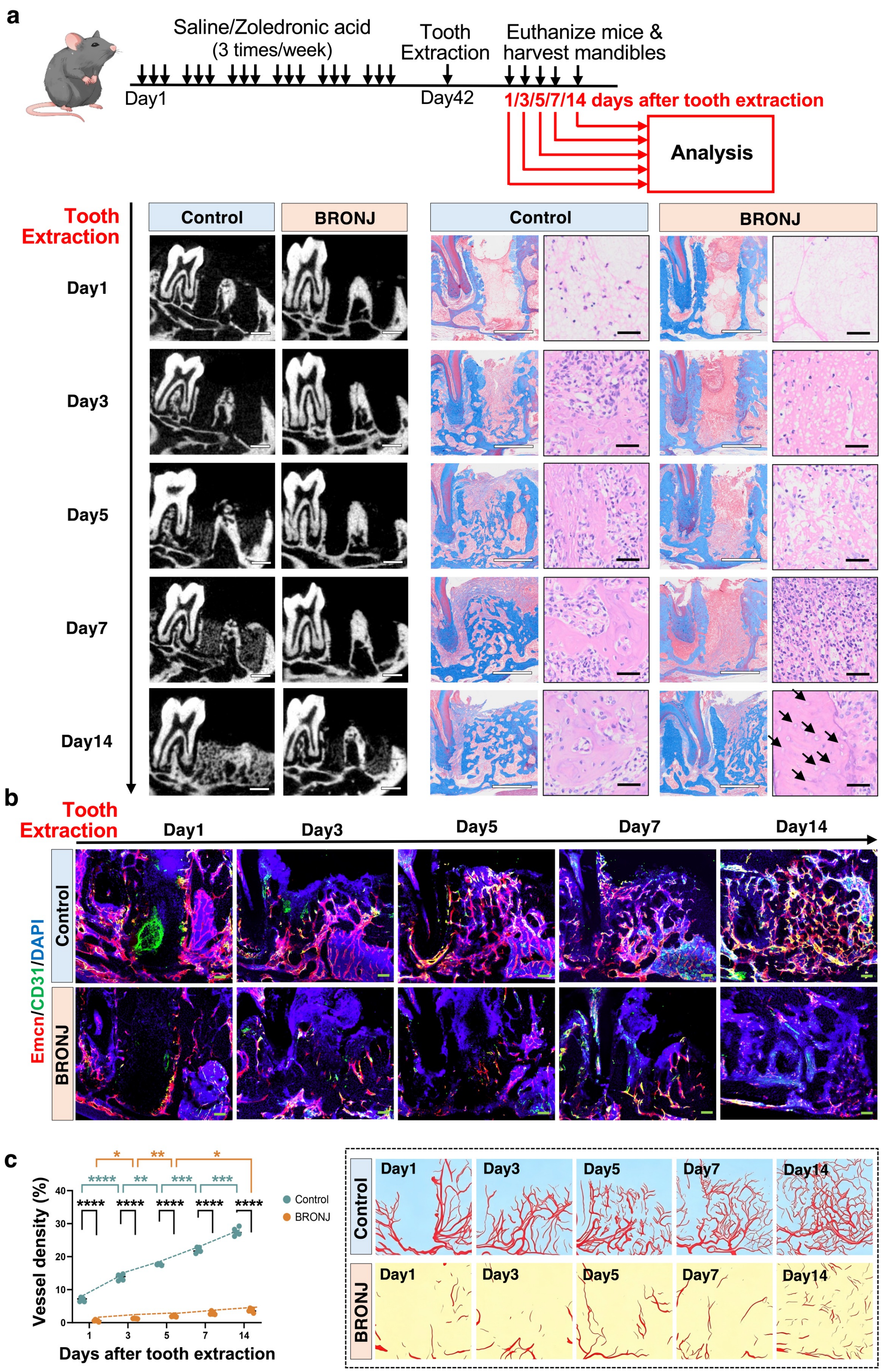

Supplementary Fig. 2: Time-course evaluation at 1, 3, 5, 7, and 14 days post-tooth extraction. **a** Representative micro-CT images of new bone formation, alongside histological assessments using Masson's trichrome and hematoxylin and eosin (H&E) staining in the control and BRONJ groups

at the indicated time points. **b** Representative immunofluorescence images of mandibular vasculature in the control and BRONJ groups at 1, 3, 5, 7, and 14 days post-extraction. Sections were immunostained for Emcn and CD31, with DAPI used for nuclear counterstaining. **c** Statistical analysis of vessel density changes over time in the Control and BRONJ groups (*n=5*), and schematic diagrams of vascular changes. **Scale bars:** white, 500 μm; green, 100 μm; black, 50 μm. *P < 0.05; **P < 0.01; ***P < 0.001; ****P < 0.0001. n represents biological replicates.

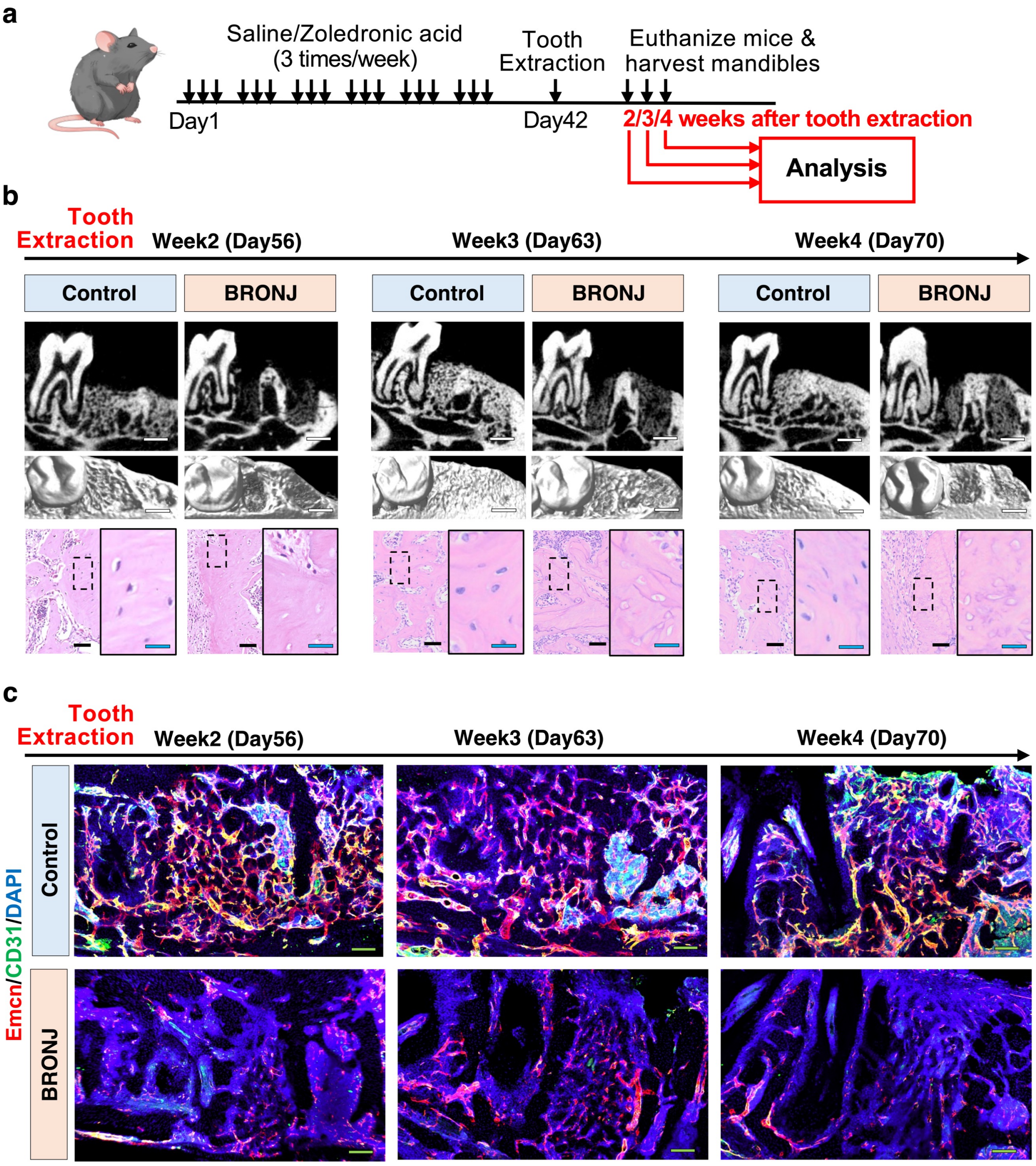

Supplementary Fig. 3: Time-course evaluation at 2, 3, and 4 weeks post-tooth extraction. **a** Schematic diagram illustrating the timeline for experimental model establishment. **b** Representative micro-CT images of new bone formation, alongside histological assessments using H&E staining in the control and BRONJ groups at the indicated time points. **c** Representative immunofluorescence images of mandibular vasculature in the control and BRONJ groups at 2, 3, and 4 weeks post-extraction. Sections were immunostained for Emcn and CD31, with DAPI used for nuclear counterstaining. **Scale bars:** white, 500 μm; green, 100 μm; black, 50 μm; blue, 20 μm.

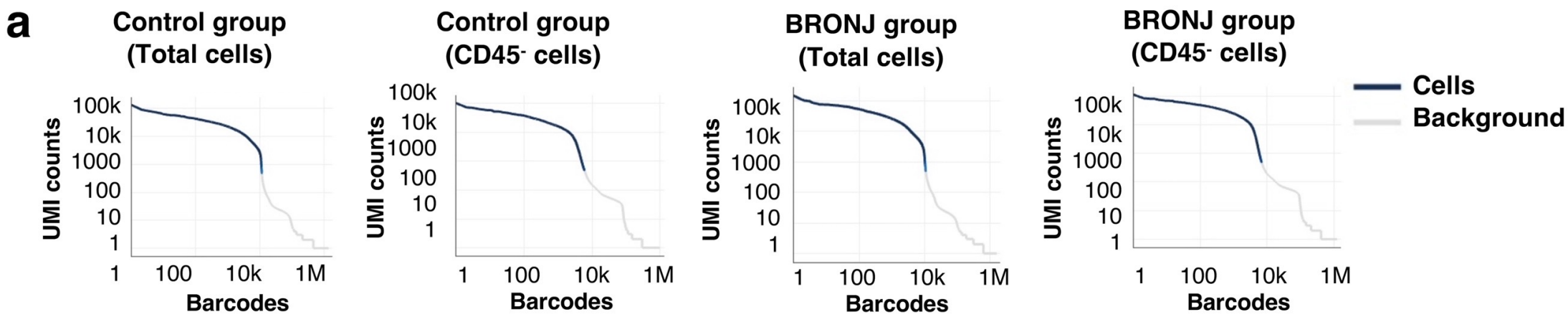

Samples	Control group (Total cells)	Control group ($CD45^-$ cells)	BRONJ group (Total cells)	BRONJ group ($CD45^-$ cells)
Estimated number of cells	11,459	5,838	10,424	6,629
Mean reads per cell	33,611	70,485	40,224	73,452
Median genes per cell	2,125	2,327	2,004	2,992
Number of reads	385,142,840	411,492,483	419,290,445	486,911,123
Valid barcodes	94.8%	96.5%	96.0%	96.0%
Valid UMIs	99.8%	99.8%	99.8%	99.8%
Sequencing saturation **(>30%)**	52.5%	78.7%	57.1%	72.2%
Q30 bases in barcode	91.9%	92.1%	91.1%	91.3%
Q30 bases in RNA read **(>70%)**	95.9%	96.0%	96.0%	96.1%
Q30 bases inUMI	91.4%	91.8%	90.6%	90.8%
Reads mapped confidently to transcriptome **(>30%)**	79.2%	78.1%	80.7%	80.2%
Fraction reads in cells **(>60%)**	96.6%	92.3%	96.0%	93.2%

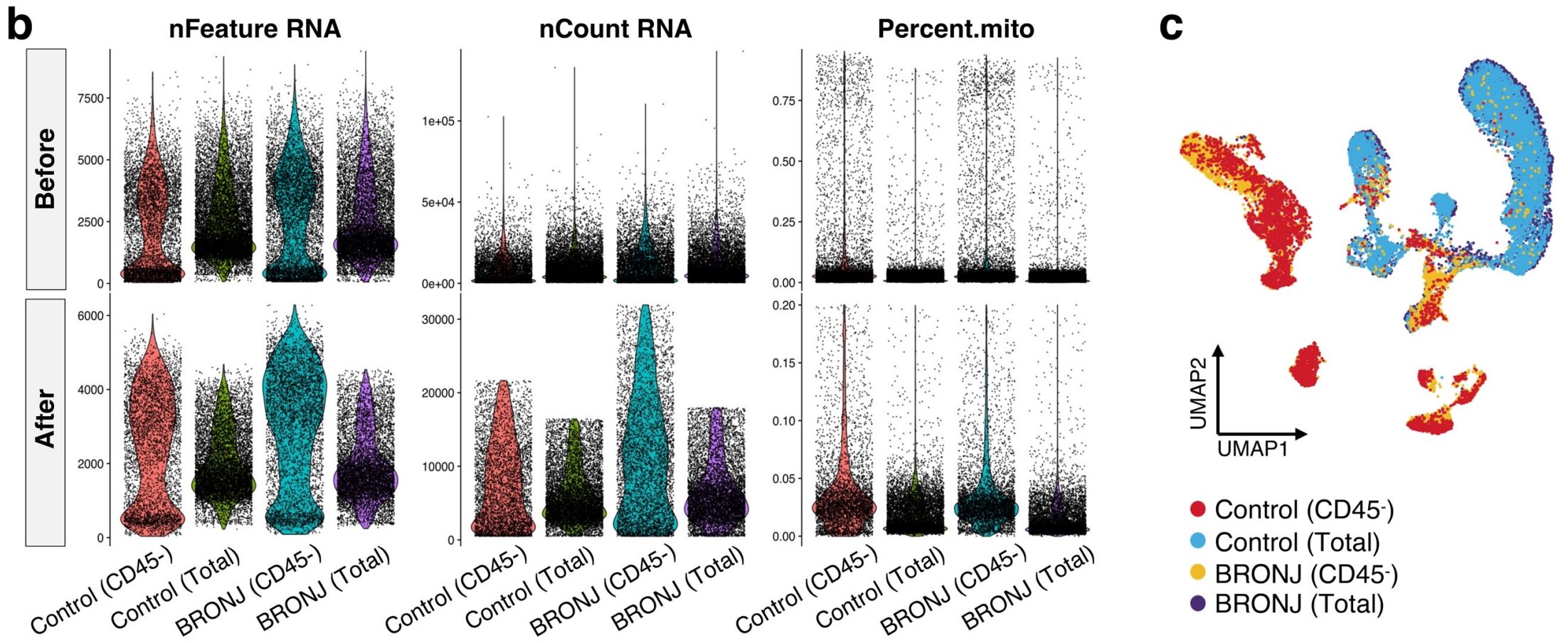

Supplementary Fig. 4: Quality control of single-cell RNA-seq samples.

a Cell Ranger QC metrics for four samples: control pre-sort total cells, control post-sort $CD45^-$ cells, BRONJ pre-sort total cells, and BRONJ post-sort $CD45^-$ cells. **b** Removal of doublets and low-quality cells. **c** UMAP projections of the four samples after quality control.

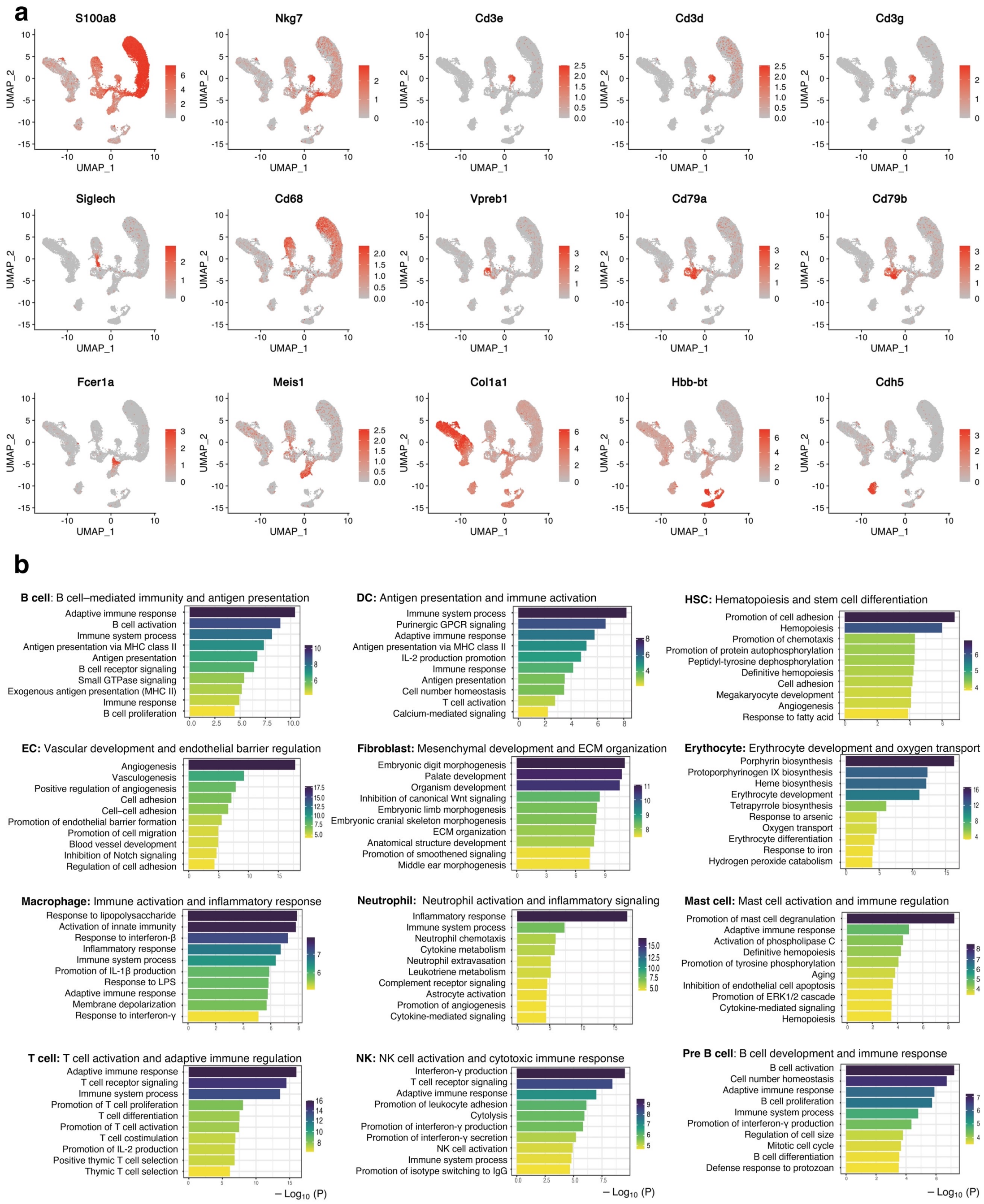

Supplementary Fig. 5: Cell type identification and functional annotation.

a UMAP projection of marker gene expression used for cell type annotation. **b** GO enrichment of the top 100 marker genes per cluster showing representative biological processes.

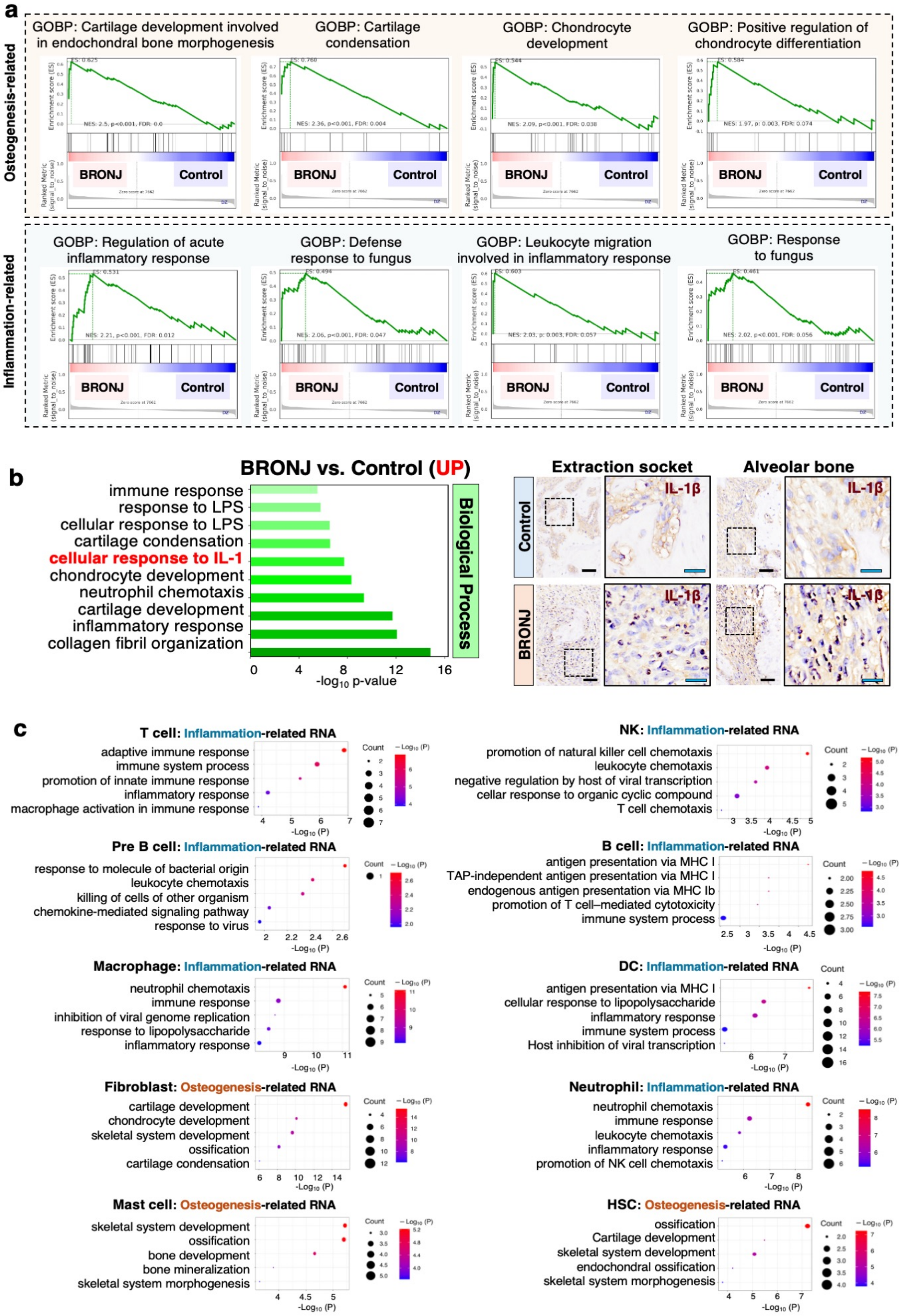

Supplementary Fig. 6: Functional enrichment of upregulated genes in BRONJ.

a Gene set enrichment analysis (GSEA) of BRONJ-upregulated genes, highlighting osteogenesis- and inflammation-related pathways. **b** GO functional enrichment analysis of the upregulated genes in the BRONJ group compared to the control. **c** Representative immunohistochemical staining images of IL-1β in the extraction socket and alveolar bone. **Scale bars:** black, 50 μm; blue, 20 μm.GO enrichment analysis of upregulated genes across 10 cell types.

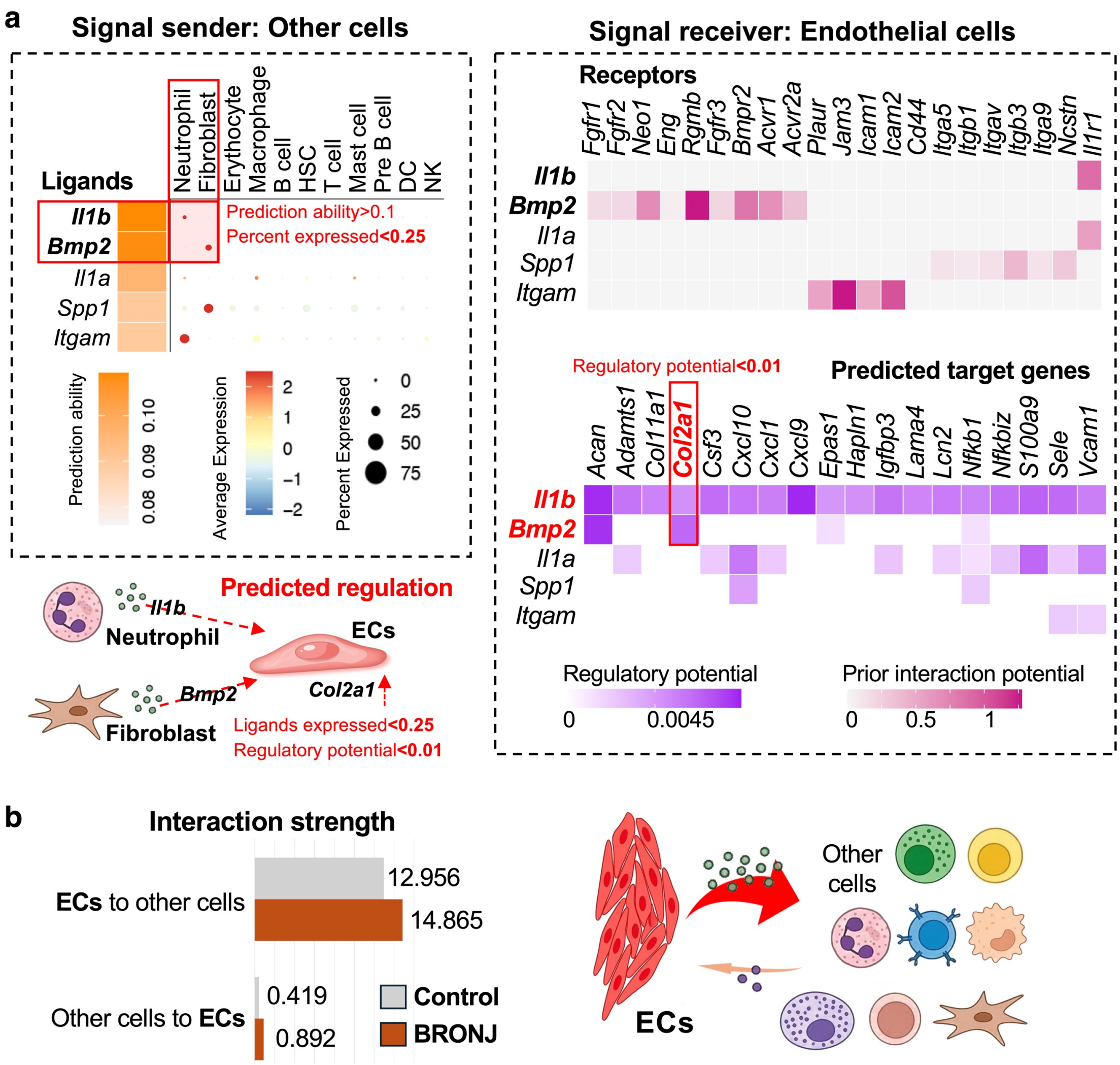

Supplementary Fig. 7: Signaling dynamics and inflammatory assessment in the mandible. **a** NicheNet analysis predicting key ligand-receptor interactions, designating other cell populations as signal senders and endothelial cells (ECs) as receivers, with EC differentially expressed genes serving as the predicted target genes. **b** Comparison of intercellular interaction strengths (ECs to other cells, and other cells to ECs), accompanied by a schematic illustration detailing the differences in signaling intensities.

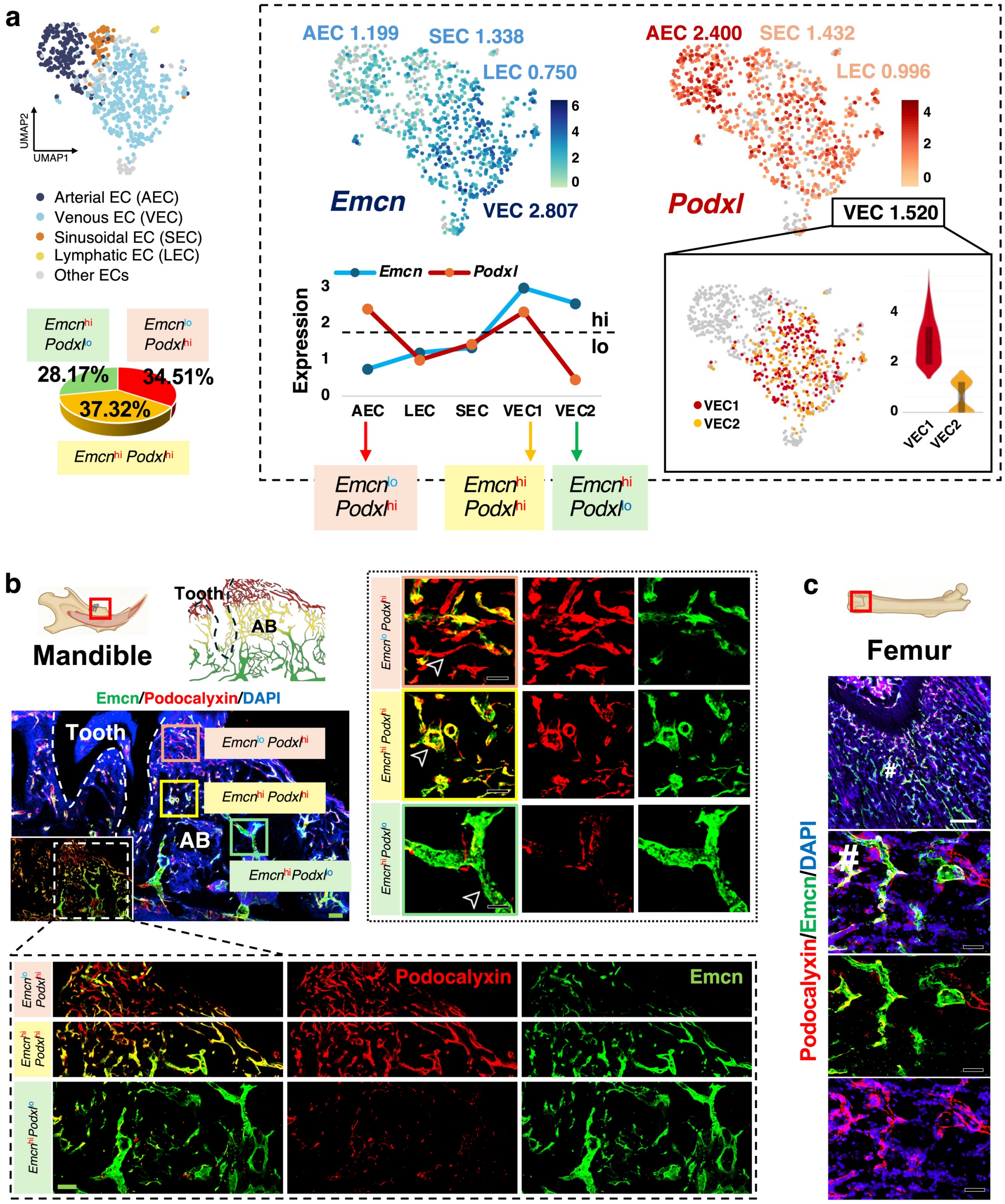

Supplementary Fig. 8: Characterization and spatial distribution of endothelial cell subtypes. **a** scRNA-seq analysis detailing the expression profiles, relative abundance, and cluster distribution of Emcn and Podxl across endothelial cell subpopulations. **b** Representative immunofluorescence images illustrating the spatial localization and abundance of Emcn^{hi} and Podxl^{hi} vascular networks within the mandible. The right and bottom panels show high- and low-magnification views, respectively. **c** Immunofluorescence staining of Emcn and Podxl expression in the femur, with nuclei counterstained by DAPI. **Scale bars:** white, 500 µm; green, 100 µm; black, 50 µm.

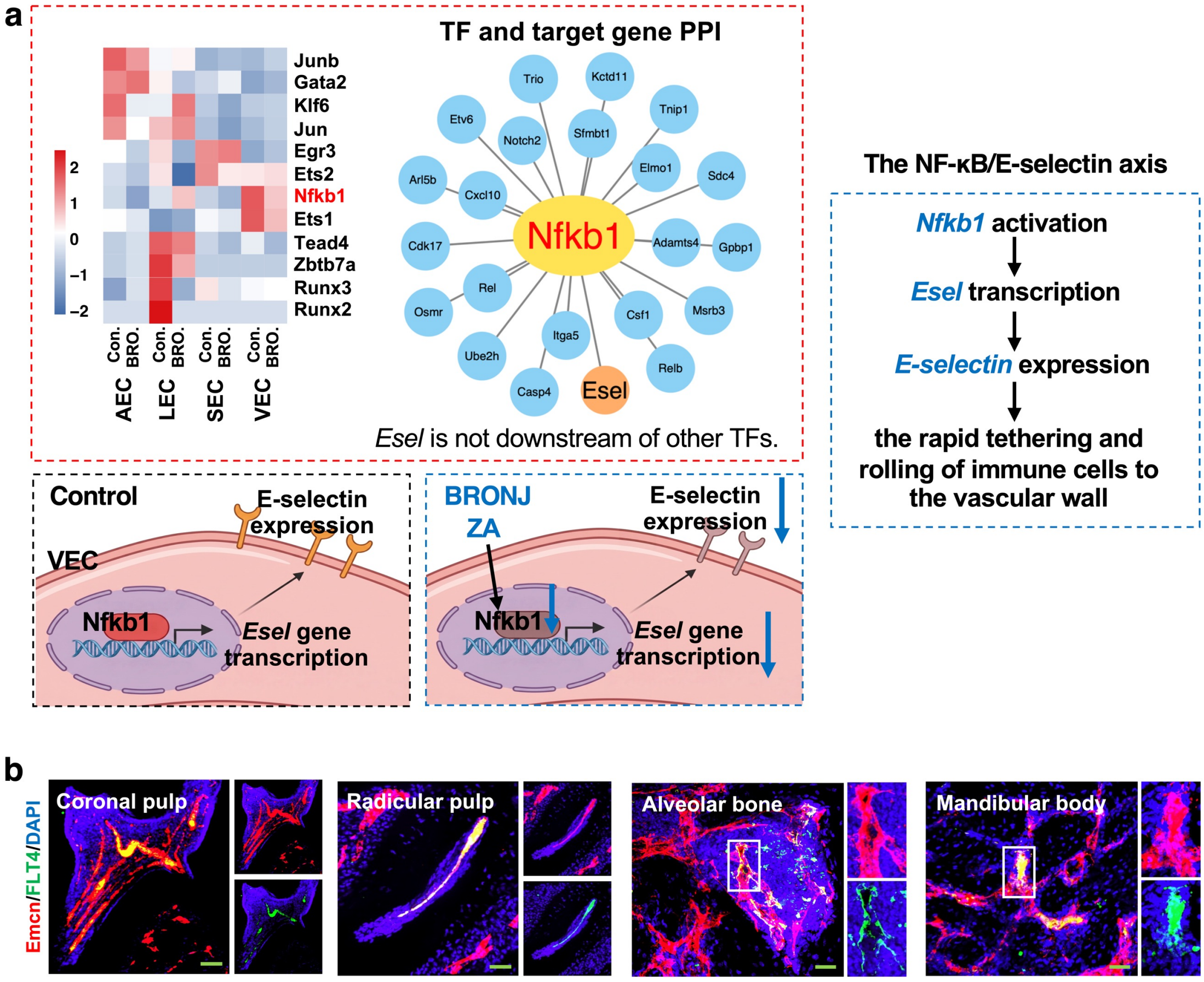

Supplementary Fig. 9: Phenotypic and transcriptional characterization of endothelial cells.
a SCENIC analysis of endothelial cell subtypes revealing differential transcription factor (TF) activities and the protein-protein interaction (PPI) network of their target genes. The schematic diagram illustrates the predicted regulatory mechanism underlying *Esel* downregulation. **b** Lymphatic vessels in mandible immunostained for FLT4 and Emcn, with nuclear counterstaining by DAPI. **Scale bars:** green, 100 μm; blue, 20 μm.

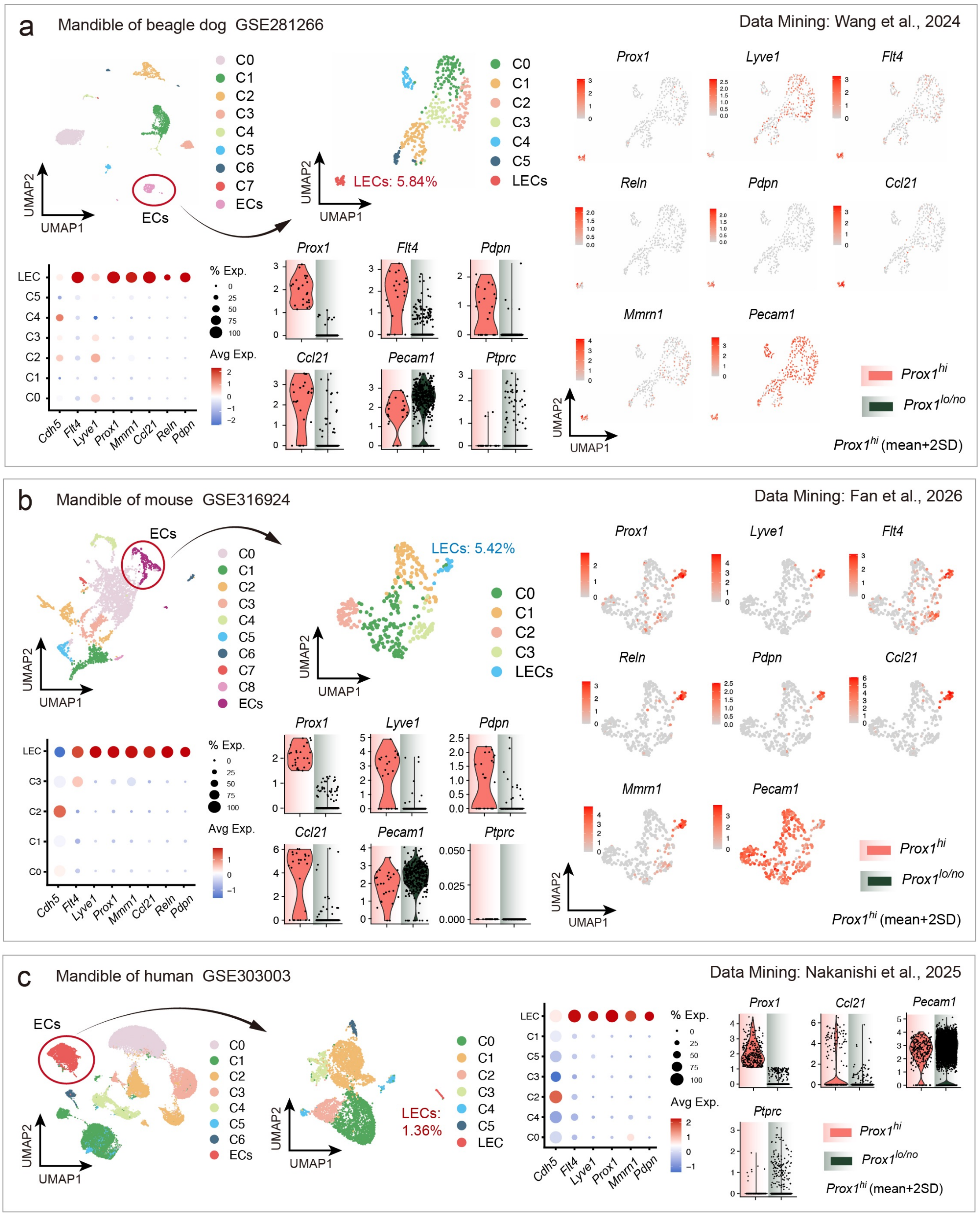

Supplementary Fig. 10: Transcriptional profiles of lymphatic endothelial cells across multiple species from public datasets.

a Analysis of lymphatic endothelial cells in the mandible of beagle dogs derived from the GSE281266 dataset. **b** Analysis of lymphatic endothelial cells in the mouse mandible derived from the GSE316924 dataset. **c** Comparative analysis of mandibular lymphatic endothelial cells between the control and MRONJ groups using the human dataset GSE303003.

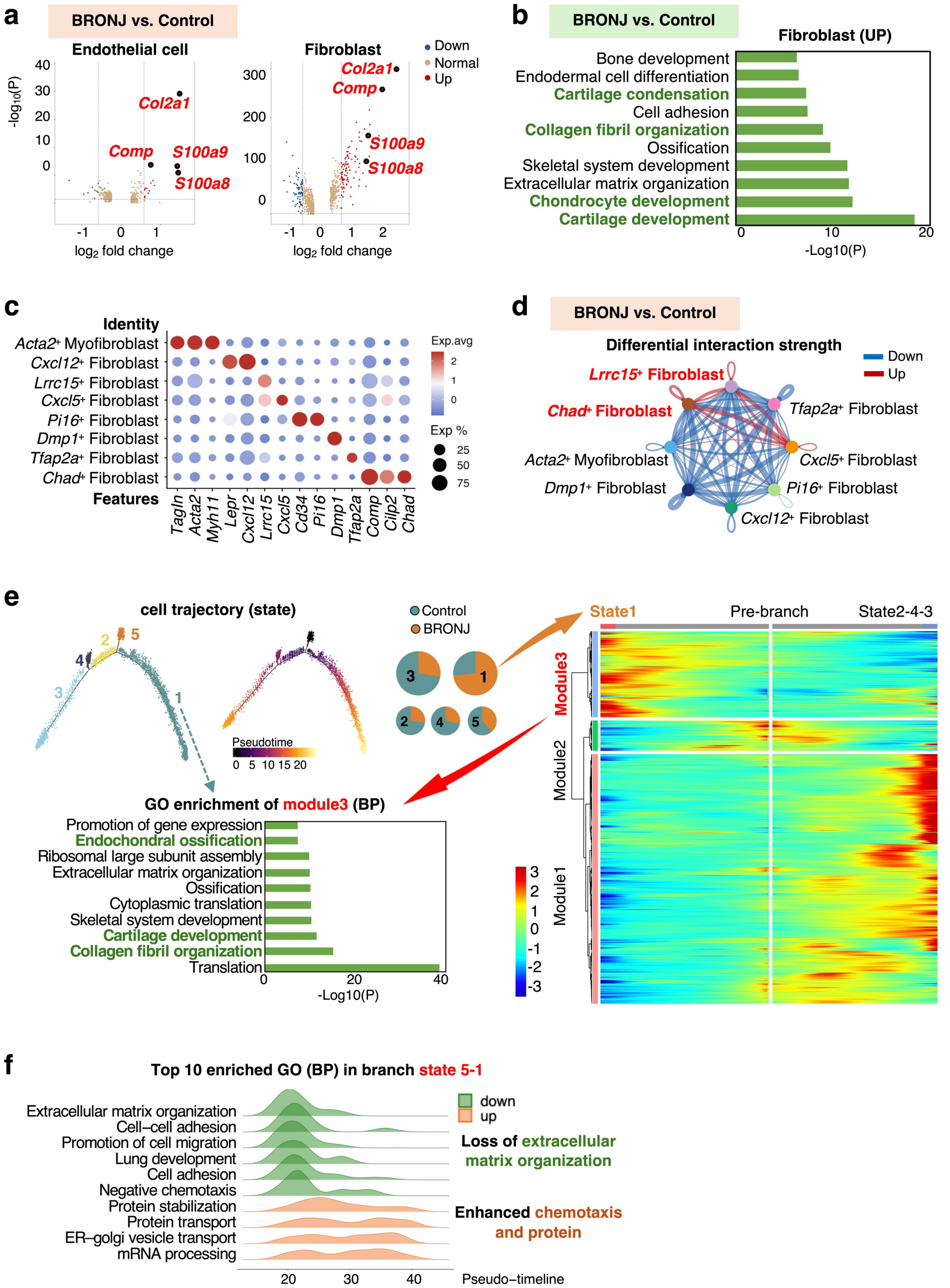

Supplementary Fig. 11: Fibroblast subtypes and differentiation trajectory analysis.

a Volcano plots of differentially expressed genes in endothelial cells and fibroblasts. **b** GO enrichment of BRONJ-upregulated genes in fibroblasts. **c** Bubble plot of marker gene expression across eight fibroblast subtypes. **d** CellChat analysis of interaction changes among fibroblast subtypes. **e** Pseudotime analysis of fibroblasts with Monocle2, showing modules by state and highlighting GO enrichment of Module 3. **f** Top ten enriched GO biological processes for branch state 5-1 with functional annotations.

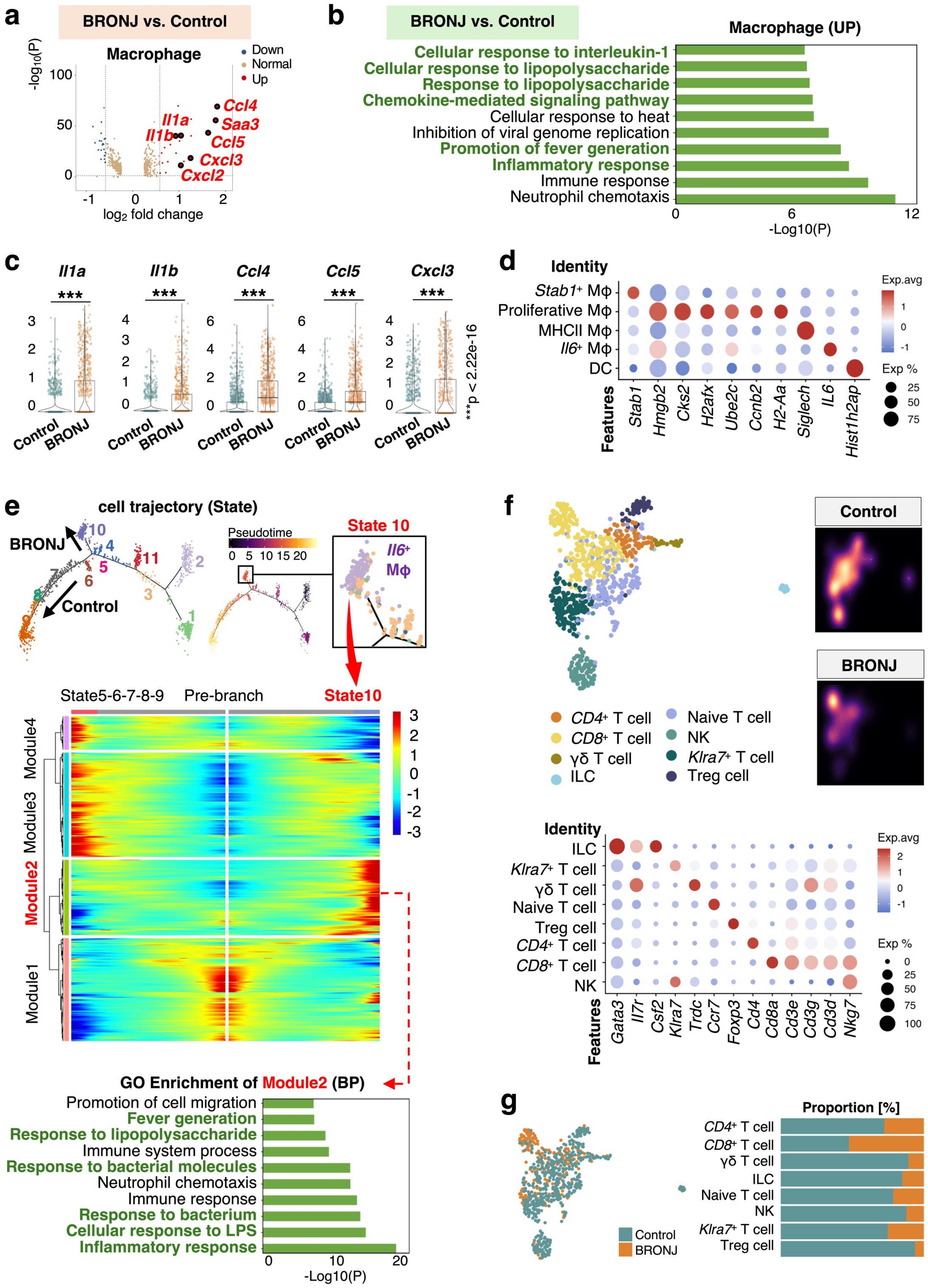

Supplementary Fig. 12: macrophage-DC and NK-T cell heterogeneity.

a Volcano plot of differentially expressed genes in macrophages. **b** GO enrichment of BRONJ-upregulated macrophage genes. **c** Differential expression of inflammatory genes. **d** Bubble plot of marker expression defining five macrophage subtypes. **e** Monocle2 pseudotime of fibroblasts showing state assignments and GO enrichment for state 10. **f** NK-T cell subtype taxonomy with marker expression. **g** Proportions of NK-T cell subtypes in control and BRONJ.

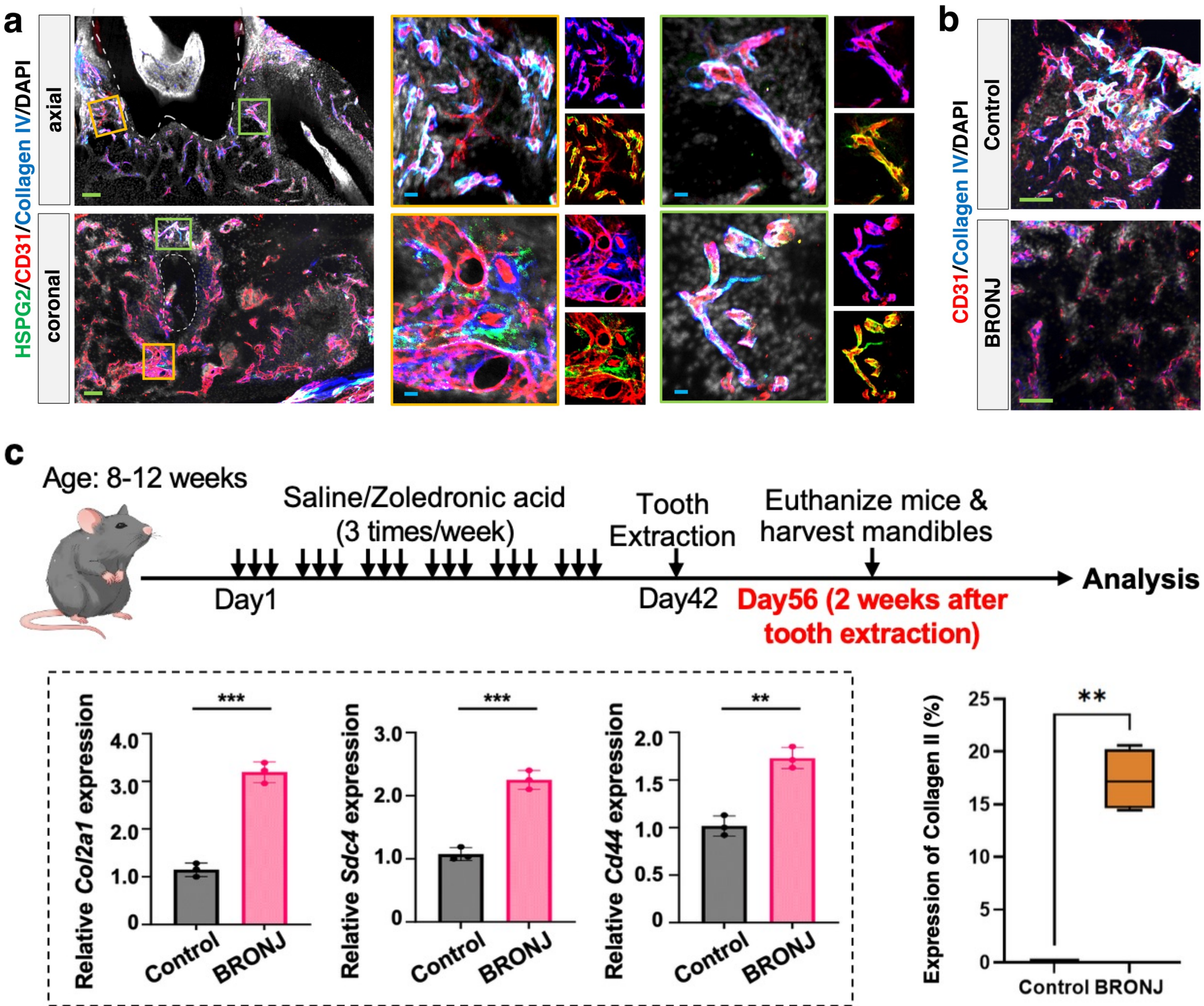

Supplementary Fig. 13: BRONJ rewires endothelial-collagen networks.

a Axial and coronal mandibular sections immunostained for HSPG2, CD31, and Collagen IV, with nuclear counterstaining by DAPI. **b** Comparative immunostaining images for CD31 and Collagen IV with DAPI counterstaining. **c** b RT-qPCR evaluation of *Col2a1* and its associated receptors *Sdc4* and *Cd44* in BRONJ (*n=3*), alongside statistical analysis of Collagen II immunofluorescence staining (*n=4*). **Scale bars:** green, 100 µm; blue, 20 µm; red, 5 µm. $^{**}P < 0.01$; $^{***}P < 0.001$.

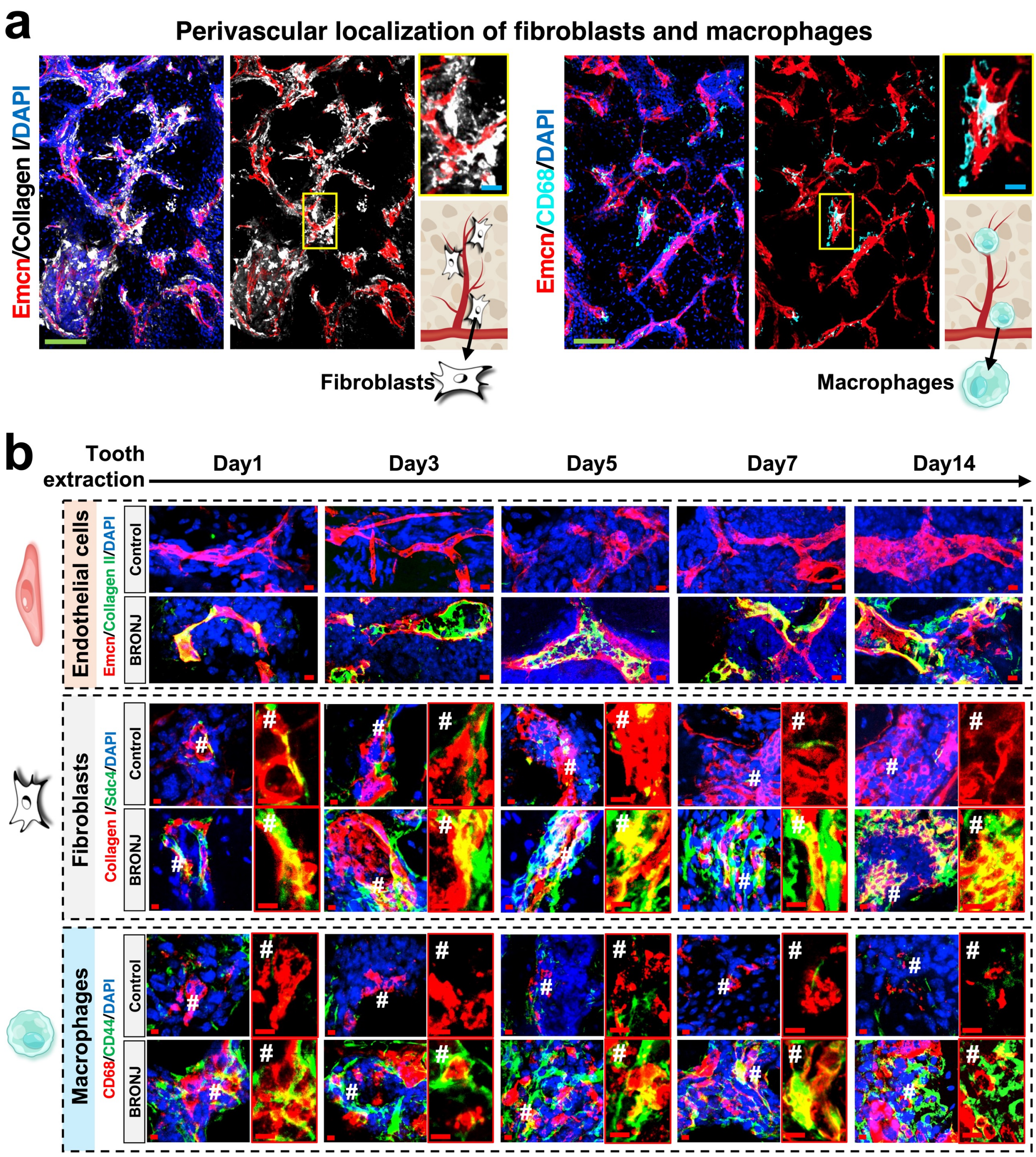

Supplementary Fig. 14: Spatial and temporal characterization of *Col2a1-Sdc4/Cd44* signaling. **a** Perivascular distribution of Collagen I^{+} fibroblasts and CD68^{+} macrophages around Emcn^{+} blood vessels (nuclei counterstained with DAPI). **b** Time-course immunolabeling of endothelial Col2a1 ligand and its receptors Sdc4 (fibroblasts) and Cd44 (macrophages) at days 1, 3, 5, 7, and 14 after tooth extraction. **Scale bars**: blue, 20 µm; red, 5 µm.

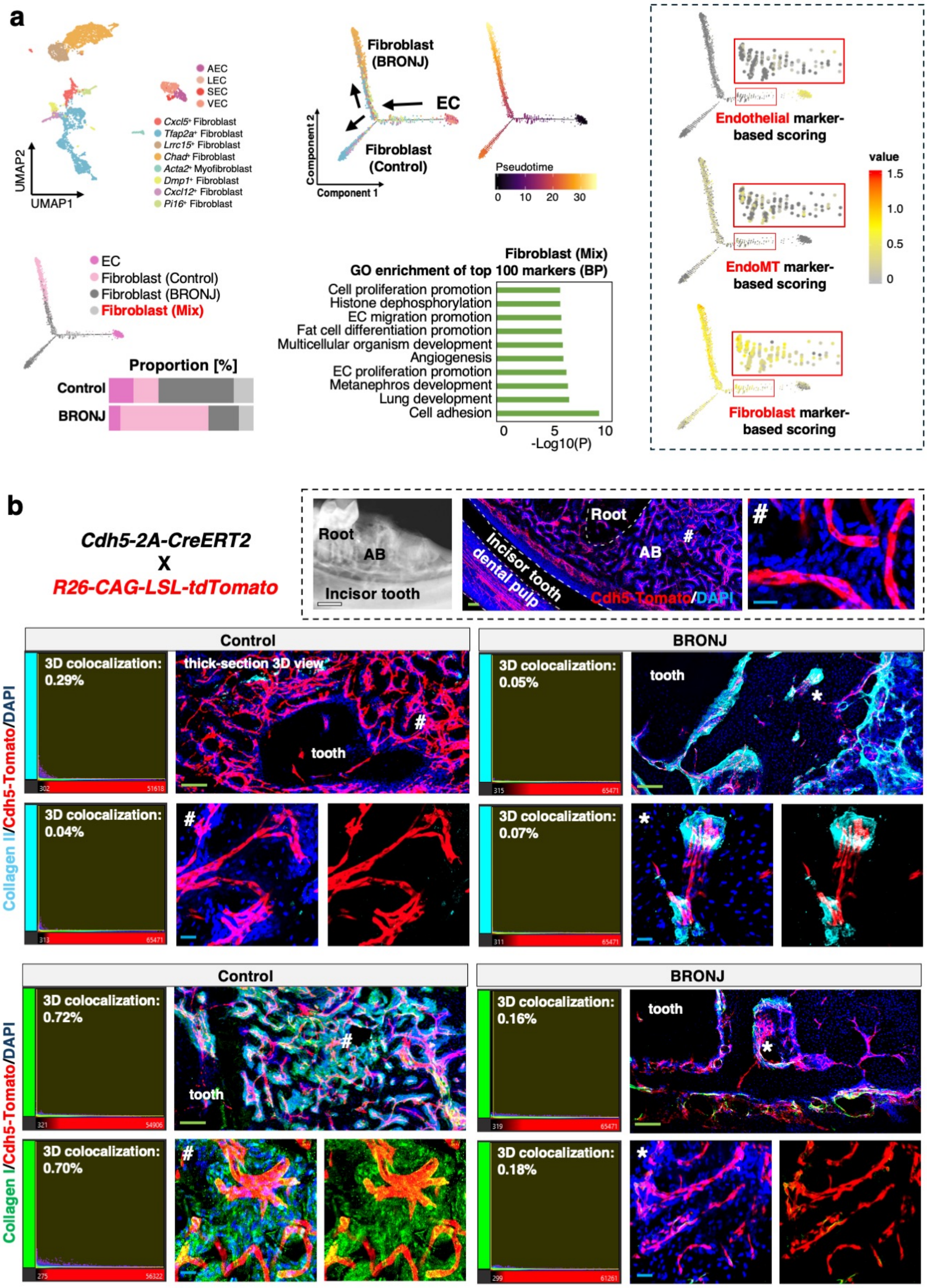

Supplementary Fig. 15: Transcriptional trajectory and lineage tracing validation of EndoMT. **a** Monocle 2 pseudotime trajectory analysis of the EC-fibroblast populations, displaying gene-set scores for endothelial, EndoMT, and fibroblast marker panels. The panel also illustrates the reclustering of these populations into four distinct subtypes, accompanied by GO enrichment analysis of fibroblast-associated (mixed) markers. **b** Lineage tracing in *Cdh5-2A-CreERT2; R26-CAG-LSL-tdTomato* mice showing semi-quantitative 3D colocalization in healthy and BRONJ mandibles, immunostained for Collagen II/I with nuclear counterstaining by DAPI. **Scale bars:** white, 500 μm; green, 100 μm; and blue, 20 μm.

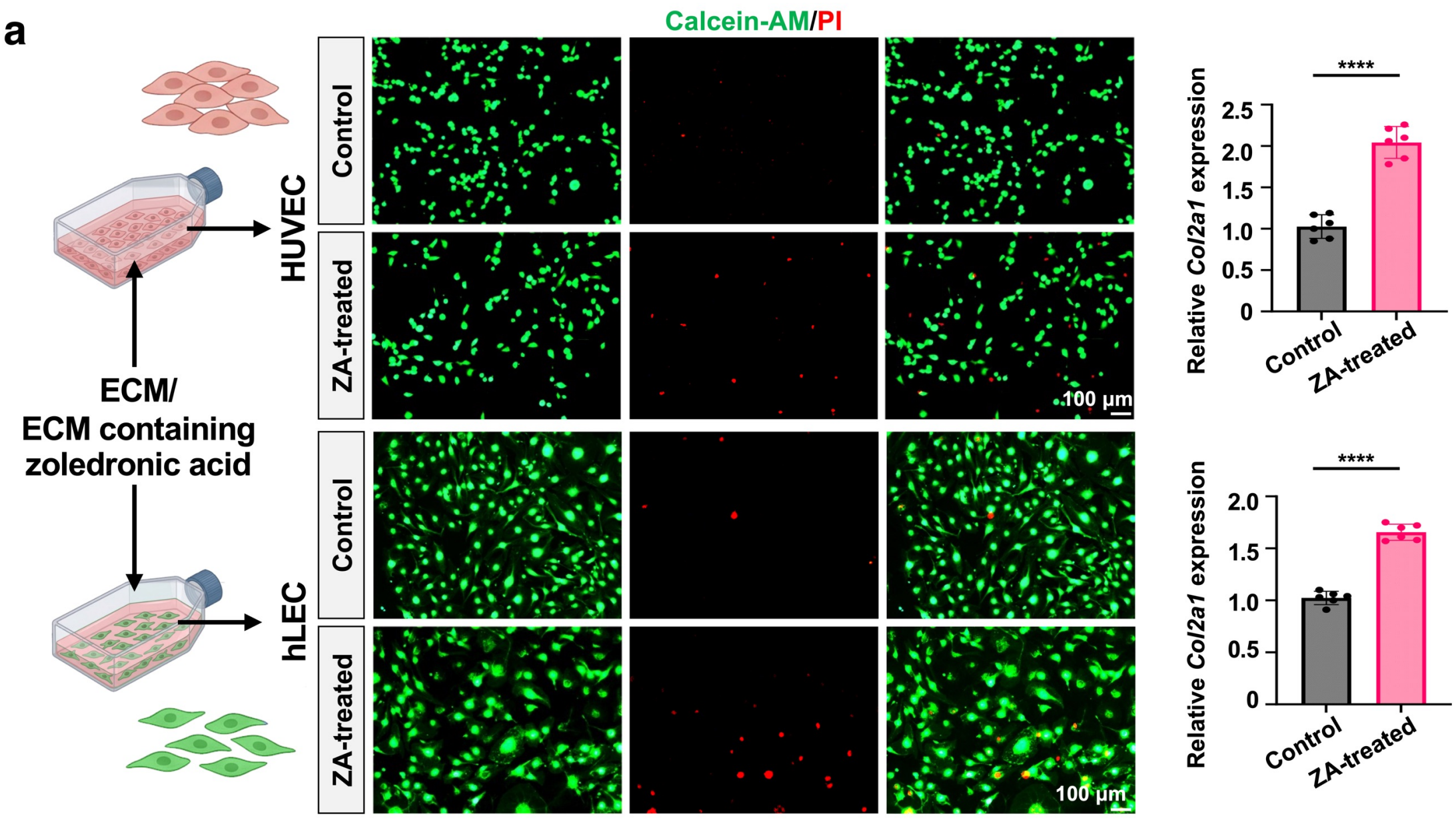

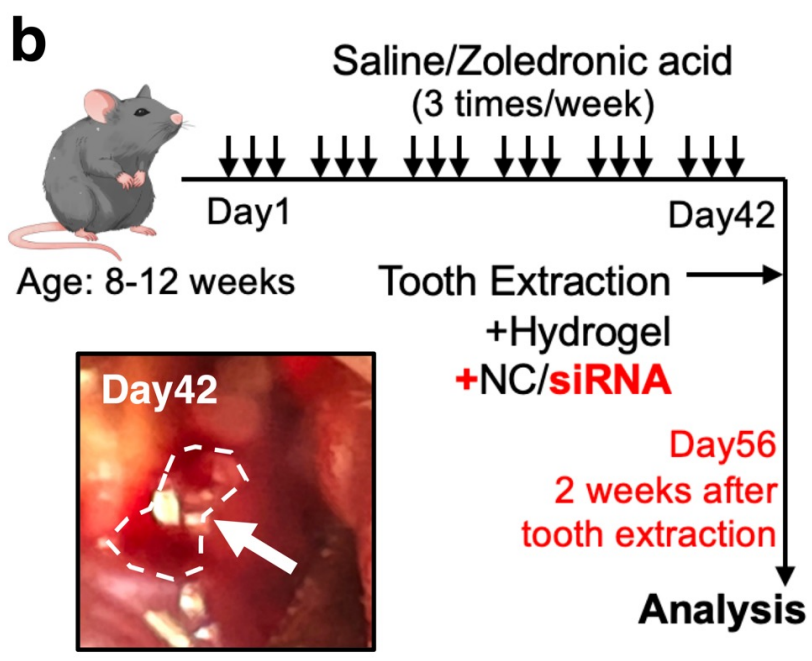

c

Name	Sequence (5'→3')	Modification
mCol2a1 siRNA sense	/i2OMeC/*/i2OMeC/*/i2OMeU/GG/i2OMeC/A/i2OMeU//i2OMeU/G/i2OMeC//i2OMeU/GG/i2OMeC//i2OMeU//i2OMeU//i2OMeU/AAA*/i2OMeU/*/i2OMeU/	3`Cholesteryl 9mU4*5mC
mCol2a1 siRNA antisense	/i2OMeU/*/i2OMeU/*/i2OMeU/AAAG/i2OMeC//i2OMeC/AG/i2OMeC/AA/i2OMeU/G/i2OMeC//i2OMeC/AGG*/i2OMeU/*/i2OMeU/	6mU4*5mC

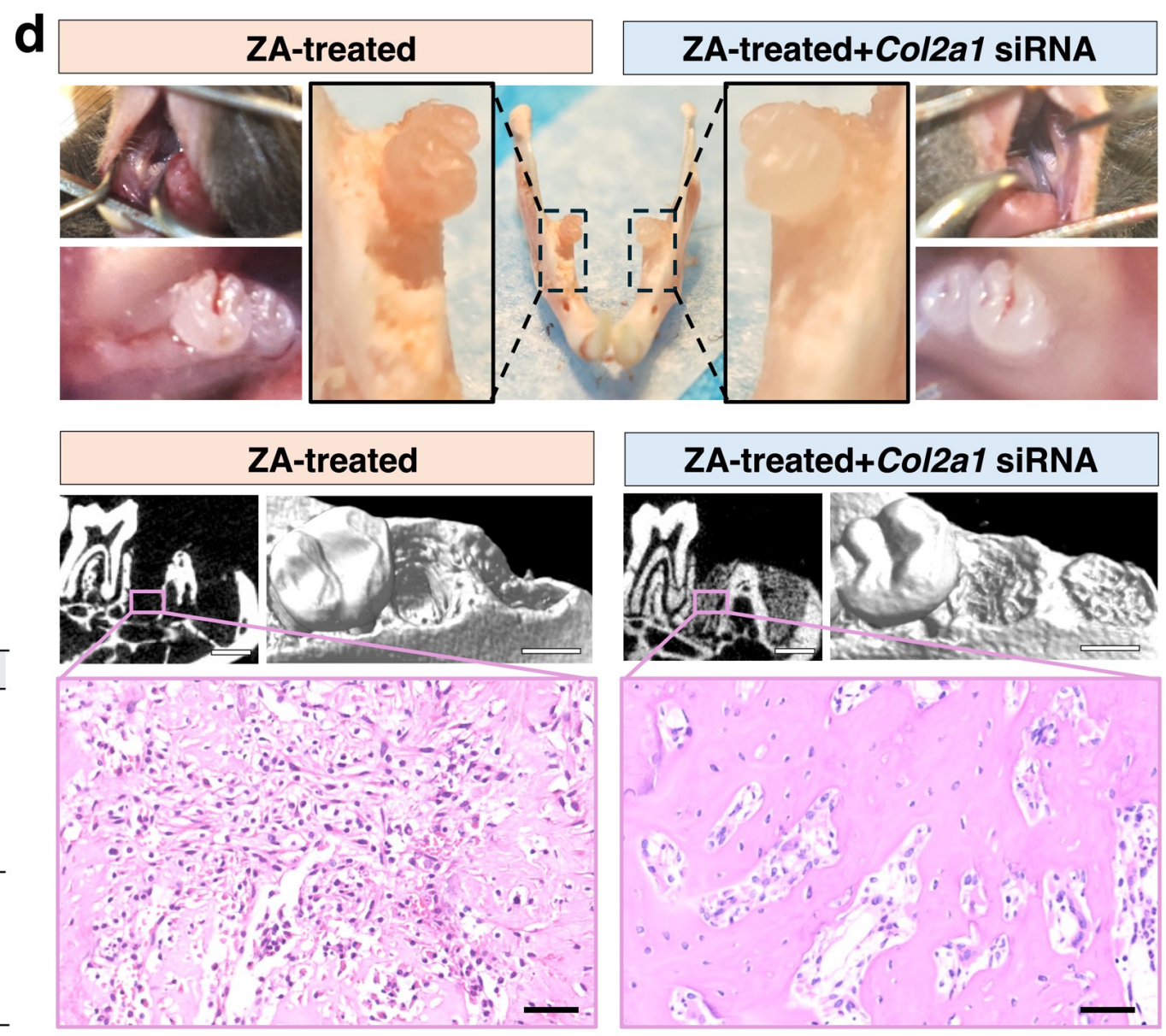

Supplementary Fig. 16: *In vitro Col2a1* expression and *in vivo* targeted therapy.

a Representative Calcein-AM/PI staining and RT-qPCR evaluation of *Col2a1* mRNA expression in HUVECs and LECs cultured in endothelial cell complete medium (ECM) or ECM containing zoledronic acid (ZA). **b** Schematic illustration of the local delivery of *Col2a1* siRNA-loaded hydrogels into the BRONJ extraction socket. **c** Sequences and modifications of the *Col2a1* siRNA sense and antisense strands. **d** Representative macroscopic images, micro-CT reconstructions, and H&E histological evaluations comparing the treated extraction sockets with the contralateral controls**. Scale bars:** white, 500 μm; black, 50 μm. ****P < 0.0001; *n=6.*

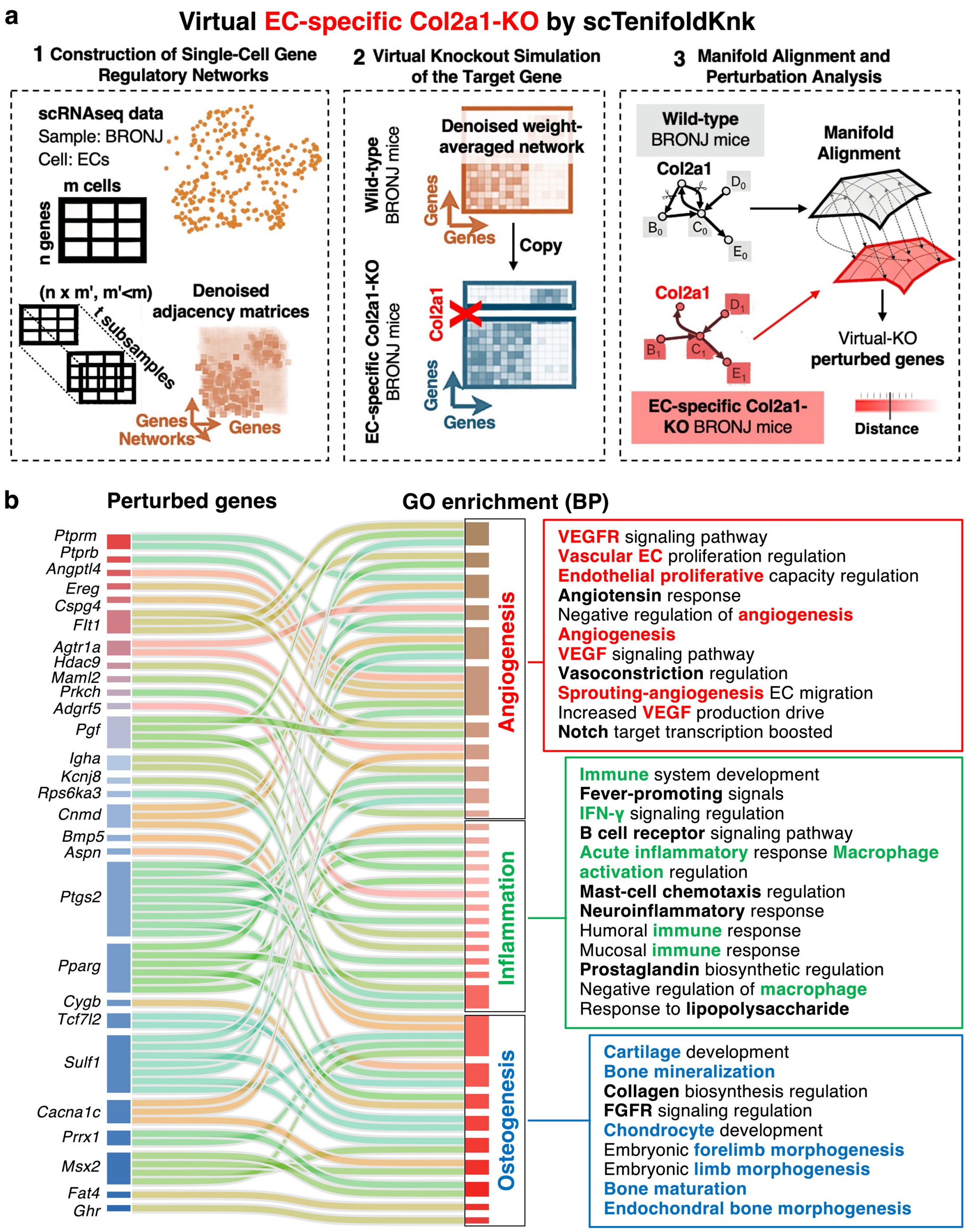

Supplementary Fig. 17: Virtual endothelial *Col2a1* knockout via scTenifoldKnk.

a Schematic illustration of the computational workflow for the virtual gene knockout analysis. **b** GO enrichment analysis of the significantly perturbed gene network following *Col2a1* deletion, highlighting key biological processes associated with angiogenesis, inflammation, and osteogenesis.

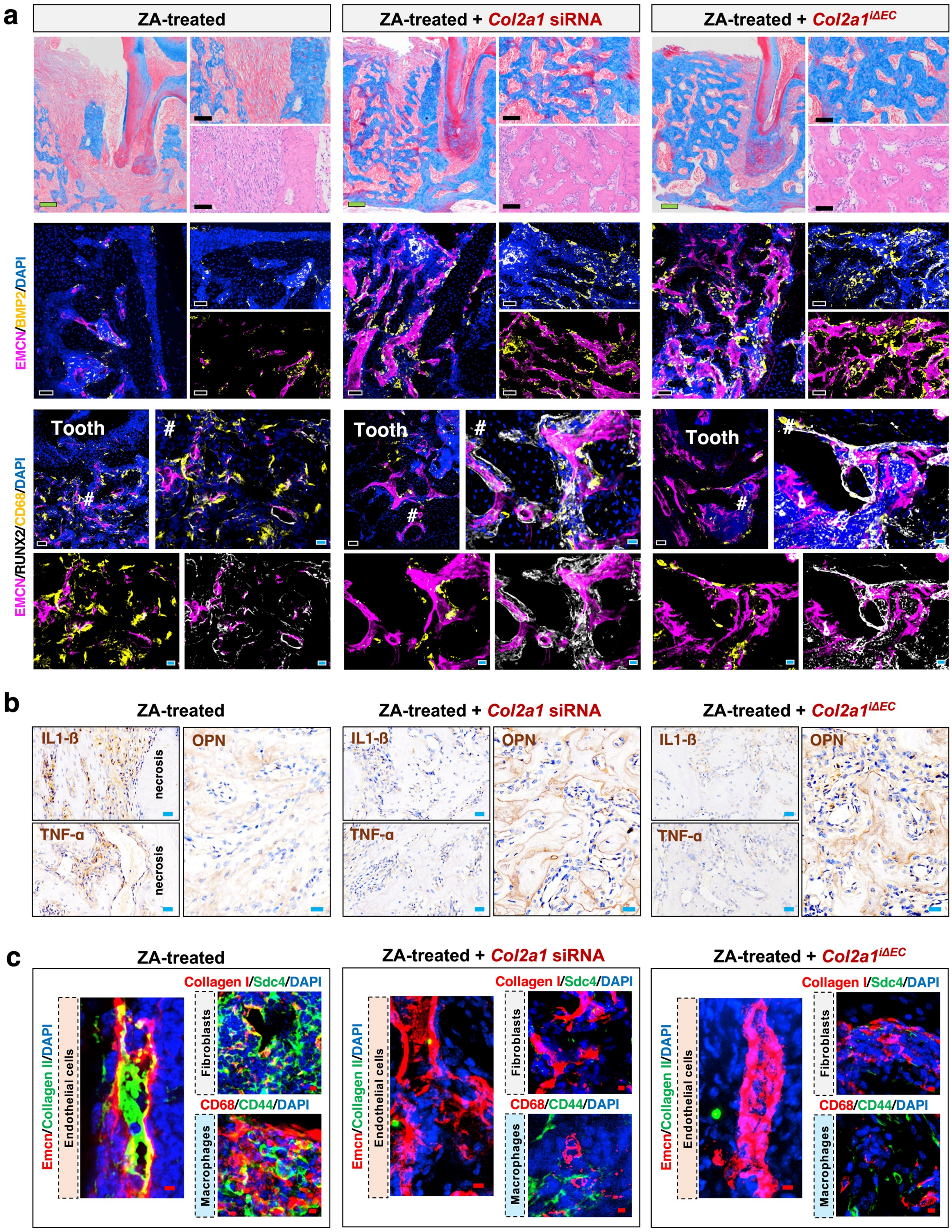

Supplementary Fig. 18: Effects of Col2a1 disruption on osteogenesis, inflammation, and ligand-receptor interactions in ZA-treated alveolar bone.

a Representative histological and immunofluorescence images of BMP2$^+$ and RUNX2$^+$ osteoblasts, as well as CD68$^+$ inflammatory cells, in the alveolar bone across the ZA-treated, ZA-treated+*Col2a1* siRNA, and ZA-treated+*Col2a1*$^{i\Delta EC}$ groups. **b** Immunohistochemical staining of pro-inflammatory cytokines (IL-1β and TNF-α) and the osteogenic marker osteopontin (OPN) in the alveolar bone. **c** Representative immunofluorescence images illustrating the expression of the endothelial Col2a1 ligand and its receptors, Sdc4 (on fibroblasts) and CD44 (on macrophages), across the three groups. **Scale bars**: green, 100 μm; black, 50 μm; blue, 20 μm; red, 5 μm.

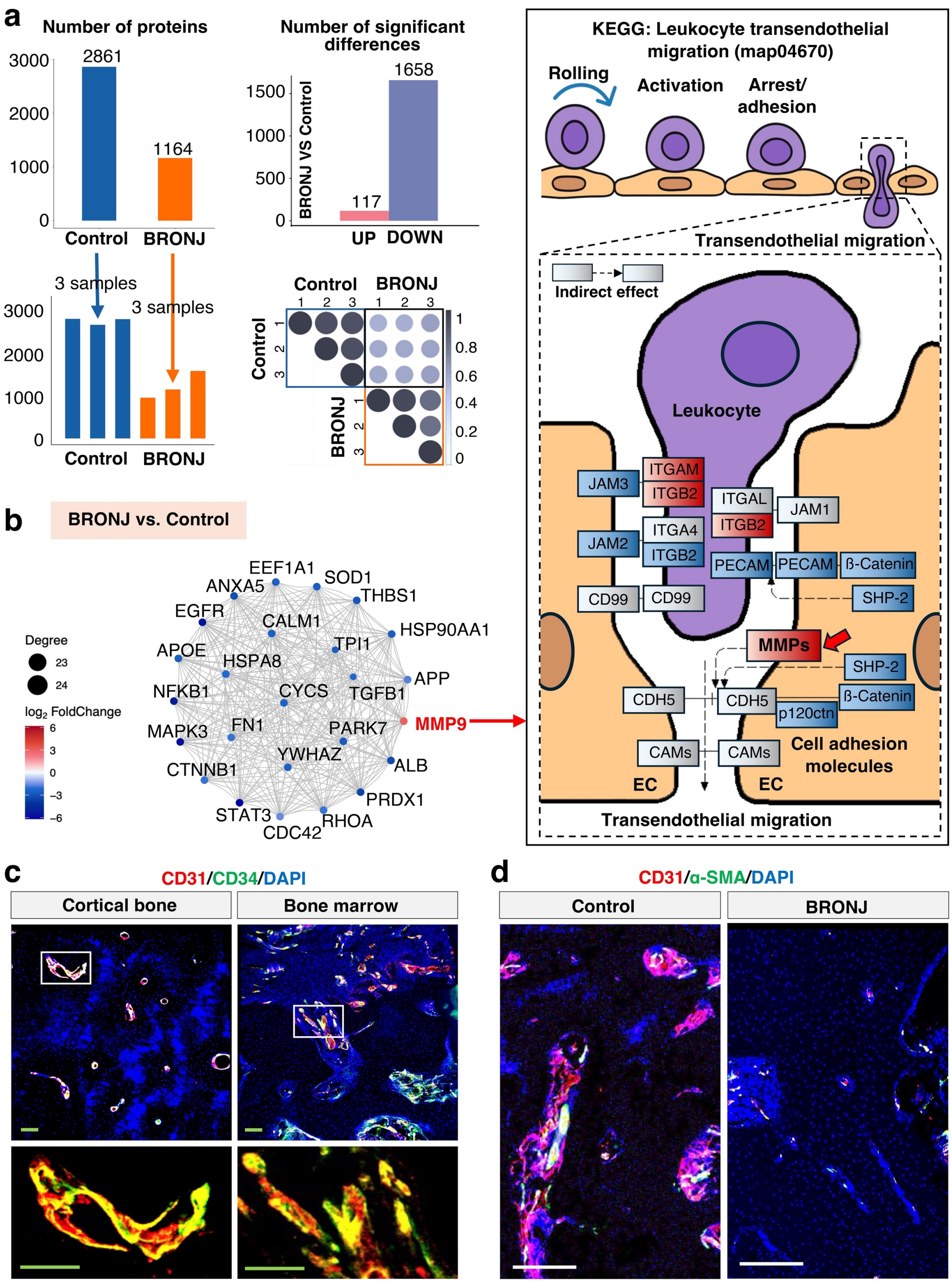

Supplementary Fig. 19: Proteomic and vascular-lymphatic pathology in human BRONJ.
a Comparative proteomic profiling of control and BRONJ mandibles showing protein counts, significantly altered proteins, and sample divergence. **b** PPI network of differentially expressed proteins with KEGG pathway mapping to leukocyte transendothelial migration. **c** 3D imaging of cortical bone and bone marrow immunostained for CD31 and CD34, with nuclear counterstaining by DAPI. **d** 3D imaging of BRONJ mandibles compared with controls, immunostained for CD31 and α-SMA, with DAPI counterstaining. **Scale bars:** white, 500 μm; green, 100 μm.

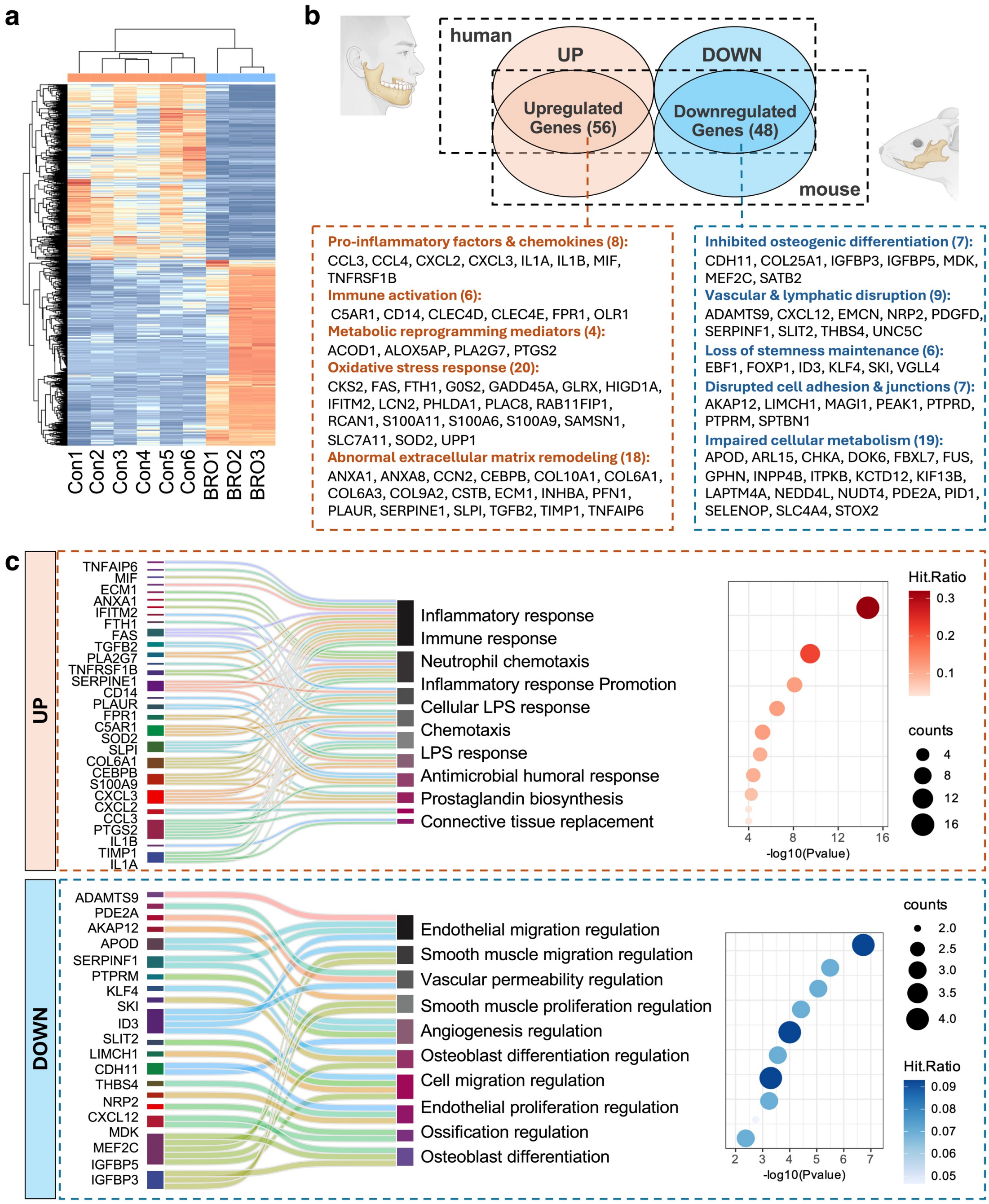

Supplementary Fig. 20: Cross-species transcriptomic analysis of BRONJ.

a Heatmap illustrating the differentially expressed genes (DEGs) between human control and BRONJ samples. **b** Identification of conserved transcriptomic signatures, displaying the intersection of commonly upregulated and downregulated genes between the human and mouse transcriptomes. **c** GO functional enrichment analysis of the cross-species conserved DEGs.

Supplementary Table 1

ITEM	COMPANY	CAT NO.
**Antibody**		
α-SMA	Sigma-Aldrich	C6198
HSPG2	Sigma-Aldrich	MAB1948P
Podocalyxin	R&D Systems	AF1556
Collagen IV	Abcam	ab236640
E-Selectin	Santa Cruz Biotechnology	SC-137054
GJA4	ThermoFisher Scientific	40-4300
Emcn	Santa Cruz Biotechnology	sc-65495
LYVE1	Abcam	ab14917
FABP4	R&D Systems	AF1443
FLT4	R&D Systems	AF743
F4/80	Bio-Rad Laboratories	MCA497GA
CD31	R&D Systems	AF3628
Collagen I	Abcam	ab34710
Collagen II	Abcam	ab34712
CD68	Abcam	ab125212
Sdc4	Santa Cruz Biotechnology	SC-12766
CD44	Abcam	ab6124
Il1ß	Servicebio	GB11113
TNF-α	Affinity	AF7014
OPN	Proteintech	22952-1-AP
IL6	Abcam	ab9324
RUNX2	Abcam	ab76956
CD34	ThermoFisher Scientific	MA1-10202
CHAD	ThermoFisher Scientific	PA5-53761
Donkey anti-Goat IgG (H + L) Alexa Fluor 546	ThermoFisher Scientific	A-11056
Donkey anti-Goat IgG (H + L) Alexa Fluor 488	ThermoFisher Scientific	A-11055
Donkey anti-Rat IgG (H + L) Alexa Fluor 488	ThermoFisher Scientific	A-21208
Donkey anti-Rabbit IgG (H + L) Alexa Fluor 647	ThermoFisher Scientific	A-31573
Donkey anti-Mouse IgG (H+L) Alexa Fluor 488	ThermoFisher Scientific	A-21202
**Reagent**		
Paraformaldehyde (PFA)	Sigma-Aldrich	P6148
Zoledronic acid	MCE	HY-13777
EDTA	Sigma-Aldrich	E6758
Sucrose	Sigma-Aldrich	V900116
Polyvinylpyrrolidone	Sigma-Aldrich	P5288

Gelatin	Sigma-Aldrich	G1890
Triton X-100	Sigma-Aldrich	T8787
Donkey serum	Solarbio	SL050
Methanol	Sigma-Aldrich	179337
Urea	Sigma-Aldrich	U5128
Glycerol	Sigma-Aldrich	G7893
FBS	Solarbio	S9020
DMSO	Sigma-Aldrich	472301
Isopropanol	Sigma-Aldrich	190764
Collagenase A	Merk	10103578001
ECi	Sigma-Aldrich	112372
PEGM	Sigma	447943
DAPI	Solarbio	C0060
H_2O_2	Sigma-Aldrich	HX0636
**Software**		
GraphPad Prism (version 9)	GraphPad Software, La Jolla, CA, USA	/
Imaris (version 10.2.0)	Bitplane	/
Imaris File converter (version 10.2.0)	Bitplane	/
Zen Black (version 3.1)	Zeiss (Jena, Germany)	/
Adobe Illustrator CC	Adobe	/
**Instruments**		
Low-profile blades	Leica	14035838382
Fluoromount-G	Invitrogen	00-4958-02
Cover glass	VWR	631-0135
Light-sheet microscope	Zeiss	Lightsheet 7
Spinning disk confocal microscope	Oxford Instruments	Andor Dragonfly 200
Micro-CT	SCANCO Medical AG	μCT50/μCT100
Digital slide scanner	Olympus	SLIDEVIEW VS200
