# Supplementary material for: Vascular and lymphatic dysregulation via non-EndoMT Col2a1 signaling in bisphosphonate-related osteonecrosis of the jaw
Source: Nat Commun. 2026 Sep 10;17:10715. doi: 10.1038/s41467-026-77692-w (PMC13658242; doi:10.1038/s41467-026-77692-w)
Supplement: Supplementary file 2 [file 41467_2026_77692_MOESM2_ESM.pdf]

nature portfolio

Corresponding author(s): Anjali P. Kusumbe, Junyu Chen

Last updated by author(s): Aug 17, 2026

Reporting Summary

Nature Portfolio wishes to improve the reproducibility of the work that we publish. This form provides structure for consistency and transparency in reporting. For further information on Nature Portfolio policies, see our Editorial Policies and the Editorial Policy Checklist.

Statistics

For all statistical analyses, confirm that the following items are present in the figure legend, table legend, main text, or Methods section.

n/a	Confirmed	
☐	☒	The exact sample size (*n*) for each experimental group/condition, given as a discrete number and unit of measurement
☐	☒	A statement on whether measurements were taken from distinct samples or whether the same sample was measured repeatedly
☐	☒	The statistical test(s) used AND whether they are one- or two-sided<br>*Only common tests should be described solely by name; describe more complex techniques in the Methods section.*
☐	☒	A description of all covariates tested
☐	☒	A description of any assumptions or corrections, such as tests of normality and adjustment for multiple comparisons
☐	☒	A full description of the statistical parameters including central tendency (e.g. means) or other basic estimates (e.g. regression coefficient) AND variation (e.g. standard deviation) or associated estimates of uncertainty (e.g. confidence intervals)
☐	☒	For null hypothesis testing, the test statistic (e.g. *F*, *t*, *r*) with confidence intervals, effect sizes, degrees of freedom and *P* value noted<br>*Give P values as exact values whenever suitable.*
☒	☐	For Bayesian analysis, information on the choice of priors and Markov chain Monte Carlo settings
☒	☐	For hierarchical and complex designs, identification of the appropriate level for tests and full reporting of outcomes
☒	☐	Estimates of effect sizes (e.g. Cohen's *d*, Pearson's *r*), indicating how they were calculated

Our web collection on statistics for biologists contains articles on many of the points above.

Software and code

Policy information about availability of computer code

Data collection	Raw single-cell and bulk RNA sequencing data were collected on the Illumina NovaSeq 6000 platform using the standard Illumina NovaSeq Control Software. High-resolution mass spectrometry spectra were acquired on a timsTOF Pro system using standard instrument control software. Fluorescence microscopy images were captured using the microscope (Oxford Instruments, Andor Dragonfly 200)'s standard operating software.
Data analysis	Raw single-cell sequencing data were processed using Cell Ranger (v7.0.1). Core single-cell downstream analysis, including normalization, dimensionality reduction, and module scoring, was performed using Seurat (v4.0.0). Doublets were identified and removed using DoubletFinder. Cell-cell communication was analyzed using the Cellchat R package (v1.1.3). Pseudotime trajectory inference was conducted via Monocle2 (v2.9.0). RNA velocity analysis was performed using velocyto.py and scVelo. Gene regulatory network inferences were executed through SCENIC (v1.2.4), RcisTarget (v1.10.0), and AUCell (v1.12.0), with regulon specificity scores computed using scFunctions. Single-cell metabolic pathway activity was quantified using scMetabolism (v0.2.1). In silico virtual knockout simulation was performed using the scTenifoldKnk R pipeline. For bulk RNA sequencing, reads were aligned using HISAT2, quantified via HTSeq-count, and analyzed for differential expression utilizing DESeq2 in R (v3.2.0). High-resolution proteomics spectra were processed using DIA-NN. Image quantification and 3D colocalization analyses were conducted using Imaris (v10.2) and Fiji (v2.14.0/1.54f). All statistical analyses were performed using GraphPad Prism (v9.0).

For manuscripts utilizing custom algorithms or software that are central to the research but not yet described in published literature, software must be made available to editors and reviewers. We strongly encourage code deposition in a community repository (e.g. GitHub). See the Nature Portfolio guidelines for submitting code & software for further information.

Data

Policy information about availability of data

All manuscripts must include a data availability statement. This statement should provide the following information, where applicable:

- Accession codes, unique identifiers, or web links for publicly available datasets
- A description of any restrictions on data availability
- For clinical datasets or third party data, please ensure that the statement adheres to our policy

Source data are provided in this paper. The remaining data are available within the Article, Supplementary Information, and Source Data file. Sequence data are deposited in the NCBI Gene Expression Omnibus under accession numbers GSE295106. The mass spectrometry proteomics data have been deposited to the ProteomeXchange Consortium (https://proteomecentral.proteomexchange.org) via the iProX partner repository with the dataset identifier PXD068365.

Research involving human participants, their data, or biological material

Policy information about studies with human participants or human data. See also policy information about sex, gender (identity/presentation), and sexual orientation and race, ethnicity and racism.

Reporting on sex and gender	Human samples were collected from 18 individuals. The control group comprised 9 donors (7 females, 2 males) with a mean age of 73.0 ± 8.9 years. The BRONJ group consisted of 9 patients (7 females, 2 males) with a mean age of 74.6 ± 4.7 years. Biological sex (determined via medical records) was recorded, but gender is not applicable to this study. Sex-based analyses were not performed due to the limited sample size (Total human n<20), which lacks sufficient statistical power for sex-stratification. The study focused on fundamental, conserved molecular and cellular mechanisms of BRONJ pathogenesis, which are not anticipated to be sex-dependent.
Reporting on race, ethnicity, or other socially relevant groupings	Not applicable. Data on race, ethnicity, or other socially relevant groupings were neither collected nor analyzed. This study investigates fundamental conserved molecular and cellular mechanisms of BRONJ pathogenesis utilizing cell lines, animal models, and a small human tissue cohort for mechanistic validation. Socially constructed variables are not biologically relevant to the fundamental endothelial and bone remodeling pathways evaluated in this study, and thus were not included in the study design or statistical adjustments.
Population characteristics	A total of 18 human subjects were included to obtain mandibular bone tissues, comprising a healthy control group (n = 9) and a BRONJ group (n = 9). Age and genotypic information are not applicable as covariates (See above)<br>Control Group: Individuals requiring routine tooth extraction (e.g., impacted third molars or orthodontic extractions) with no history of bone metabolic diseases, radiotherapy, or antiresorptive/antiangiogenic therapies.<br>BRONJ Group: Patients clinically diagnosed according to AAOMS guidelines. The primary underlying disease requiring antiresorptive therapy included osteoporosis or bone metastases. All patients had a documented history of bisphosphonate treatment prior to the onset of jaw necrosis.
Recruitment	Participants were recruited from patients admitted to the West China Hospital of Stomatology, Sichuan University. The healthy control group consisted of individuals scheduled for routine tooth extractions, while the BRONJ group comprised patients undergoing surgical sequestrectomy for necrotic jawbone tissue.<br>Potential biases and impact: A potential single-center selection bias exists, as all participants were recruited from a single tertiary referral hospital. This could theoretically skew the cohort towards patients with specific regional demographics or more severe clinical profiles. However, because the primary objective of this study is to investigate fundamental, highly conserved cellular and molecular mechanisms within the bone microenvironment (rather than evaluating population-level epidemiological outcomes), this geographical and institutional selection bias is highly unlikely to impact the validity, reproducibility, or generalizability of our core biological findings.
Ethics oversight	All animal experiments complied with local ethical guidelines and regulations and received approval from the Research Ethics Committee of West China Hospital of Stomatology (Approval No. WCHSIRB-D-2023-245). All experiments using these human samples received approval from the Research Ethics Committee of West China Hospital of Stomatology (Approval No. WCHSIRB-D-2025-392). Written informed consent was obtained from all participants.

Note that full information on the approval of the study protocol must also be provided in the manuscript.

Field-specific reporting

Please select the one below that is the best fit for your research. If you are not sure, read the appropriate sections before making your selection.

- [x] Life sciences
- [ ] Behavioural & social sciences
- [ ] Ecological, evolutionary & environmental sciences

For a reference copy of the document with all sections, see nature.com/documents/nr-reporting-summary-flat.pdf

Life sciences study design

All studies must disclose on these points even when the disclosure is negative.

Sample size	No statistical methods were used to predetermine sample sizes. Instead, they were chosen based on established empirical standards for high-resolution imaging and multi-omics in bone biology.Rationale for sufficiency:scRNA-seq: Statistical power is driven by the massive single-cell

resolution (34,350 high-quality cells analyzed), providing robust evidence for transcriptional shifts. Imaging & Histology: Using n = 3–6 biological replicates per group adequately accounted for baseline variability , which is empirically supported by consistent statistical significance ($P < 0.05$) across independent validation assays. Human Validation: A cohort of n = 9 human specimens per group was sufficient to capture key transcriptomic, proteomic, and structural imaging changes , successfully cross-validating the core pathological mechanisms observed in mice.

Data exclusions	No human or animal samples were excluded from the study. Pre-established data-level filtering criteria were strictly applied for quality control during multi-omics analysis. Specifically, single-cell RNA sequencing data excluded cells failing to meet UMI/gene count thresholds (median ± 2 MADs), exceeding a 20% mitochondrial fraction, or identified as doublets by DoubletFinder prior to downstream analysis. For high-resolution LC-MS/MS proteomics, proteins were retained only if supported by at least one unique peptide and at least two valid values in 50% or more of the samples in at least one group.
Replication	All attempts at experimental replication were successful. Experimental findings were systematically verified using multiple independent biological replicates (typically n = 3 to 6 per group), as detailed across independent functional, morphological, and validation assays.
Randomization	Mice were randomly assigned to the experimental (zoledronate-treated) and vehicle control groups during the establishment of the murine BRONJ model. Randomization was not applicable to the human specimens, as participants were allocated into the healthy control or BRONJ group strictly based on pre-existing clinical diagnoses according to the AAOMS guidelines and antiresorptive treatment histories.
Blinding	Investigator blinding was not performed during group allocation or sample collection because the clinical and histopathological presentations of healthy controls and necrotic BRONJ jawbones were highly distinct and readily identifiable. To minimize potential observer bias, downstream morphometric quantifications (including micro-CT structural profiling and 3D fluorescence colocalization or masking in Imaris and Fiji) and bioinformatic pipelines were executed using automated, uniform, and pre-set software criteria.

Reporting for specific materials, systems and methods

We require information from authors about some types of materials, experimental systems and methods used in many studies. Here, indicate whether each material, system or method listed is relevant to your study. If you are not sure if a list item applies to your research, read the appropriate section before selecting a response.

Materials & experimental systems

n/a	Involved in the study
☐	☒ Antibodies
☐	☒ Eukaryotic cell lines
☒	☐ Palaeontology and archaeology
☐	☒ Animals and other organisms
☒	☐ Clinical data
☒	☐ Dual use research of concern
☒	☐ Plants

Methods

n/a	Involved in the study
☒	☐ ChIP-seq
☒	☐ Flow cytometry
☒	☐ MRI-based neuroimaging

Antibodies

Antibodies used	Primary antibodies targeting vascular, lymphatic, bone matrix, and inflammatory components include: anti-α-SMA (Sigma-Aldrich, Cat# C6198), anti-HSPG2 (Sigma-Aldrich, Cat# MAB1948P), anti-Podocalyxin (R&D Systems, Cat# AF1556), anti-Collagen IV (Abcam, Cat# ab236640), anti-E-Selectin (Santa Cruz Biotechnology, Cat# SC-137054), anti-GJA4 (ThermoFisher Scientific, Cat# 40-4300), anti-Emcn (Santa Cruz Biotechnology, Cat# sc-65495), anti-LYVE1 (Abcam, Cat# ab14917), anti-FABP4 (R&D Systems, Cat# AF1443), anti-FLT4 (R&D Systems, Cat# AF743), anti-F4/80 (Bio-Rad Laboratories, Cat# MCA497GA), anti-CD31 (R&D Systems, Cat# AF3628), anti-Collagen I (Abcam, Cat# ab34710), anti-Collagen II (Abcam, Cat# ab34712), anti-CD68 (Abcam, Cat# ab125212), anti-IL6 (Abcam, Cat# ab9324), anti-RUNX2 (Abcam, Cat# ab76956), anti-CD34 (ThermoFisher Scientific, Cat# MA1-10202), and anti-CHAD (ThermoFisher Scientific, Cat# PA5-53761). Fluorophore-conjugated secondary antibodies used for multi-channel visualization were all obtained from ThermoFisher Scientific, consisting of: Donkey anti-Goat IgG (H + L) Alexa Fluor 546 (Cat# A-11056), Donkey anti-Goat IgG (H + L) Alexa Fluor 488 (Cat# A-11055), Donkey anti-Rat IgG (H + L) Alexa Fluor 488 (Cat# A-21208), Donkey anti-Rabbit IgG (H + L) Alexa Fluor 647 (Cat# A-31573), and Donkey anti-Mouse IgG (H+L) Alexa Fluor 488 (Cat# A-21202). Primary antibodies, secondary antibodies, and DAPI were used at dilutions of 1:150, 1:300, and 1:1000, respectively. Clone names for monoclonal reagents are designated by the respective manufacturers, and batch-specific lot numbers for all primary and secondary antibodies were tracked systematically in internal laboratory logs and are available from the corresponding author upon reasonable request.
Validation	For the characterization of human clinical specimens, target-specific or cross-species validation for Homo sapiens was utilized for CD34 (Cat# MA1-10202), CD31 (Cat# AF3628), LYVE1 (Cat# ab14917), CHAD (Cat# PA5-53761), Collagen I (Cat# ab34710), Collagen II (Cat# ab34712), Collagen IV (Cat# ab236640), α-SMA (Cat# C6198), E-Selectin (Cat# SC-137054), GJA4 (Cat# 40-4300), IL6 (Cat# ab9324), RUNX2 (Cat# ab76956), and HSPG2 (Cat# MAB1948P) to accurately map human mandibular tissue environments. For the experimental evaluation of murine disease models, validated reactivity for Mus musculus was leveraged for Emcn (Cat# sc-65495; explicitly verified by the manufacturer as reactive in rodents and non-reactive in humans), Podocalyxin (Cat# AF1556), FLT4 (Cat# AF743), FABP4 (Cat# AF1443), F4/80 (Cat# MCA497GA), and CD68 (Cat# ab125212), alongside the cross-reactive markers CD31, LYVE1, CHAD, α-SMA, E-Selectin, GJA4, IL6, RUNX2, Collagen I, Collagen II, and Collagen IV. These species-specific applications are heavily supported by manufacturer portals and extensive peer-reviewed citations, and the operational specificity of each antibody within the bone marrow, microvascular, and immune niches was internally cross-validated within this manuscript by the absolute concordance between in situ immunostaining patterns and corresponding cell-type transcript/protein expression profiles from our scRNA-seq and proteomic datasets.

Eukaryotic cell lines

Policy information about cell lines and Sex and Gender in Research

Cell line source(s)	The human eukaryotic cell lines used in this study were PUMC-HUVEC-T1, purchased from Procell (Cat# CL-0675) , and hLECs, purchased from IMMOCELL (Cat# IM-H480). Both lines are derived from human tissues. The biological sex of the donors for these commercial cell lines was not specified by the respective manufacturers.
Authentication	Both cell lines were authenticated by their respective manufacturers prior to purchase. Cell identity was verified using Short Tandem Repeat (STR) profiling (for the human PUMC-HUVEC-T1 line) and characteristic endothelial morphology alongside specific marker expression analysis (for both PUMC-HUVEC-T1 and hLECs).
Mycoplasma contamination	All cell lines utilized in this study were routinely tested and confirmed to be completely negative for mycoplasma contamination before use in experimental procedures.
Commonly misidentified lines (See ICLAC register)	None of the cell lines used in this study are listed in the International Cell Line Authentication Committee (ICLAC) register of commonly misidentified cell lines.

Animals and other research organisms

Policy information about studies involving animals; ARRIVE guidelines recommended for reporting animal research, and Sex and Gender in Research

Laboratory animals	The study utilized female C57BL/6J mice aged 8–12 weeks, purchased from Dashuo Biotechnology Co., Ltd. (Chengdu, China). Transgenic and reporter strains, including Cdh5-2A-CreERT2 (stock no. NM-KI-200173), Rosa26-LSL-td-Tomato (stock no. NM-KI 225042), and Col2a1fl/fl (stock no. NM-CKO-240983), were obtained from Shanghai Southern Model Biotechnology Co., Ltd. (China). Cdh5-Cre-Tg mice (stock no. J-017968) were obtained from the Jackson Laboratory (USA). Genetic lineage tracing was performed using crossed Cdh5-2A-CreERT2 × Rosa26-LSL-td-Tomato mice, and the endothelial-specific conditional knockout model (Col2a1iΔEC) was generated by crossing Cdh5-Cre-Tg and Col2a1fl/fl mice. All mice were housed in a specific pathogen-free (SPF) facility maintained at 22 ± 2 °C with 40–60% humidity under a standard 12-hour light/12-hour dark cycle. Mice were fed a standard laboratory rodent chow diet (comprising approximately 18% protein and 4% fat; Dashuo Biotechnology, Chengdu, China) and provided with autoclaved water ad libitum.
Wild animals	The study did not involve wild animals.
Reporting on sex	Sex-based statistical analyses were not performed because the study exclusively utilized a single sex (female mice) to ensure baseline physiological homogeneity.
Field-collected samples	The study did not involve samples collected from the field.
Ethics oversight	All animal experimental protocols complied with local ethical guidelines and regulations and received formal institutional approval from the Research Ethics Committee of West China Hospital of Stomatology under Approval No. WCHSIRB-D-2023-245.

Note that full information on the approval of the study protocol must also be provided in the manuscript.

Plants

Seed stocks	Not applicable.
Novel plant genotypes	Not applicable.
Authentication	Not applicable.
