# Supplementary material for: Vascular and lymphatic dysregulation via non-EndoMT Col2a1 signaling in bisphosphonate-related osteonecrosis of the jaw
Source: Nat Commun. 2026 Sep 10;17:10715. doi: 10.1038/s41467-026-77692-w (PMC13658242; doi:10.1038/s41467-026-77692-w)
Supplement: Supplementary file 3 [file 41467_2026_77692_MOESM3_ESM.pdf]

nature portfolio

Peer Review File

Vascular and Lymphatic Dysregulation via Non-EndoMT Col2a1 Signaling in Bisphosphonate-Related Osteonecrosis of the Jaw

Corresponding Author: Professor Junyu Chen

Parts of this Peer Review File have been redacted as indicated to remove third-party material.

This file contains all reviewer reports in order by version, followed by all author rebuttals in order by version.

Version 0:

Reviewer comments:

Reviewer #1

(Remarks to the Author)
This manuscript investigates endothelial remodeling in bisphosphonate-related osteonecrosis of the jaw (BRONJ) in mice and humans using advanced tissue-clearing and single-cell analyses. By administering bisphosphonate to mice, the authors demonstrate a reduction in vascular structures using tissue clearing and characterize remodeling of endothelial and stromal subsets by single-cell RNA-seq. They further propose potential cell–cell interaction mechanisms using CellChat analysis. In human BRONJ samples, the authors apply proteomic profiling and tissue clearing to assess microenvironmental changes and vascular remodeling.
Overall, the study combines multiple complementary approaches and is technically sound. However, the work remains largely descriptive, and several of the proposed mechanisms are not experimentally validated, limiting the strength of the conclusions. The following points should be addressed to enhance the rigor and impact of the study.

Major Concerns
1. The authors highlight Col2a1 upregulation as a key driver of tissue remodeling in BRONJ, even featuring it in the title and abstract. They propose the importance of the Col2a1–CD44/SDC4 axis based on CellChat analysis. However, there is insufficient experimental evidence to support this hypothesis. Functional assays using Col2a1 conditional knockout (KO) mice are necessary to test whether Col2a1 deficiency affects BRONJ pathology. In histology (Fig. 6e, h), other cell types appear to express type II collagen more strongly than endothelial cells. The authors should clarify which cells these are, as their Col2a1 expression may be more relevant to the pathology. Generation of EC-specific Col2a1 KO mice would directly address this question.
2. It would strengthen the work to perform single-cell RNA-seq on human BRONJ samples (endothelial and stromal compartments) and compare these data with the mouse datasets.
3. The authors speculate that zoledronate-induced Col2a1 upregulation in ECs might influence fibroblasts or macrophages expressing SDC4 or CD44. This remains highly speculative without direct evidence. In vitro assays using endothelial (BECs and LECs) treated with zoledronate should be performed to test whether Col2a1 is indeed upregulated in ECs. Moreover, EC remodeling may be a secondary effect driven by altered stromal activity and tissue remodeling. In CellChat, ECs were always defined as senders, which introduces bias. The authors should also analyze other cell types as senders to explore reciprocal interactions. Tools such as NicheNet could be also helpful for this.
4. In line 97, the authors describe three vascular subtypes defined by Emcn and Podocalyxin. The manuscript should clearly show where these subtypes are located in the single-cell datasets.
5. Lymphatic structures in the tooth–jawbone region have not, to my knowledge, been previously characterized. It would therefore be valuable to visualize lymphatics in the cleared tissue to determine their spatial distribution. Additional LEC markers such as PROX1 or PDPN should be used, since LYVE1 is not specific for lymphatic endothelium, as the authors themselves note.
6. The authors suggest that BRONJ induces pro-inflammatory responses in ECs and macrophages. However, Fig. 2c shows minimal immune infiltration, which would suggest a non-inflammatory state. The authors need to explain this discrepancy and reconcile histological findings with transcriptional data.
7. In the endothelial single-cell dataset, capillary ECs seem to be absent. Do the authors believe this tissue lacks capillaries,

were these cells lost during processing or filtering or annotated wrongly? Clarification is required.
8. In Fig. 5B, the dataset includes a population labeled as “progenitor macrophages.” The authors should clarify what these cells represent. Are they monocytes or proliferative macrophages? This point needs explanation, as the presence of progenitor macrophages in this tissue is unexpected.

Reviewer #2

(Remarks to the Author)
Major comments:
Based on a novel imaging approach of the intact tooth–jaw structure and in-depth scRNA-seq analyses, the authors identified four vascular subtypes, eight fibroblast subtypes, and two macrophage subtypes. In BRONJ, there was a significant depletion of all vascular subtypes except sinusoidal endothelial cells (SECs), whereas LRRC15⁺ and CHAD⁺ fibroblasts and IL6⁺ macrophages selectively expanded. Furthermore, the COL2A1–CD44/SDC4 axis was highlighted as a BRONJ-specific endothelial-to-fibroblast/macrophage signaling pathway. Overall, this was a compelling study that delineated vascular and lymphatic dysregulation via non-EndoMT mechanisms in BRONJ. Nevertheless, the work remains incomplete without functional evidence. To substantiate the proposed role of the COL2A1–CD44/SDC4 axis, targeted genetic or pharmacologic perturbations in appropriate BRONJ models are required.

Specific comments:
1. In Figure 1d, the authors identified three vascular subtypes, Emcn-low/Podcalyxin-low, Emcn-high/Podcalyxin-high, and Emcn-high/Podcalyxin-low, in the mandibular alveolar bone. However, the relative abundance of these subtypes remains unclear. Furthermore, the relationship between these Emcn/Podocalyxin-defined subtypes and the four vascular subtypes described in Figure 3a should be clarified. In addition, since Podcalyxin-expressing endothelial cells in long-bone marrow have not been clearly characterized, it would be helpful to clarify the similarities and differences in vascular subtypes between long bone and mandibular alveolar bone.
2. In Figure 1g and 1h, the authors showed a reduction of type H vessels, arteries, and lymphatic vessels in the BRONJ group. However, the spatial relationship between the decreased vessels and the necrotic region remains unclear.
3. In Figure 2, the expression of Sele was decreased in BRONJ. The underlying mechanism remains unclear.

Reviewer #3

(Remarks to the Author)
This study aims to provide a valuable multimodal atlas of bisphosphonate-related osteonecrosis of the jaw (BRONJ) integrating whole-organ imaging, single-cell transcriptomics, and proteomics in mouse and human mandibles. Using bioinformatic analysis, the authors identify endothelial signaling alterations as central features of disease pathogenesis, particularly elevated Col2a1 expression in endothelial cells, which is predicted to act as a paracrine hub through the COL2A1-CD44/SDC4 axes. Although the integrative approach is technically robust and the cross-species validation compelling, the data is still descriptive at the current stage and is only sufficient to support correlation, not a causal relationship, between the vascular changes and bone defects. In addition, the authors do not appear to have carefully considered the temporal factors relevant to the progression of BRONJ. Both the vascular alterations and single-cell analyses should be interpreted as contributing mechanistic factors in the context of early disease development, before the phenotype is fully established (e.g. day 56 in Fig. 1f). Mechanistically, because CellChat predictions are based on ligand and receptor expression rather than actual signaling activity, the authors should also examine whether the COL2A1-CD44/SDC4 signaling network is indeed activated in vivo and verify its functional role in BRONJ progression. Overall, the study is not mechanistic and preliminary.

1. The authors have established a whole-mount imaging protocol optimized for exogenous antibody labeling of the intact tooth-jawbone structure (lines 82–86). This method will be very valuable for the field. However, the red signals appear to be non-specific particularly within the dental pulp, most notably in in Figure 1 B (α-SMA) and 1C (CD31).
2. Many figures/data are missing information on the timing of sample collection. Please notate the time points consistently across all figures in the manuscript.
3. Lines 96–97: The authors reported 3 vascular subtypes with distinct spatial distributions within the mandibular alveolar bone, based on the co-expression patterns of Emcn and Podocalyxin. Could the authors explain the rationale for selecting these two specific markers to evaluate vascular heterogeneity? In addition, please clarify the spatial distribution differences between the 3 identified vascular subtypes (EmcnloPodocalyxinhi, EmcnhiPodocalyxinhi, and EmcnhiPodocalyxinlo) in the mandibular alveolar bone.
4. In Figures 1g, 1h, and S1f, the whole-organ imaging nicely demonstrates vascular and lymphatic attenuation in BRONJ. However, to support the conclusion that endothelial cells play a central role in the pathogenic mechanism of BRONJ, the authors should clarify whether these vascular changes represent a cause rather than a consequence of bone necrosis. While it is understandable that time-course analysis may not be feasible for human samples across disease stages, the authors should take advantage of this mouse model to perform a time-course analysis following zoledronate treatment/tooth extraction to determine when vascular defects first arise and whether they precede necrotic changes.
5. A key piece of missing information concerns the timing of the single-cell transcriptomic profiling of healthy and BRONJ mouse mandibles. The authors must specify how long after zoledronate treatment/tooth extraction the samples were collected. Moreover, to elucidate the mechanisms contributing to bone defects, the single-cell experiments should be

performed at an earlier stage when molecular changes are still initiating these pathological changes, rather than at day 56 (4 weeks post-zoledronate treatment) as shown in Figure 1f, when necrotic bone defects are already well developed. If performed at earlier stages, would CellChat analysis still reveal similar significantly enhanced endothelial cell-to-fibroblast/microphage signaling in BRONJ?
6. Using bioinformatic analysis (CellChat), the authors report elevated Col2a1 expression in endothelial cells and propose that it acts through the COL2A1-CD44/SDC4 axis in fibroblasts and macrophages, likely promoting extracellular matrix remodeling and inflammation. Because CellChat predictions are based on ligand and receptor expression rather than actual signaling activity, the authors should validate these findings by examining in vivo gene expression changes of the COL2A1-CD44/SDC4 axis ligands or receptors and, more importantly, the corresponding signaling readouts in mouse and human BRONJ tissue sections. The authors should then test whether restoring the COL2A1-CD44/SDC4 axis can rescue the bone defects by modulating matrix remodeling and inflammation to substantiate their proposed functional role.

Version 1:

Reviewer comments:

Reviewer #1

(Remarks to the Author)
The authors adequately responded to my concerns. I have no more comments.

Reviewer #2

(Remarks to the Author)
The authors have sufficiently addressed the reviewers' concerns. I have no further comments.

Reviewer #3

(Remarks to the Author)
In this revision, the authors have performed additional experiments, improved data quality, and added experimental procedural details, all of which significantly strengthen the study. However, some concerns remain and will need to be clarified to make the conclusions and findings more solid:

Q3: Defining the in vivo locations of the three vascular subtypes by Emcn/Podocalyxin co-expression is a valuable finding. However, Supplementary Fig. 8b shows only high-magnification images of the individual expression patterns, making the anatomical distribution hard to appreciate. A lower-magnification image of the co-expression patterns, with higher-magnification images as insets, comparable to the schematic in Supplementary Fig. 8b, would be necessary.
Q4: Quantitative statistical analysis, in addition to the images, is needed to evaluate the change in vascularity in Supplementary Fig. 2b.
Q5: It is understandable that the authors may not be able to perform additional scRNA-seq experiments at earlier time points. Since the authors already have time-course sections from day 1 to day 14 post tooth extraction, could the authors still try to evaluate whether the endothelial cell-to-fibroblast/macrophage signaling identified by CellChat is already significantly enhanced before definitive histological bone necrosis becomes evident? This would help establish that endothelial cell-to-fibroblast/macrophage signaling is a contributing factor to the necrosis rather than a downstream event following necrosis.
Q6: Since the authors propose that endothelial-derived Col2a1 acts through the COL2A1–CD44/SDC4 axis in fibroblasts and macrophages, can they confirm that the upregulation of Col2a1 and its receptors (Cd44 and Sdc4) occurs in these fibroblasts and macrophages, preferably using histological sections?
More importantly, the two powerful in vivo tools the authors have used—localized Col2a1-targeting siRNA hydrogel delivery and endothelial-specific Col2a1 conditional knockout mice—should be explored further to dissect the mechanism of the COL2A1–CD44/SDC4 axis in BRONJ. For example, does reduced Col2a1 restore the expression of its receptors Cd44/Sdc4 and, in addition to vascular morphological recovery, eventually reduce necrosis (e.g., by histology)? Moreover, since the authors bioinformatically predict that Col2a1 deletion perturbs gene networks governing angiogenesis, inflammation, and osteogenesis (Supplementary Fig. 16), could they validate whether this prediction holds in vivo using the existing histology sections for both localized silencing and genetic deletion (Figure 6h)?

Version 2:

Reviewer comments:

Reviewer #3

(Remarks to the Author)
The authors have performed additional experiments to address my previous comments. These new data have significantly strengthened the study, and I have no further comments

Open Access This Peer Review File is licensed under a Creative Commons Attribution 4.0 International License, which permits use, sharing, adaptation, distribution and reproduction in any medium or format, as long as you give appropriate credit to the original author(s) and the source, provide a link to the Creative Commons license, and indicate if changes were made.
In cases where reviewers are anonymous, credit should be given to 'Anonymous Referee' and the source.
The images or other third party material in this Peer Review File are included in the article's Creative Commons license, unless indicated otherwise in a credit line to the material. If material is not included in the article's Creative Commons license and your intended use is not permitted by statutory regulation or exceeds the permitted use, you will need to obtain permission directly from the copyright holder.
To view a copy of this license, visit https://creativecommons.org/licenses/by/4.0/

Point-by-point response

We sincerely appreciate the editor and the reviewers' time and insightful comments. We have carefully revised the manuscript to address all the concerns raised and believe that the revised version has been significantly improved. All modifications are highlighted in yellow, and our point-by-point responses to the reviewers' comments are provided below.

Response to Reviewer 1:

This manuscript investigates endothelial remodeling in bisphosphonate-related osteonecrosis of the jaw (BRONJ) in mice and humans using advanced tissue-clearing and single-cell analyses. By administering bisphosphonate to mice, the authors demonstrate a reduction in vascular structures using tissue clearing and characterize remodeling of endothelial and stromal subsets by single-cell RNA-seq. They further propose potential cell–cell interaction mechanisms using CellChat analysis. In human BRONJ samples, the authors apply proteomic profiling and tissue clearing to assess microenvironmental changes and vascular remodeling. Overall, the study combines multiple complementary approaches and is technically sound. However, the work remains largely descriptive, and several of the proposed mechanisms are not experimentally validated, limiting the strength of the conclusions. The following points should be addressed to enhance the rigor and impact of the study.

Response: We sincerely appreciate your thorough assessment evaluation of our study and your highly constructive feedback regarding the manuscript. To further elevate the mechanistic rigor of our study, we have performed extensive *in vitro* and *in vivo* functional validation experiments to confirm the bioinformatic predictions regarding the *Col2a1-Sdc4/Cd44* signaling axis. We have completed the modifications you suggested, and the specific changes are as follows:

Q1. The authors highlight Col2a1 upregulation as a key driver of tissue remodeling in BRONJ, even featuring it in the title and abstract. They propose the importance of the Col2a1–CD44/SDC4 axis based on CellChat analysis. However, there is insufficient experimental evidence to support this hypothesis. Functional assays using Col2a1 conditional knockout (KO) mice are necessary to test whether Col2a1 deficiency affects BRONJ pathology. In histology (Fig. 6e, h), other cell types appear to express type II collagen more strongly than endothelial cells. The authors should clarify which cells these are, as their Col2a1 expression may be more relevant to the pathology. Generation of EC-specific Col2a1 KO mice would directly address this question.

Response: We thank the reviewer for the constructive comments and suggestions. We agree that functional validation is essential to confirm the bioinformatic predictions

regarding the *Col2a1–Cd44/Sdc4* axis and to clarify the pathogenic role of endothelial-derived Collagen II. Our specific responses are detailed below:

First, to evaluate whether suppressing the *Col2a1* axis rescues BRONJ pathology, we developed a localized delivery system by implanting *Col2a1*-targeting siRNA-loaded hydrogels directly into the extraction sockets of ZA-treated mice. This local intervention promoted soft tissue healing and restored osteogenesis within the extraction socket compared to the untreated BRONJ group (**Supplementary Fig. 15**). (**Page 11**)

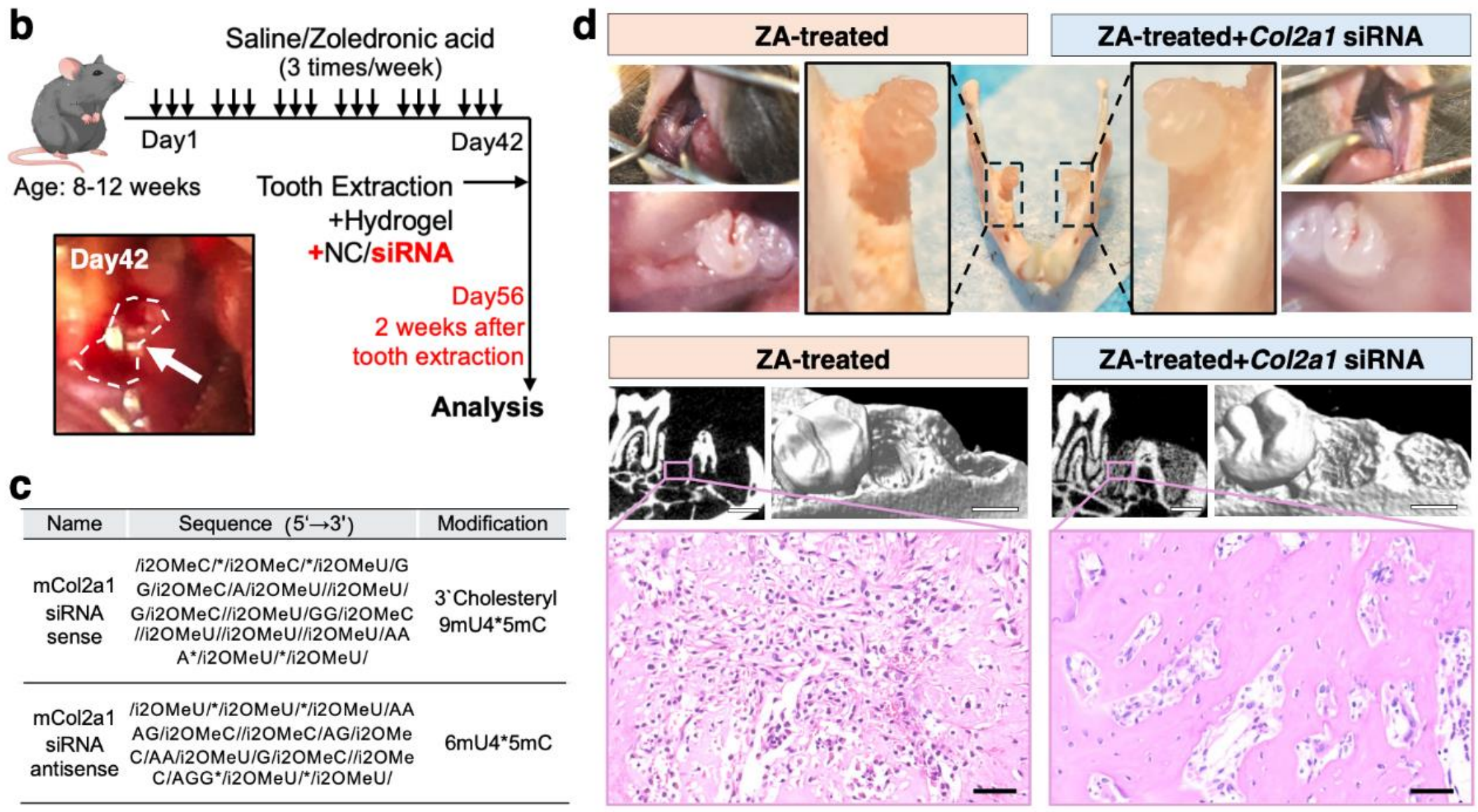

Name	Sequence (5'→3')	Modification
mCol2a1 siRNA sense	/i2OMeC/*/i2OMeC/*/i2OMeU/G G/i2OMeC/A/i2OMeU//i2OMeU/ G/i2OMeC//i2OMeU/GG/i2OMeC //i2OMeU//i2OMeU//i2OMeU/AA A*/i2OMeU/*/i2OMeU/	3`Cholesteryl 9mU4*5mC
mCol2a1 siRNA antisense	/i2OMeU/*/i2OMeU/*/i2OMeU/AA AG/i2OMeC//i2OMeC/AG/i2OMe C/AA/i2OMeU/G/i2OMeC//i2OMe C/AGG*/i2OMeU/*/i2OMeU/	6mU4*5mC

(Supplementary Fig. 15) **b** Schematic illustration of the local delivery of *Col2a1* siRNA-loaded hydrogels into the BRONJ extraction socket. **c** Sequences and modifications of the *Col2a1* siRNA sense and antisense strands. **d** Representative macroscopic images, micro-CT reconstructions, and H&E histological evaluations comparing the treated extraction sockets with the contralateral controls**. Scale bars:** white, 500 µm; black, 50 µm.

Further, to isolate the specific contribution of endothelial-derived Collagen II, we generated an endothelial-specific *Col2a1* conditional knockout (*Col2a1*$^{i\Delta EC}$) mouse model by crossing *Cdh5-Cre-Tg* (the Jackson Laboratory, no. 017968) and *Col2a1*$^{fl/fl}$ (Shanghai Southern Model Biotechnology Co., Ltd., NM-CKO-240983) mice. Notably, endothelial-specific deletion of *Col2a1* accelerated vascular recovery and enhanced tissue healing. These findings demonstrate that EC-derived Col2a1 upregulation is a primary pathogenic driver of vascular disruption in this model. (**Page 11**)

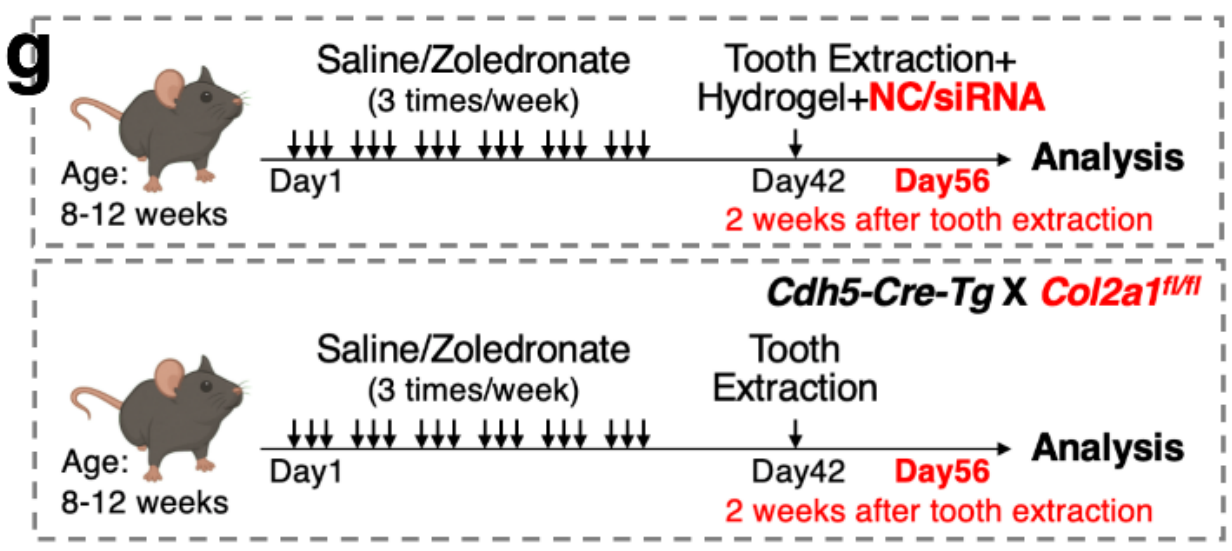

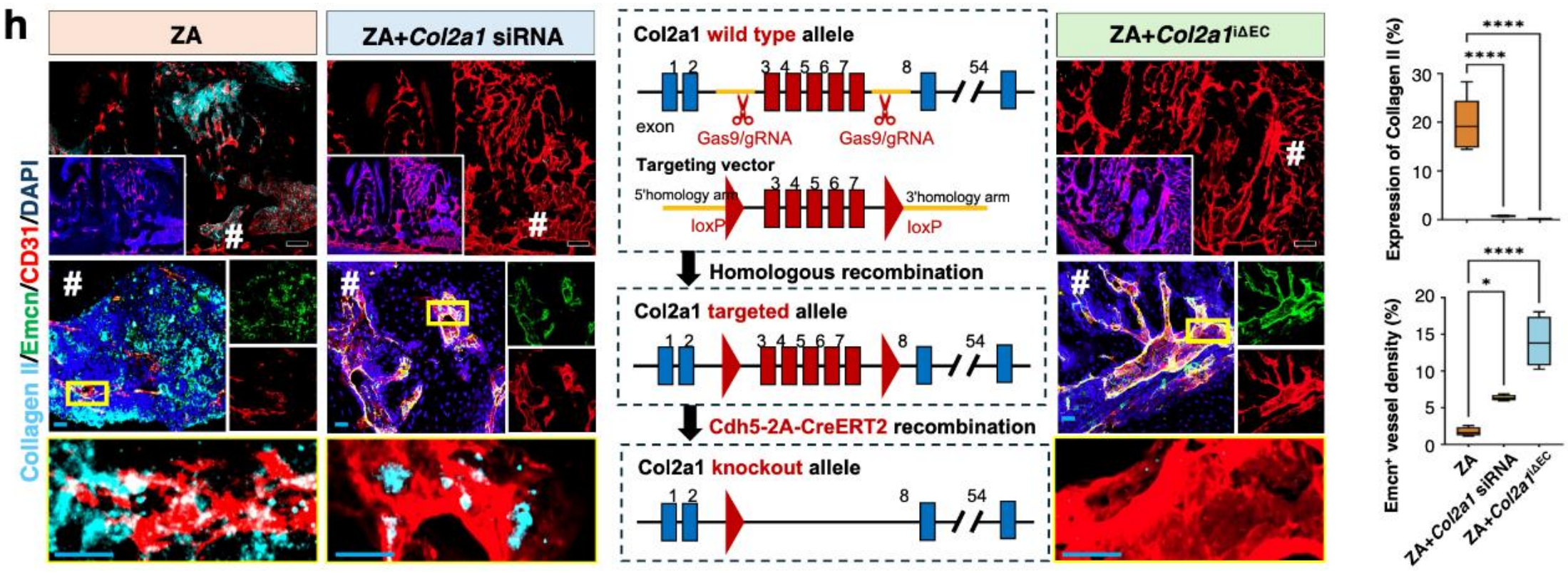

(Fig.6) g Experimental workflow: local delivery of NC or *Col2a1* siRNA via hydrogels immediately after tooth extraction in ZA-treated mice, and *Col2a1*$^{i\Delta EC}$ mice subjected to the same BRONJ induction. **h** Representative immunofluorescence images of the extraction socket, depicting the spatial distribution of blood vessels (*n=4*) and perivascular Collagen II deposition (*n=5*). Sections were immunostained for Emcn, CD31, and Collagen II, with nuclear counterstaining by DAPI. **Scale bars:** black, 50 µm; blue, 20µm. *P < 0.05, ****P< 0.0001.

Finally, a virtual genetic knockout using scTenifoldKnk on our BRONJ scRNA-seq dataset revealed that deleting endothelial *Col2a1* disrupts gene regulatory networks governing angiogenesis, inflammation, and osteogenesis (**Supplementary Fig. 16**). This bioinformatic perturbation complements our in vivo findings, confirming that endothelial *Col2a1* is centrally integrated into the vascular-osteogenic and inflammatory coupling mechanisms underlying BRONJ. (**Page 11**)

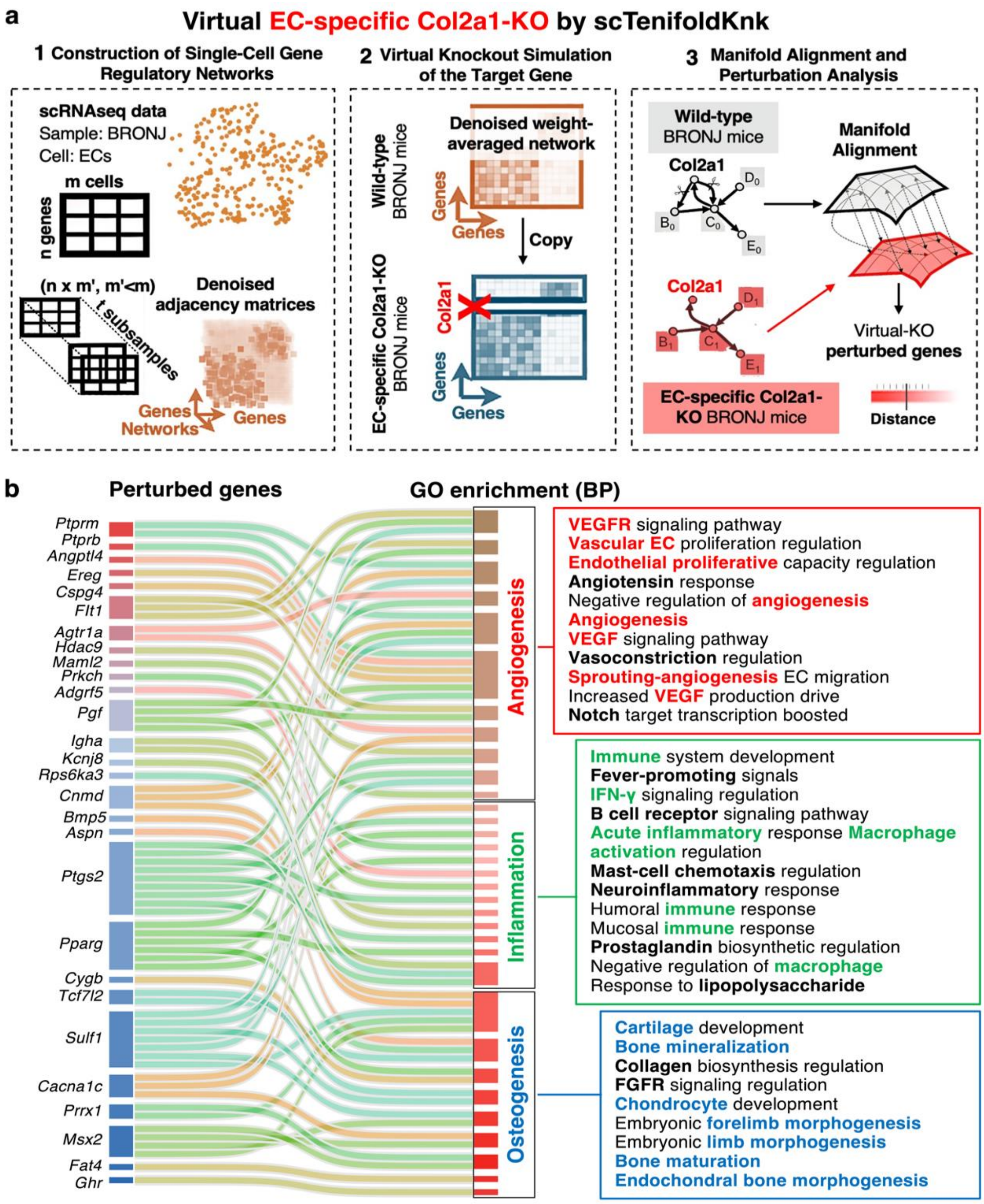

(Supplementary Fig. 16: Virtual endothelial *Col2a1* knockout via scTenifoldKnk.) a Schematic illustration of the computational workflow for the virtual gene knockout analysis. **b** GO enrichment analysis of the significantly perturbed gene network following *Col2a1* deletion, highlighting key biological processes associated with angiogenesis, inflammation, and osteogenesis.

***Q2*. It would strengthen the work to perform single-cell RNA-seq on human BRONJ samples (endothelial and stromal compartments) and compare these data with the mouse datasets.**

Response: We thank the reviewer for this constructive suggestion. We initially attempted scRNA-seq on human control and BRONJ mandibles; however, because these surgical specimens represent end-stage necrotic tissue, the isolated cells exhibited

extremely low viability and failed single-cell quality control (QC) thresholds. As a robust alternative, we performed bulk RNA-seq on 12 human samples (6 healthy controls vs. 6 BRONJ). Unfortunately, 3 BRONJ samples failed QC due to severe microbial contamination, a recognized clinical hallmark of exposed jaw necrosis (QC Figure). We therefore proceeded with sequencing and analysis of the remaining qualified samples.

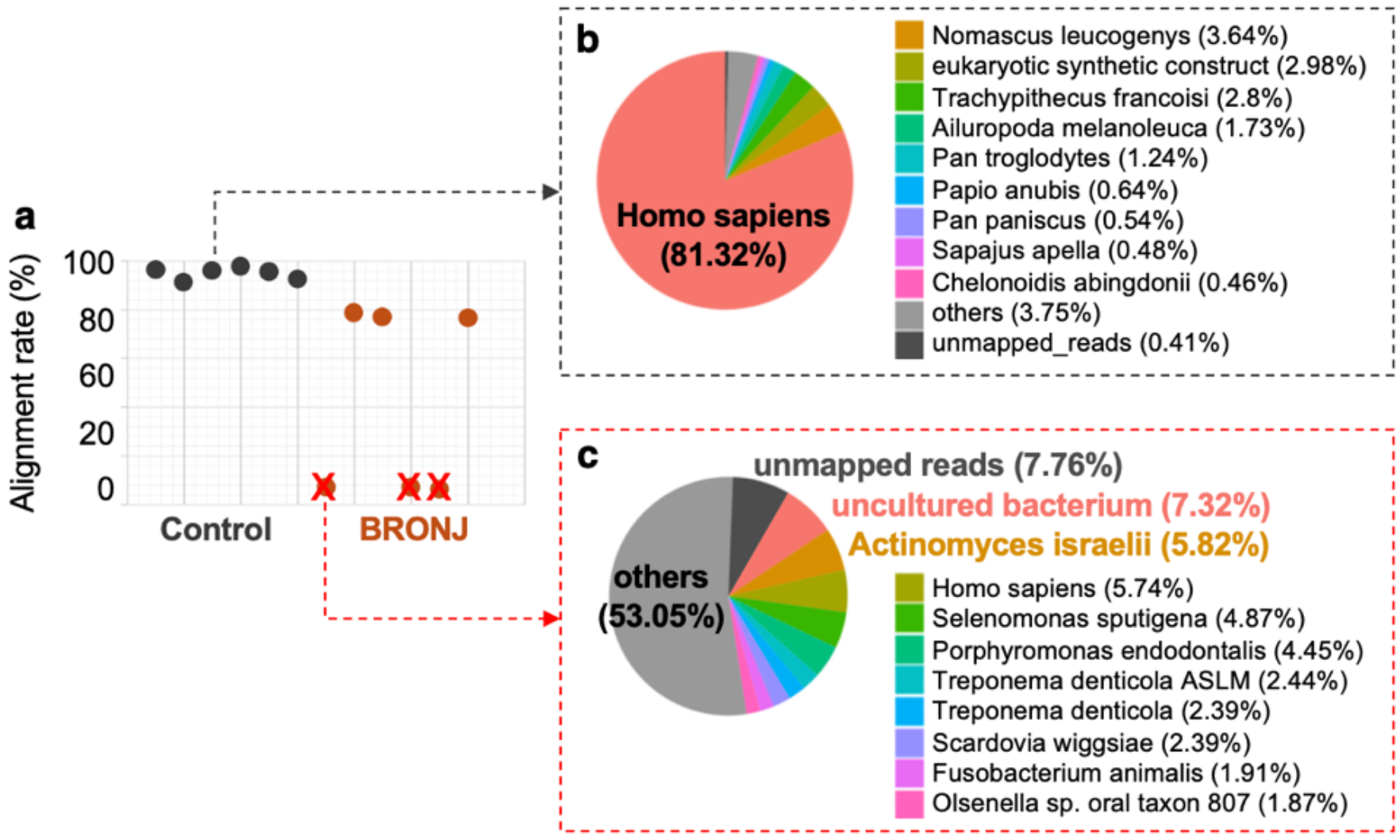

(QC Figure) a Alignment rates of the 6 control and 6 BRONJ human samples. **b** Contamination detection profile of a successfully sequenced sample passing QC. **c** Contamination detection profile of a failed sample, highlighting severe bacterial colonization.

Despite these constraints, cross-species transcriptomic comparison identified conserved pathogenic signatures between human and mouse datasets (**Supplementary Fig. 18a,b**). Conserved upregulated networks govern oxidative stress, matrix remodeling, and immune activation, whereas downregulated pathways are enriched in metabolism, vascular/lymphatic disruption, and osteogenesis under BRONJ condition (**Supplementary Fig. 18c**). These human profiles closely mimic our mouse findings, confirming that BRONJ involves vascular-stromal-immune dysregulation and endothelial decline. **(Page 12-13)**

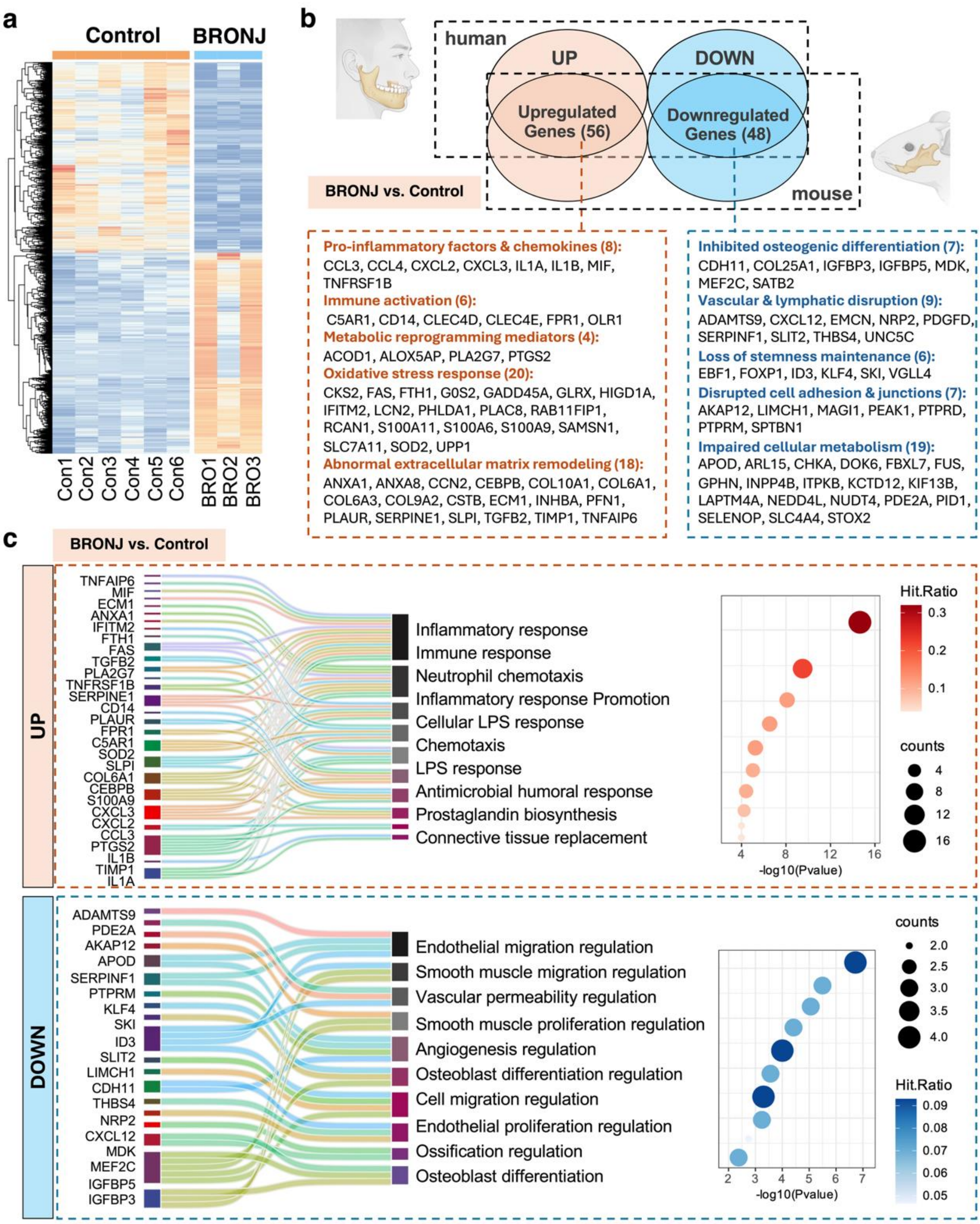

(Supplementary Fig. 18: Cross-species transcriptomic analysis of BRONJ.) a Heatmap illustrating the differentially expressed genes (DEGs) between human control and BRONJ samples. **b** Identification of conserved transcriptomic signatures, displaying the intersection of commonly upregulated and downregulated genes between the human and mouse transcriptomes. **c** GO functional enrichment analysis of the cross-species conserved DEGs.

Q3. The authors speculate that zoledronate-induced Col2a1 upregulation in ECs might influence fibroblasts or macrophages expressing SDC4 or CD44. This remains highly speculative without direct evidence. In vitro assays using endothelial (BECs and LECs) treated with zoledronate should be performed to test whether Col2a1 is indeed

upregulated in ECs. Moreover, EC remodeling may be a secondary effect driven by altered stromal activity and tissue remodeling. In CellChat, ECs were always defined as senders, which introduces bias. The authors should also analyze other cell types as senders to explore reciprocal interactions. Tools such as NicheNet could be also helpful for this.

Response: Thank you for this valuable suggestion. To provide direct in vitro evidence, we treated HUVECs (Procell, CL-0675) and hLECs (IMMOCELL, IM-H480) with zoledronic acid (ZA). Calcein-AM/PI staining revealed severe cellular damage and widespread death in both populations; hLECs lost their healthy cobblestone morphology, exhibiting cell retraction and rounding indicative of cytoskeletal collapse under ZA-treated condition. Crucially, RT-qPCR confirmed significant *Col2a1* mRNA upregulation in these treated cells (**Supplementary Fig. 14**). This cell-autonomous assay demonstrates that zoledronic acid directly induces endothelial *Col2a1* overexpression, independent of the microenvironment. (**Page 11**)

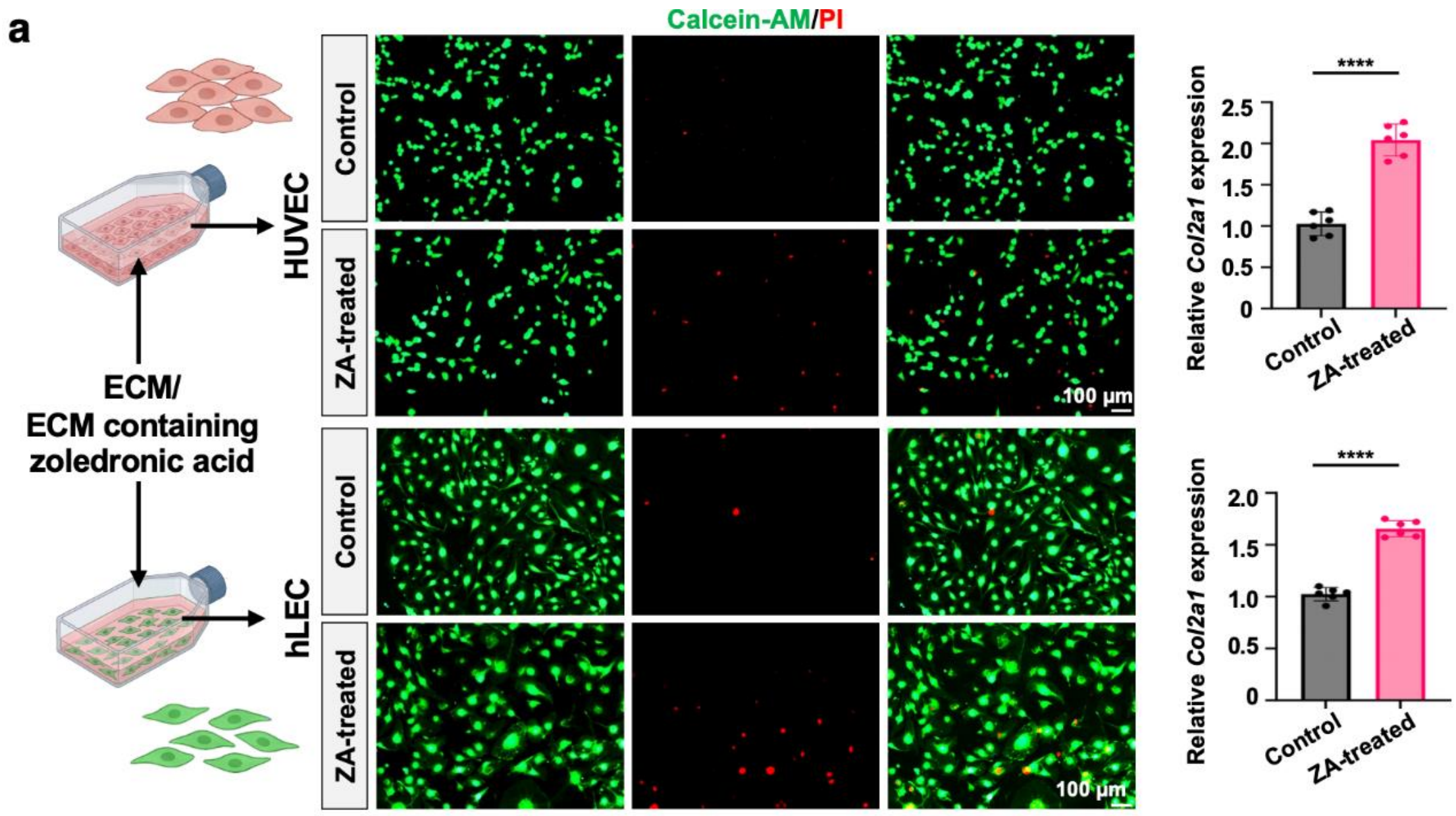

(Supplementary Fig. 14) a Representative Calcein-AM/PI staining and RT-qPCR evaluation of *Col2a1* mRNA expression in HUVECs and LECs cultured in endothelial cell complete medium (ECM) or ECM containing zoledronic acid (ZA). ****P < 0.0001; *n=6*.

Further, to assess if endothelial remodeling is secondary to stromal activity, NicheNet analysis (**Supplementary Fig. 7a**) was performed with endothelial cells as receivers and stromal cells as senders. Endothelial *Col2a1* expression received modest upstream regulation from the stroma, primarily via neutrophil-derived *Il1b* and fibroblast-derived *Bmp2*. Crucially, global signaling intensity from endothelial cells to other populations was substantially higher than the reciprocal stroma-to-endothelial signaling (**Supplementary Fig. 7b**). While stromal activity may contribute to a feedback loop, the relatively weak interaction strengths suggest that it plays a limited

role in driving pathology. **(Page 6)**

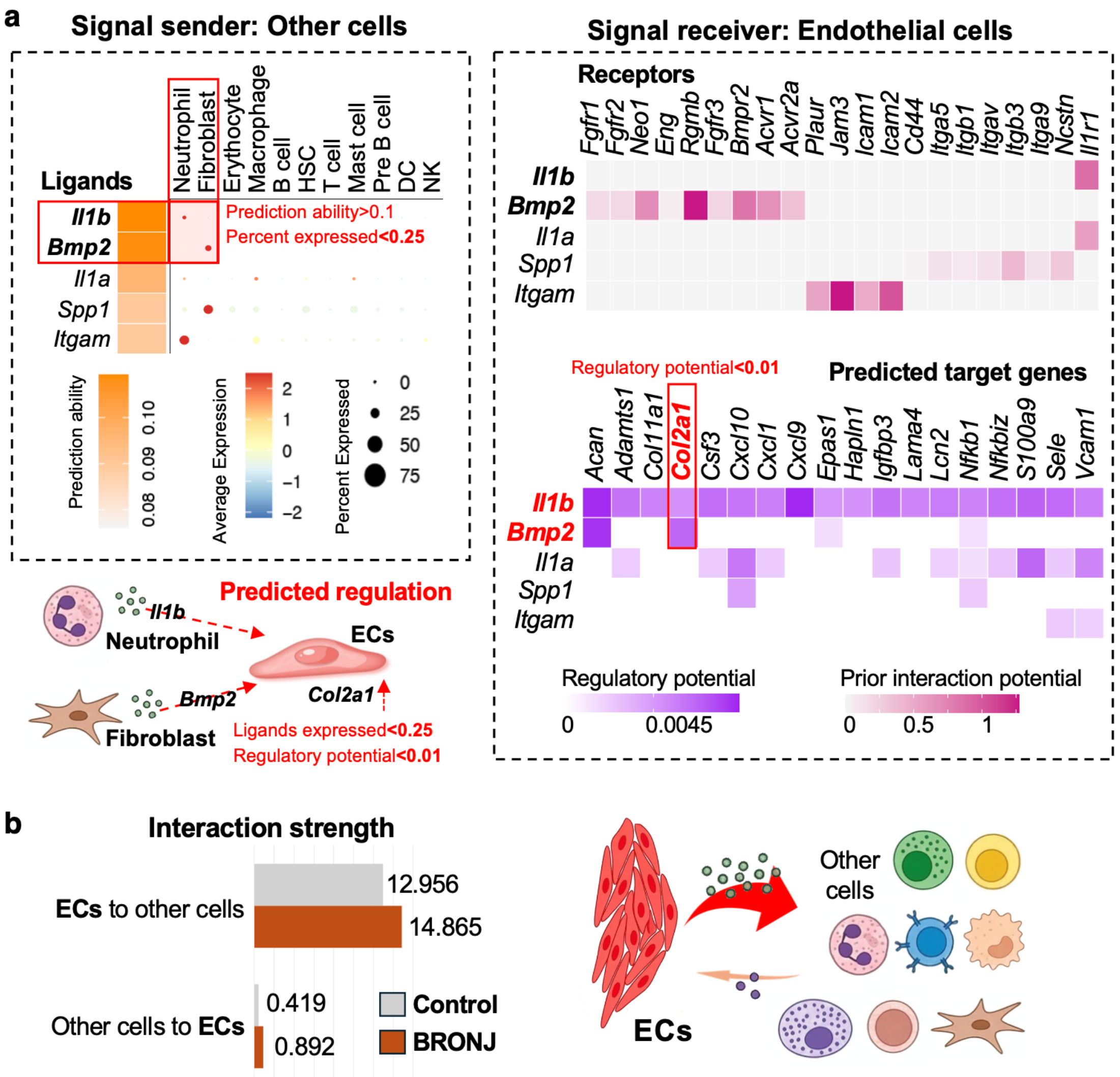

(Supplementary Fig. 7: Signaling dynamics and inflammatory assessment in the mandible.) a NicheNet analysis predicting key ligand-receptor interactions, designating other cell populations as signal senders and endothelial cells (ECs) as receivers, with EC differentially expressed genes serving as the predicted target genes. **b** Comparison of intercellular interaction strengths, accompanied by a schematic illustration detailing the differences in signaling intensities.

Q4. In line 97, the authors describe three vascular subtypes defined by Emcn and Podocalyxin. The manuscript should clearly show where these subtypes are located in the single-cell datasets.

Response: We thank the reviewer for the comment. To clarify the anatomical and phenotypic relationships, we mapped Emcn and Podocalyxin expression. AECs, representing arteries and arterioles, correspond to the Podocalyxin^{hi} Emcn^{lo} subtype, while VECs comprise both Podocalyxin^{hi} Emcn^{hi} and Podocalyxin^{lo} Emcn^{hi} subtypes. Spatially, the alveolar bone displays a distinct apical-to-coronal gradient characterized by decreasing Emcn and increasing Podocalyxin. Consequently, Podocalyxin^{lo} Emcn^{hi} VECs localize at the alveolar-mandibular junction, Podocalyxin^{hi} Emcn^{hi} VECs

dominate the apical region, and the Podocalyxin^{hi} Emcn^{lo} arterial network restricts primarily to coronal, gingival-facing aspects. (**Supplementary Fig. 8**) (**Page 6**)

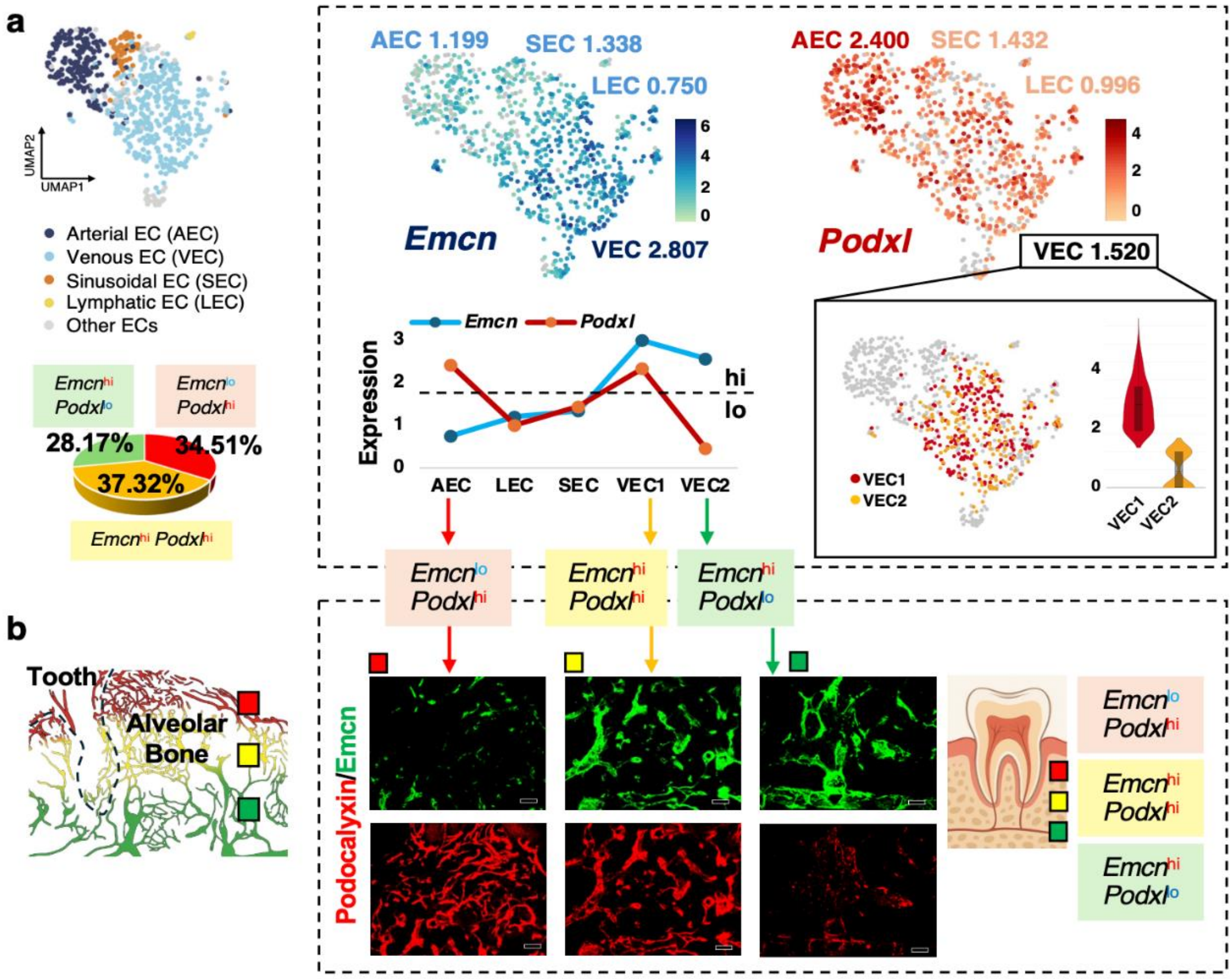

(**Supplementary Fig. 8**) **a** scRNA-seq analysis detailing the expression profiles, relative abundance, and cluster distribution of Emcn and Podxl across endothelial cell subpopulations. **b** Representative immunofluorescence images illustrating the spatial localization of Emcn^{hi} and Podocalyxin^{hi} vascular networks within the mandible.

Q5. Lymphatic structures in the tooth–jawbone region have not, to my knowledge, been previously characterized. It would therefore be valuable to visualize lymphatics in the cleared tissue to determine their spatial distribution. Additional LEC markers such as PROX1 or PDPN should be used, since LYVE1 is not specific for lymphatic endothelium, as the authors themselves note.

Response: Thank you for this important suggestion. Based on its high enrichment in mandibular lymphatics, we used FLT4 (VEGFR3) as a marker and performed FLT4/Emcn co-staining to visualize these local lymphatic structures. (**Page 6**)

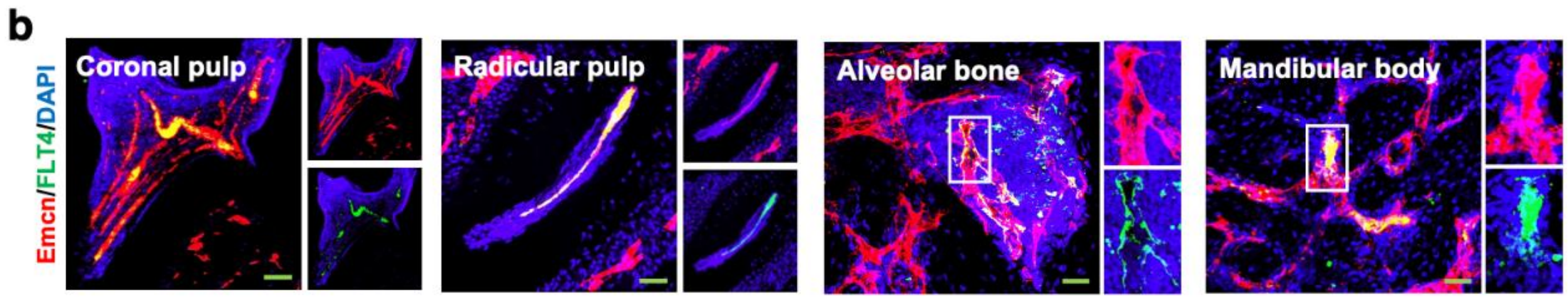

(Supplementary Fig. 9) b Lymphatic vessels in mandible immunostained for FLT4 and Emcn, with nuclear counterstaining by DAPI. **Scale bars:** yellow, 1000 μm; green, 100 μm.

Furthermore, we have integrated public multi-species scRNA-seq datasets (beagle dog, mouse, and human) and identified mandibular LECs that closely align with our findings **(Supplementary Fig. 10)**. Establishing a comprehensive molecular atlas of LECs remains an important goal for future studies. **(Page 6)**

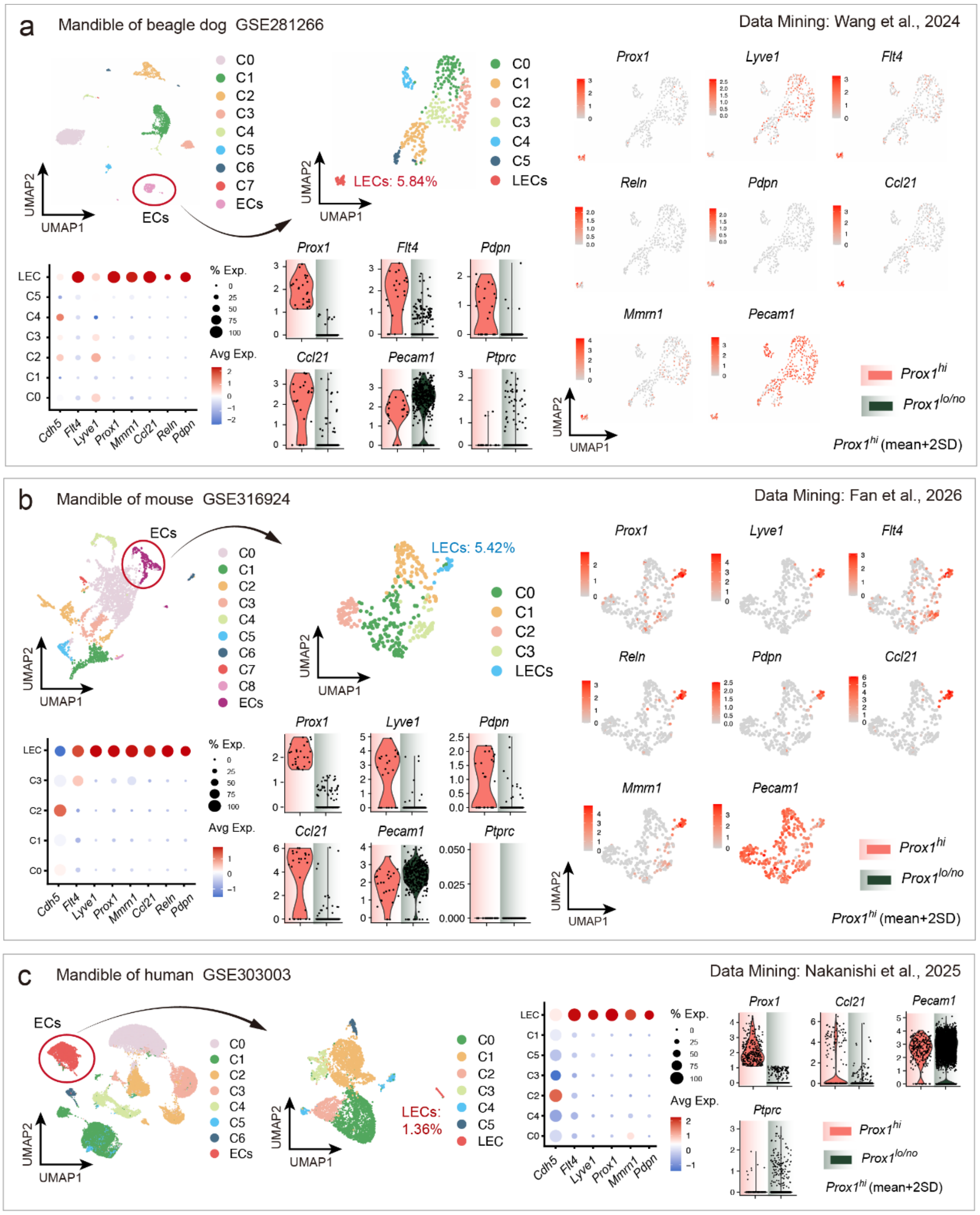

(Supplementary Fig. 10) a Analysis of LEC in the mandible of beagle dogs derived from the GSE281266 dataset. **b** Analysis of LEC in the mouse mandible derived from the GSE316924 dataset. **c** Comparative analysis of mandibular LEC using the human dataset GSE303003.

***Q6*. The authors suggest that BRONJ induces pro-inflammatory responses in ECs and macrophages. However, Fig. 2c shows minimal immune infiltration, which would suggest a non-inflammatory state. The authors need to explain this discrepancy and reconcile histological findings with transcriptional data.**

Response: Thank you for highlighting this discrepancy. In our initial unsorted single-cell sequencing of post-extraction tissues, immune cells exceeded 98%, masking endothelial and stromal populations. To overcome this, we employed magnetic-activated cell sorting (MACS) to enrich the vascular-stromal compartments. Following established bone marrow niche mapping protocols (Bandyopadhyay et al., *Cell*, 2024, Fig. 1A), this targeted immune depletion strategy effectively reduced immune cell overrepresentation and enabled more comprehensive downstream analyses.

[FIGURE REDACTED]

Single-cell sorting strategy and baseline immune infiltration. a Schematic representation of the magnetic bead-based single-cell RNA sequencing sorting strategy utilized to enrich rare stromal/endothelial populations, adapted from Bandyopadhyay et al. (*Cell*, 2024). **b** Unsorted single-cell RNA sequencing results from control and BRONJ mandibles, demonstrating the profound baseline immune infiltration (>98% of total cells) prior to magnetic sorting.

Taken from references: Bandyopadhyay S, Duffy MP, Ahn KJ, et al. Mapping the cellular biogeography of human bone marrow niches using single-cell transcriptomics and proteomic imaging. *Cell*. 2024 Jun 6;187(12):3120-3140.e29. doi: 10.1016/j.cell.2024.04.013.

We then evaluated the local microenvironment with histological analysis, which remained highly pro-inflammatory. Consistent with our transcriptomic data showing upregulation of the "cellular response to IL-1" pathway, immunohistochemical staining confirmed that IL-1β expression is significantly elevated throughout BRONJ tissues

compared to healthy controls (**Supplementary Fig. 6b**). **(Page 6)**

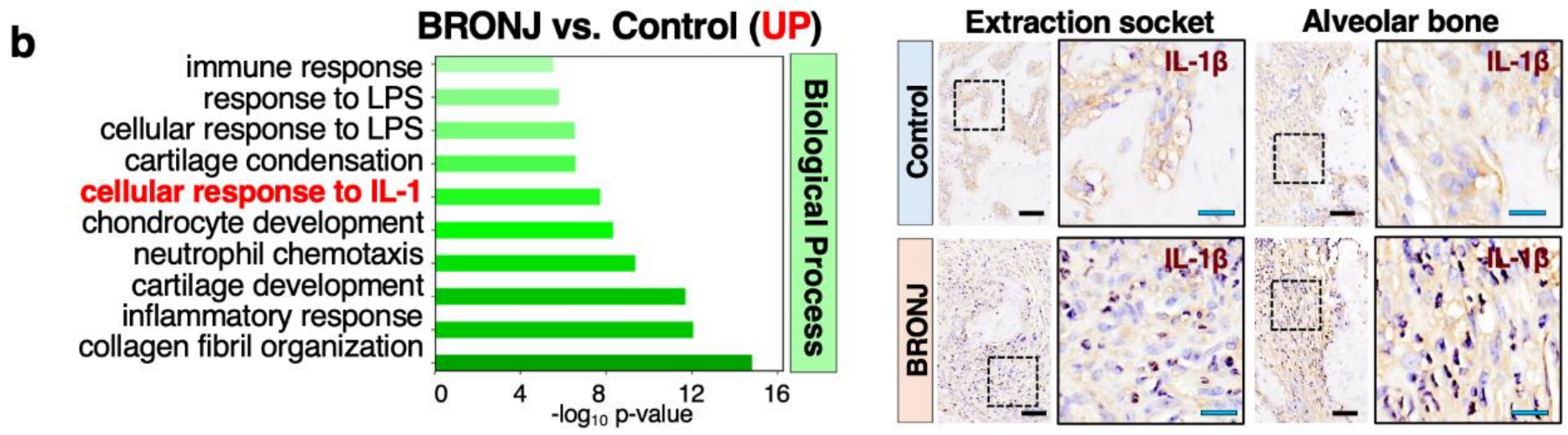

(Supplementary Fig. 6) b GO functional enrichment analysis of the upregulated genes in the BRONJ group compared to the control. Representative immunohistochemical staining images of IL-1β in the extraction socket and alveolar bone. **Scale bars:** black, 50 μm; blue, 20 μm.

Q7. In the endothelial single-cell dataset, capillary ECs seem to be absent. Do the authors believe this tissue lacks capillaries, were these cells lost during processing or filtering or annotated wrongly? Clarification is required.

Response: Thank you for your comment. Capillary endothelial cells are well preserved in our dataset. Given our focus on specialized bone vasculature, this population was initially annotated using tissue-specific nomenclature as sinusoidal endothelial cells (SECs). Based on established endothelial atlases (Kalucka et al., *Cell*; Bondareva et al., *Nat. Metab.*), we confirmed that our SEC cluster was enriched for canonical capillary markers, including *Car4*, *Aqp7*, *Tcf15*, and *Cd36*.

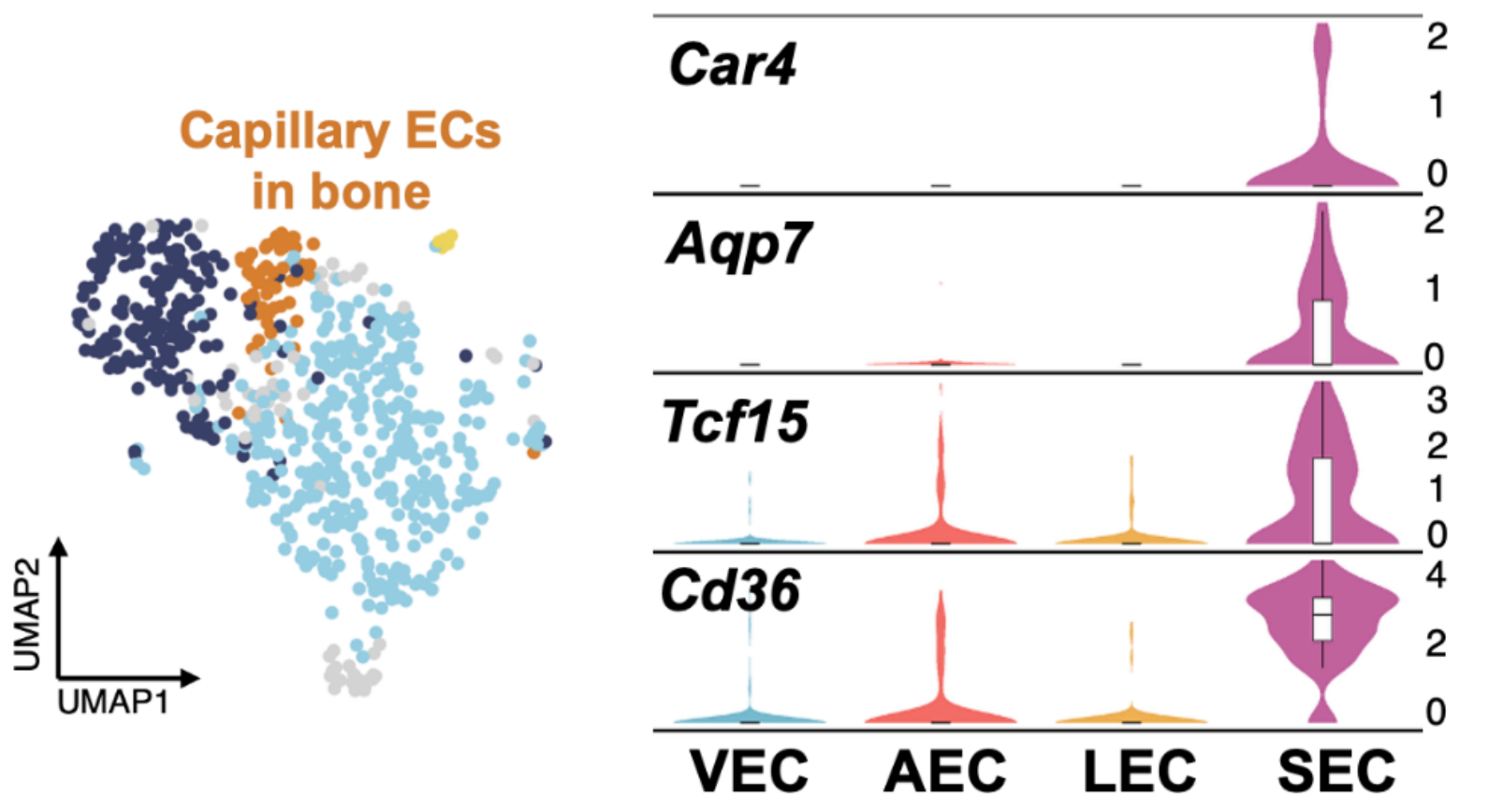

Expression of canonical capillary endothelial markers, including *Car4*, *Aqp7*, *Tcf15*, and *Cd36*, localized within the annotated SEC cluster.

References: 1) Kalucka J, et al. Single-Cell Transcriptome Atlas of Murine Endothelial Cells. *Cell*. 2020 Feb 20;180(4):764-779.e20. doi: 10.1016/j.cell.2020.01.015.; 2) Bondareva O, et al. Single-cell profiling of vascular endothelial cells reveals progressive organ-specific vulnerabilities during obesity. *Nat Metab*. 2022 Nov;4(11):1591-1610. doi: 10.1038/s42255-022-00674-x.

Q8. In Fig. 5B, the dataset includes a population labeled as "progenitor macrophages." The authors should clarify what these cells represent. Are they monocytes or

proliferative macrophages? This point needs explanation, as the presence of progenitor macrophages in this tissue is unexpected.

Response: We thank the reviewer for this point. Re-evaluation of the transcriptomic profile for this subpopulation showed strong GO enrichment for cell division and the mitotic cycle, confirming a locally dividing state rather than undifferentiated precursors. Accordingly, we have updated the annotation from "progenitor" to "proliferating macrophages" across the revised text and figures to ensure biological precision. (**Page 8**)

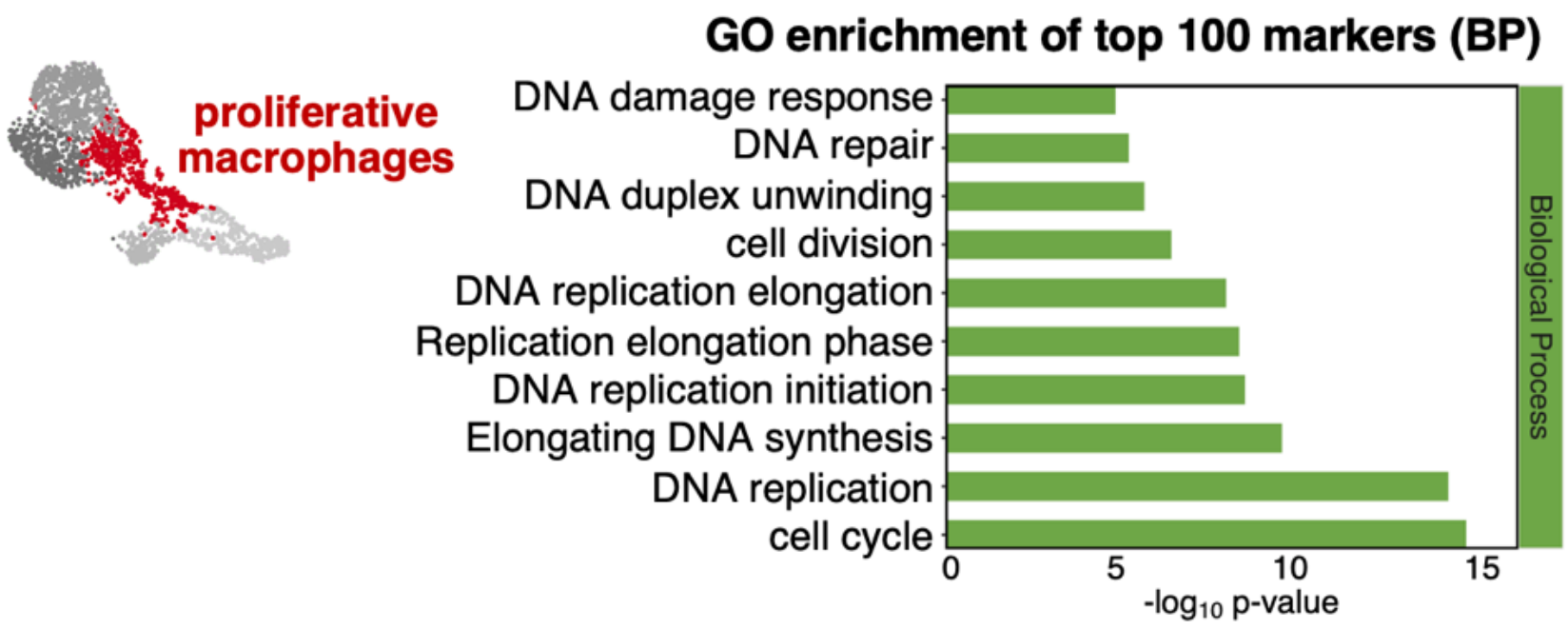

GO functional enrichment analysis of biological processes based on the top 100 expressed marker genes defining the proliferative macrophage cluster

Response to Reviewer 2:

Based on a novel imaging approach of the intact tooth–jaw structure and in-depth scRNA-seq analyses, the authors identified four vascular subtypes, eight fibroblast subtypes, and two macrophage subtypes. In BRONJ, there was a significant depletion of all vascular subtypes except sinusoidal endothelial cells (SECs), whereas LRRC15$^+$ and CHAD$^+$ fibroblasts and IL6$^+$ macrophages selectively expanded. Furthermore, the COL2A1–CD44/SDC4 axis was highlighted as a BRONJ-specific endothelial-to-fibroblast/macrophage signaling pathway. Overall, this was a compelling study that delineated vascular and lymphatic dysregulation via non-EndoMT mechanisms in BRONJ. Nevertheless, the work remains incomplete without functional evidence. To substantiate the proposed role of the COL2A1–CD44/SDC4 axis, targeted genetic or pharmacologic perturbations in appropriate BRONJ models are required.

Response: We thank the reviewer for noting that targeted genetic or pharmacological perturbations in an appropriate BRONJ model are required to validate the role of the *Col2a1–Cd44/Sdc4* axis. Following this suggestion, we have performed a series of in vivo functional assays and in silico validations, as detailed below:

First, to evaluate pharmacologic perturbations in BRONJ models, we developed a localized delivery system by implanting *Col2a1*-targeting siRNA-loaded hydrogels directly into the extraction sockets of ZA-treated mice. This local intervention significantly promoted soft tissue healing and restored osteogenesis within the

extraction socket compared to the untreated BRONJ group (**Supplementary Fig. 15**). **(Page 11)**

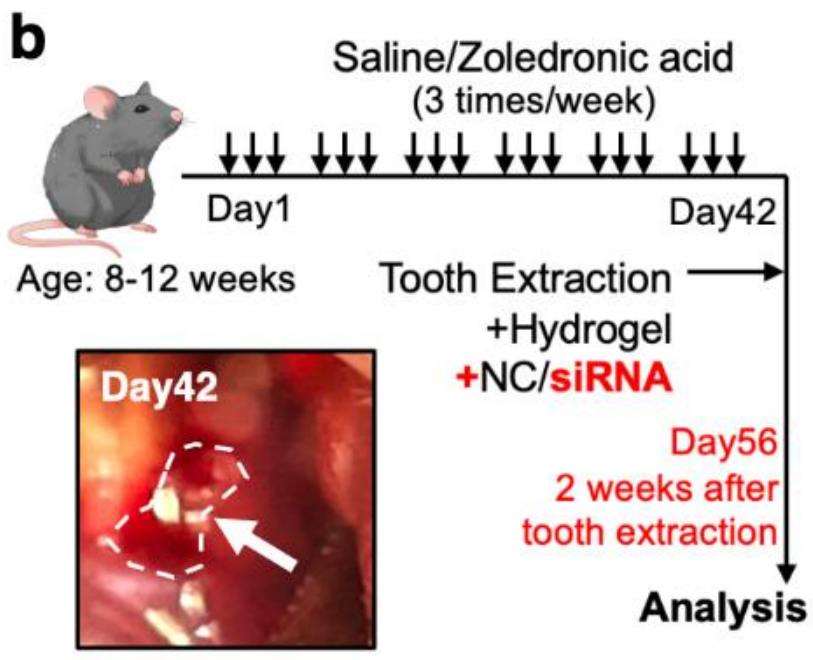

c

Name	Sequence (5'→3')	Modification
mCol2a1 siRNA sense	/i2OMeC/*/i2OMeC/*/i2OMeU/GG/i2OMeC/A/i2OMeU//i2OMeU/G/i2OMeC//i2OMeU/GG/i2OMeC//i2OMeU//i2OMeU//i2OMeU/AAA*/i2OMeU/*/i2OMeU/	3`Cholesteryl 9mU4*5mC
mCol2a1 siRNA antisense	/i2OMeU/*/i2OMeU/*/i2OMeU/AAAG/i2OMeC//i2OMeC/AG/i2OMeC/AA/i2OMeU/G/i2OMeC//i2OMeC/AGG*/i2OMeU/*/i2OMeU/	6mU4*5mC

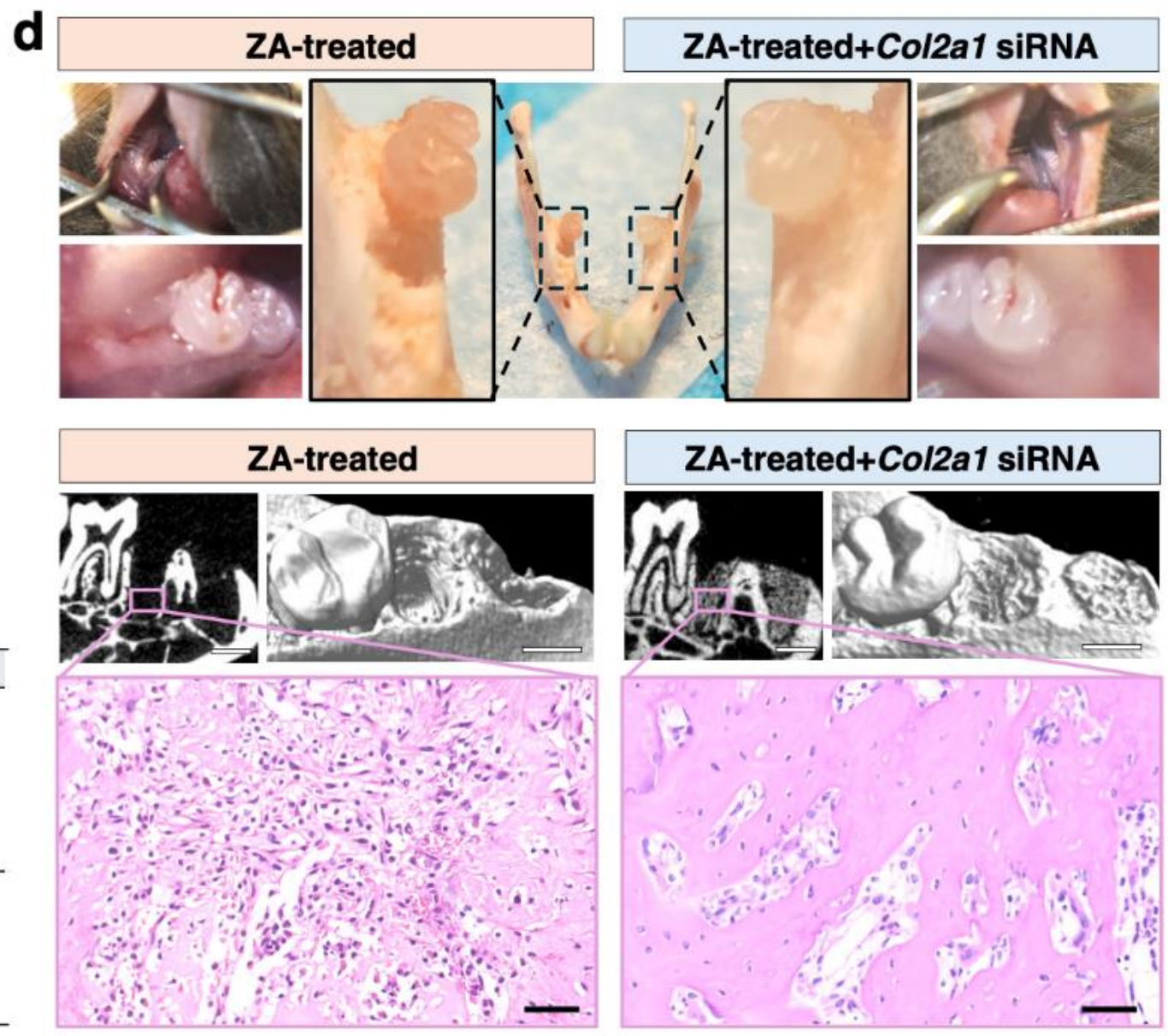

(Supplementary Fig. 15) **b** Schematic illustration of the local delivery of *Col2a1* siRNA-loaded hydrogels into the BRONJ extraction socket. **c** Sequences and modifications of the *Col2a1* siRNA sense and antisense strands. **d** Representative macroscopic images, micro-CT reconstructions, and H&E histological evaluations comparing the treated extraction sockets with the contralateral controls**. Scale bars:** white, 500 µm; black, 50 µm.

Further, complementing the pharmacological siRNA intervention, we implemented a targeted genetic strategy by crossing *Cdh5-Cre-Tg* (the Jackson Laboratory, no. 017968) and *Col2a1*$^{fl/fl}$ (Shanghai Southern Model Biotechnology Co., Ltd., NM-CKO-240983) mice, to generate the *Col2a1*$^{i\Delta EC}$ mouse model. Notably, endothelial-specific deletion of *Col2a1* accelerated vascular recovery and enhanced tissue healing. These findings demonstrate that endothelial-derived *Col2a1* upregulation is a primary pathogenic driver of vascular disruption in this model. **(Page 11)**

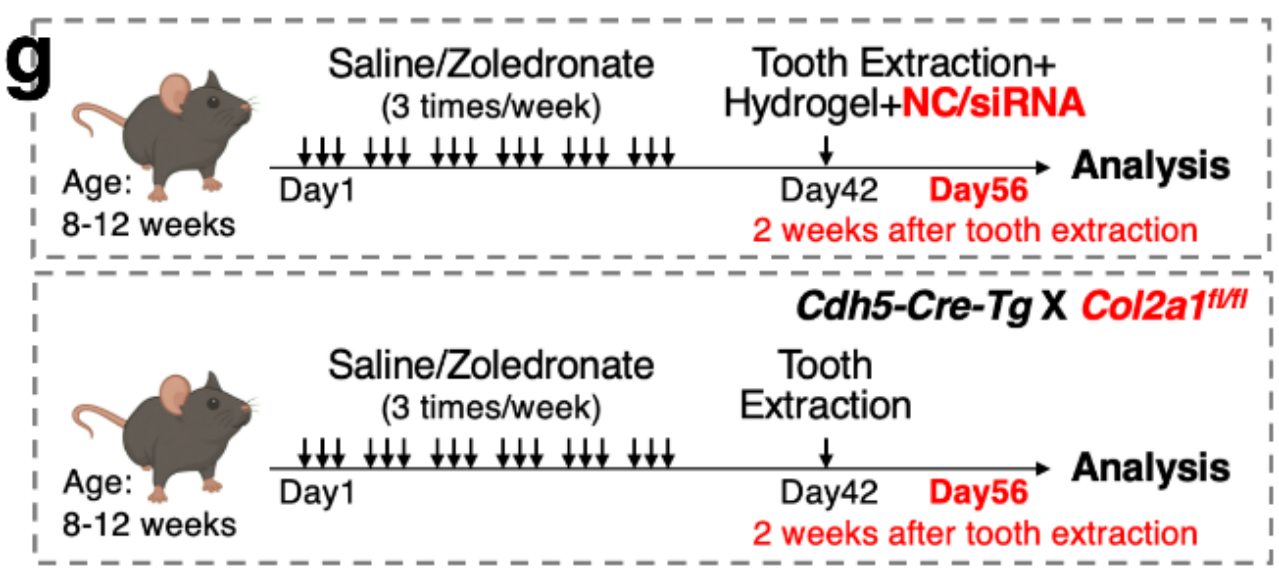

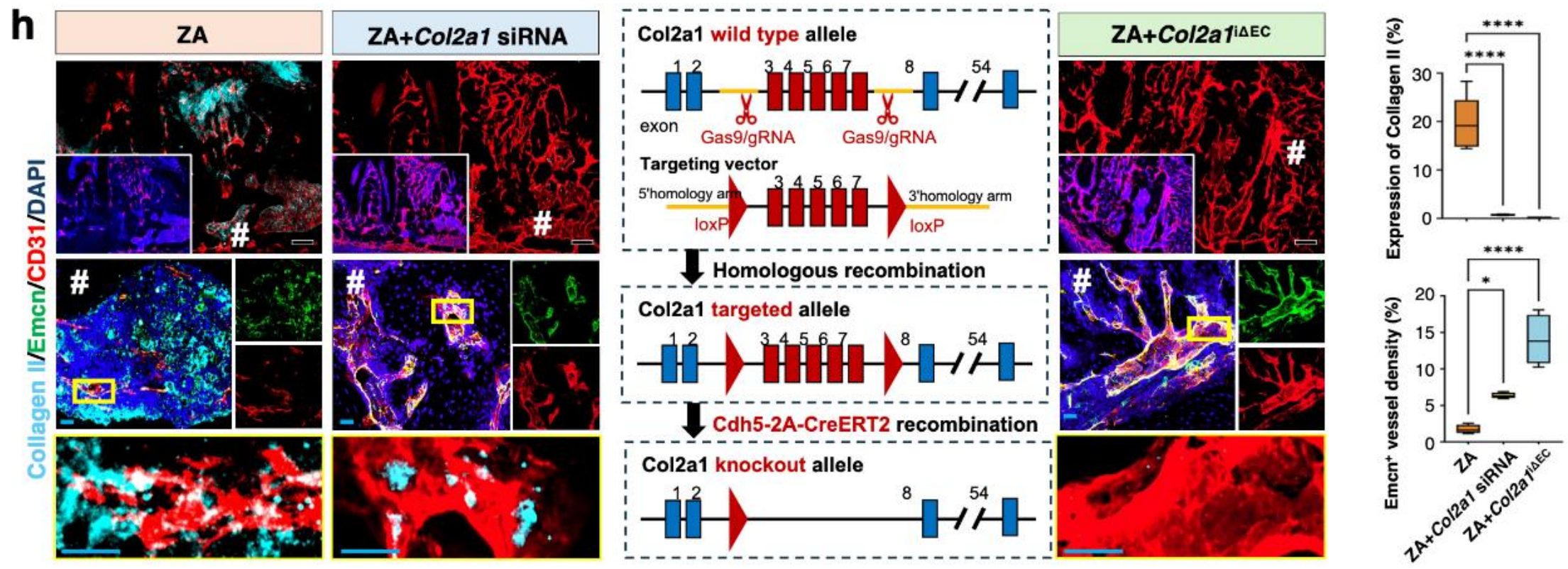

(Fig.6) g Experimental workflow: local delivery of NC or *Col2a1* siRNA via hydrogels immediately after tooth extraction in ZA-treated mice, and *Col2a1*^{iΔEC} mice subjected to the same BRONJ induction. **h** Representative immunofluorescence images of the extraction socket, depicting the spatial distribution of blood vessels (*n=4*) and perivascular Collagen II deposition (*n=5*). Sections were immunostained for Emcn, CD31, and Collagen II, with nuclear counterstaining by DAPI. **Scale bars:** black, 50 µm; blue, 20µm. *P < 0.05, ****P< 0.0001.

Then, a virtual genetic knockout using scTenifoldKnk on our BRONJ scRNA-seq dataset revealed that deleting endothelial *Col2a1* disrupts gene regulatory networks governing angiogenesis, inflammation, and osteogenesis (**Supplementary Fig. 16**). This bioinformatic perturbation complements our in vivo findings, confirming that endothelial *Col2a1* is centrally integrated into the vascular-osteogenic and inflammatory coupling mechanisms underlying BRONJ. **(Page 11)**

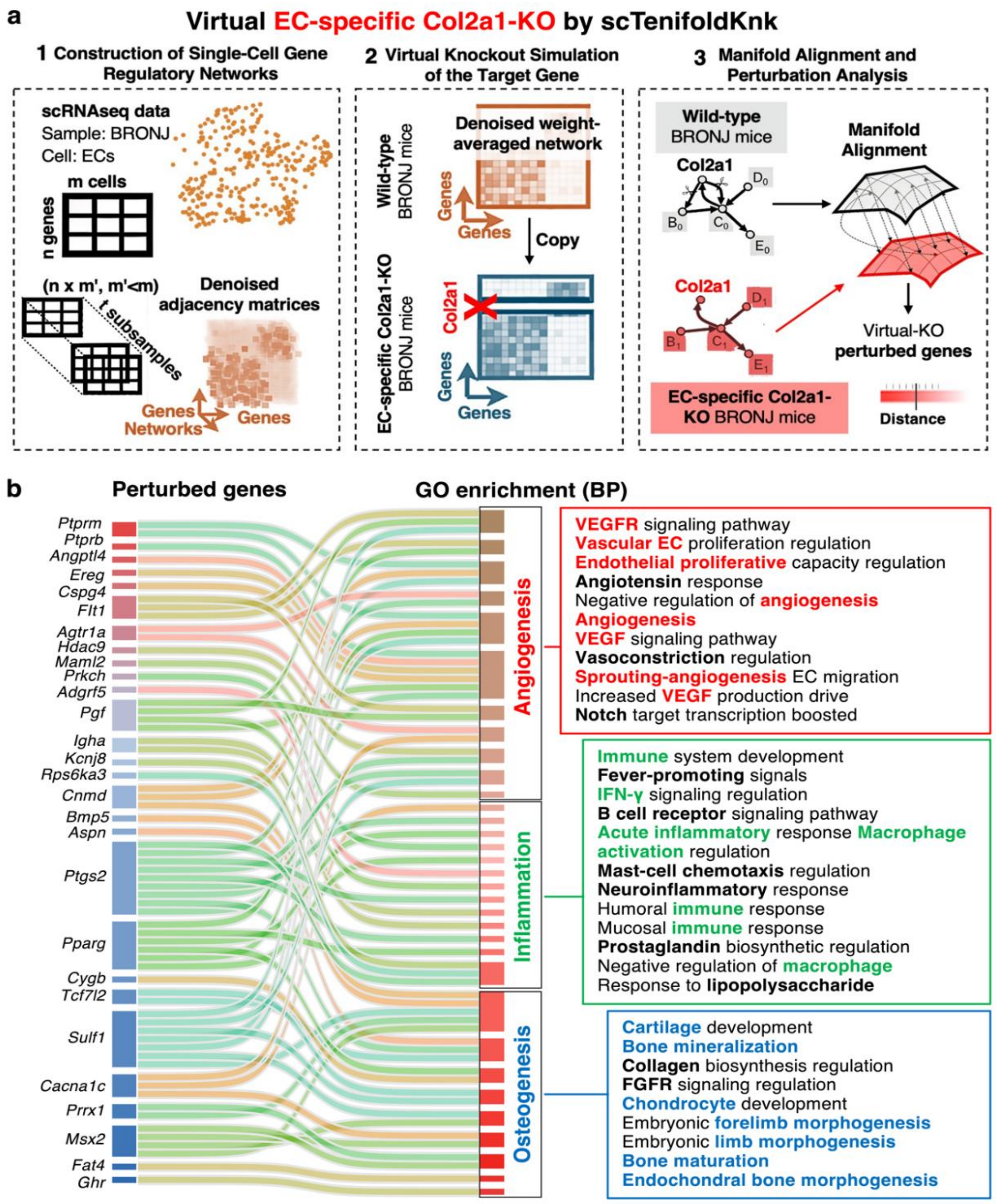

(Supplementary Fig. 16: Virtual endothelial *Col2a1* knockout via scTenifoldKnk.) a Schematic illustration of the computational workflow for the virtual gene knockout analysis. **b** GO enrichment analysis of the significantly perturbed gene network following *Col2a1* deletion, highlighting key biological processes associated with angiogenesis, inflammation, and osteogenesis.

Q1. In Figure 1d, the authors identified three vascular subtypes, Emcn-low/Podcalyxin-low, Emcn-high/Podcalyxin-high, and Emcn-high/Podcalyxin-low, in the mandibular alveolar bone. However, the relative abundance of these subtypes remains unclear. Furthermore, the relationship between these Emcn/Podocalyxin-defined subtypes and the four vascular subtypes described in Figure 3a should be clarified. In addition, since Podcalyxin-expressing endothelial cells in long-bone marrow have not been clearly characterized, it would be helpful to clarify the similarities and differences in vascular

subtypes between long bone and mandibular alveolar bone.

Response: We thank the reviewer for the comment. We have quantified the relative abundance of each subpopulation and mapped their correspondence. Specifically, AECs, representing arteries and arterioles, correspond to the Podocalyxin^{hi} Emcn^{lo} subtype, while VECs comprise both the Podocalyxin^{hi} Emcn^{hi} and Podocalyxin^{lo} Emcn^{hi} subtypes. Spatially, the alveolar bone displays a distinct apical-to-coronal gradient characterized by decreasing Emcn and increasing Podocalyxin. Consequently, Podocalyxin^{lo} Emcn^{hi} VECs localize at the alveolar-mandibular junction, Podocalyxin^{hi} Emcn^{hi} VECs dominate the apical region, and the Podocalyxin^{hi} Emcn^{lo} arterial network restricts primarily to coronal, gingival-facing aspects. **(Page 6)**

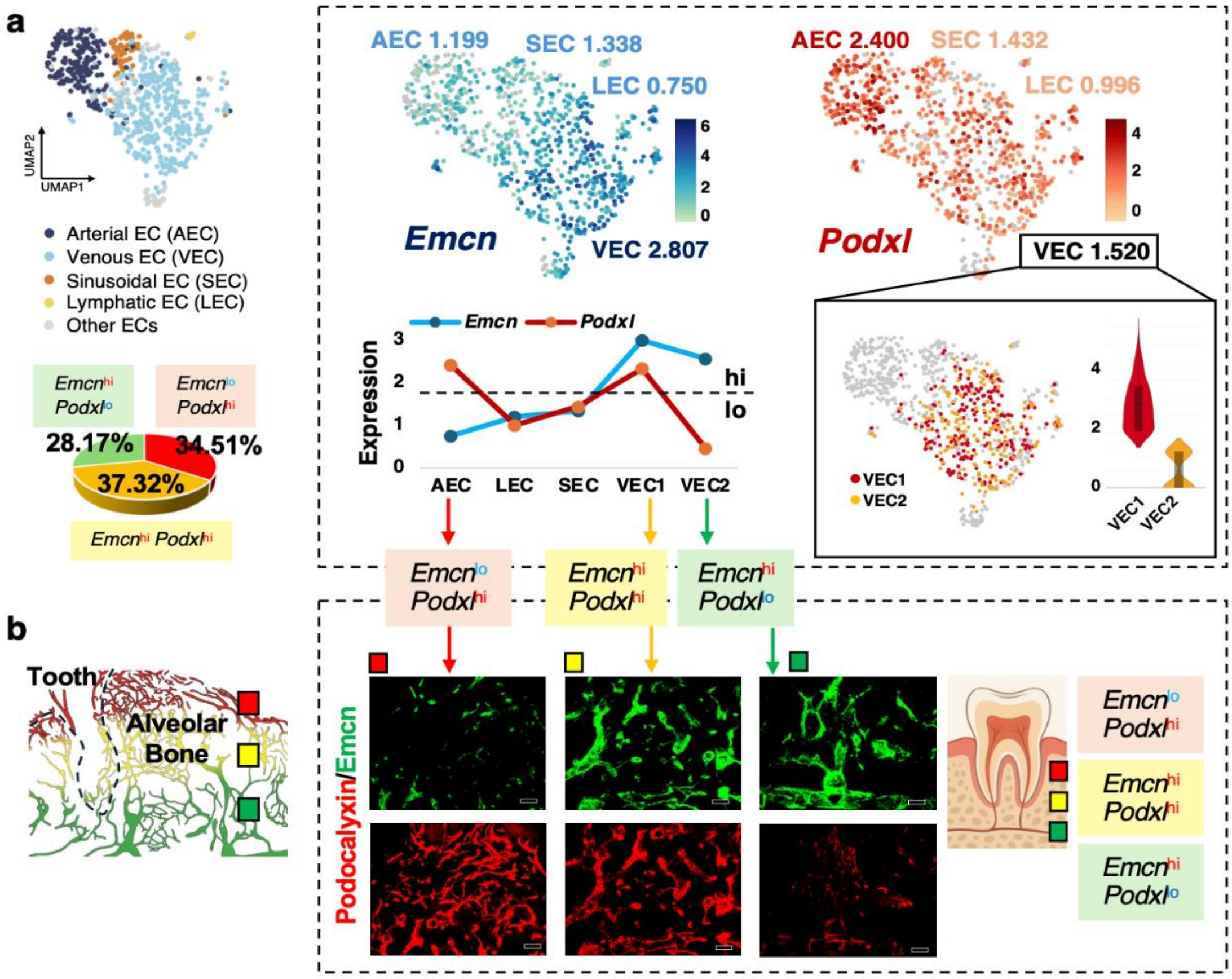

(Supplementary Fig. 8) a scRNA-seq analysis detailing the expression profiles, relative abundance, and cluster distribution of Emcn and Podxl across endothelial cell subpopulations. **b** Representative immunofluorescence images illustrating the spatial localization of Emcn^{hi} and Podocalyxin^{hi} vascular networks within the mandible.

As suggested, we performed additional immunostaining of long bones. Preliminary analysis of the femur suggests that Podocalyxin^{+} vascular networks are also present in the metaphyseal region adjacent to the growth plate. While these findings suggest regional differences between long bone and mandibular vascular beds, definitive anatomical mapping will require further investigation.

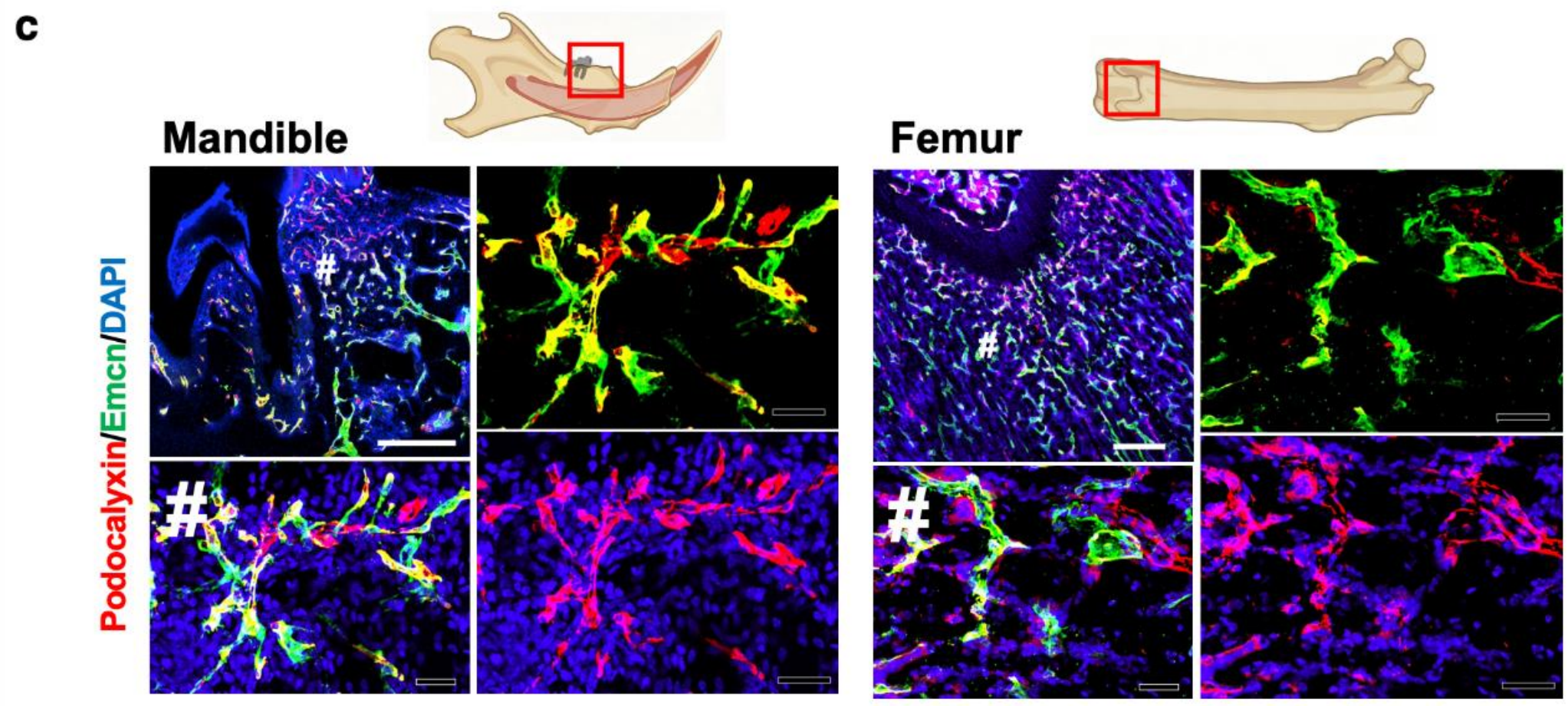

(Supplementary Fig. 8) c Comparative immunofluorescence evaluation of Emcn and Podxl expression in the vascular beds of the mandible and femur, with nuclei counterstained with DAPI. **Scale bars:** white, 500 µm; black, 50 µm.

<u>*Q2*. In Figure 1g and 1h, the authors showed a reduction of type H vessels, arteries, and lymphatic vessels in the BRONJ group. However, the spatial relationship between the decreased vessels and the necrotic region remains unclear.</u>

Response: Thank you for the comment. To address the spatial relationship between vascular decline and bone necrosis, we have included a schematic illustration along with bounding boxes in Figures 1g and 1h to demarcate the immunofluorescence regions. These imaging enhancements confirm that areas of jaw necrosis spatially coincide with marked depletion of local vascular and lymphatic networks.

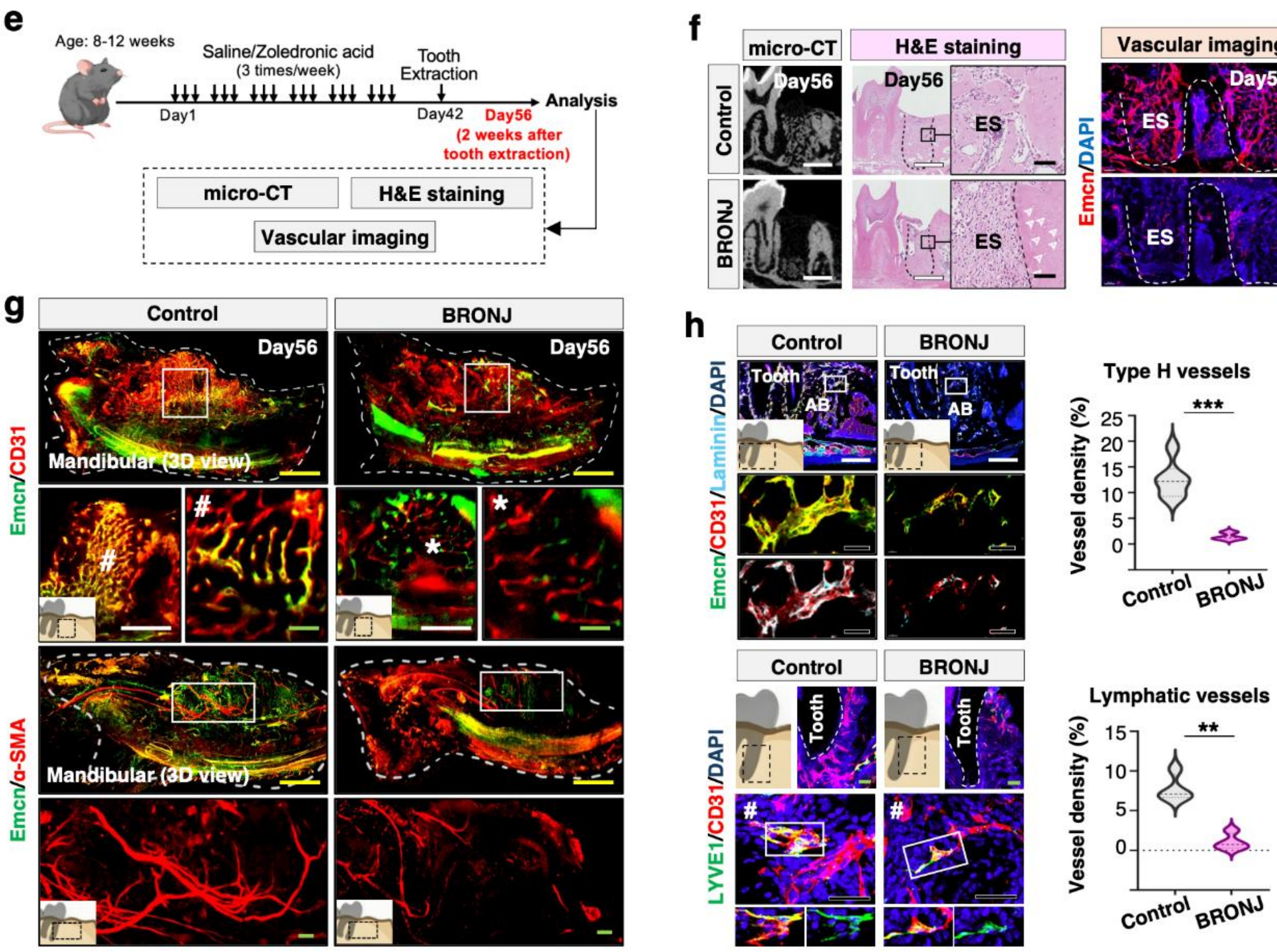

Q3. In Figure 2, the expression of Sele was decreased in BRONJ. The underlying mechanism remains unclear.

Response: We thank the reviewer for raising this important mechanistic point. SCENIC analysis and protein-protein interaction (PPI) networks revealed that *Sele* (*Esel*) is a potential downstream target of *Nfkb1*, identifying an NF-κB/E-selectin axis where *Nfkb*1 activation drives *Sele* (*Esel*) transcription to mediate leukocyte tethering and rolling. We will further validate this mechanism in future studies. (**Page 6**)

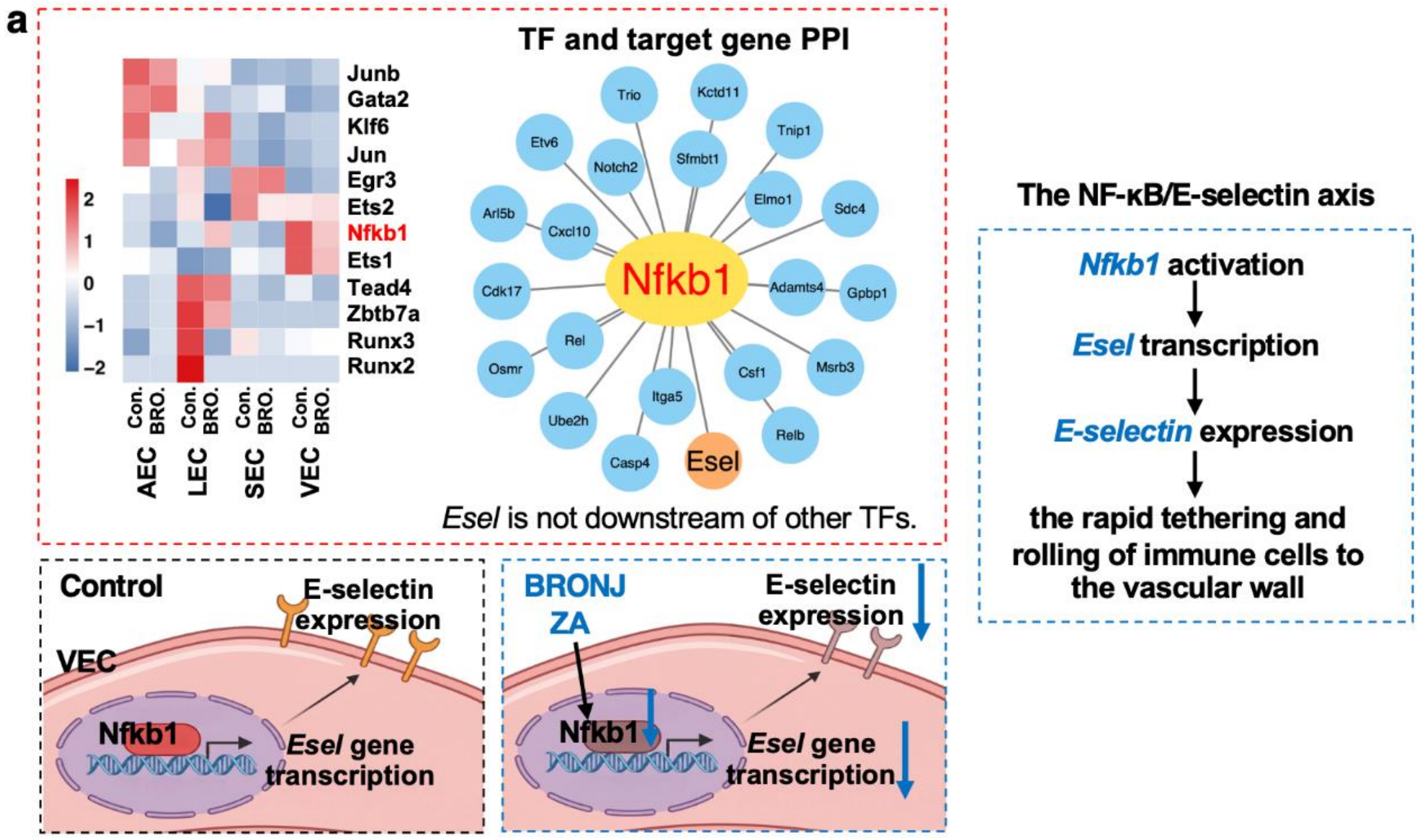

(Supplementary Fig. 9) **a** SCENIC analysis of EC subtypes revealing differential transcription factor (TF) activities and the protein-protein interaction (PPI) network of their target genes. The schematic diagram illustrates the predicted regulatory mechanism underlying *Esel* downregulation.

Response to Reviewer 3:

This study aims to provide a valuable multimodal atlas of bisphosphonate-related osteonecrosis of the jaw (BRONJ) integrating whole-organ imaging, single-cell transcriptomics, and proteomics in mouse and human mandibles. Using bioinformatic analysis, the authors identify endothelial signaling alterations as central features of disease pathogenesis, particularly elevated Col2a1 expression in endothelial cells, which is predicted to act as a paracrine hub through the COL2A1-CD44/SDC4 axes. Although the integrative approach is technically robust and the cross-species validation compelling, the data is still descriptive at the current stage and is only sufficient to support correlation, not a causal relationship, between the vascular changes and bone defects. In addition, the authors do not appear to have carefully considered the temporal factors relevant to the progression of BRONJ. Both the vascular alterations and single-

cell analyses should be interpreted as contributing mechanistic factors in the context of early disease development, before the phenotype is fully established (e.g. day 56 in Fig. 1f). Mechanistically, because CellChat predictions are based on ligand and receptor expression rather than actual signaling activity, the authors should also examine whether the COL2A1-CD44/SDC4 signaling network is indeed activated in vivo and verify its functional role in BRONJ progression. Overall, the study is not mechanistic and preliminary.

Response: We appreciate the reviewer's thoughtful and constructive comments. To address the concerns regarding the disease timeline and mechanistic causation, we have performed a series of supplemental experiments: (1) a detailed time-course assessment (Days 1–28 post-extraction) of vascular and stromal dynamics; (2) *in vivo* functional validations of the endothelial *Col2a1* axis via localized siRNA and conditional knockout (*Col2a1*$^{i\Delta EC}$) mice; and (3) *in silico* validation using scTenifoldKnk virtual knockout. These genetic, pharmacological, and computational analyses strengthen the evidence supporting our proposed mechanism. Our specific point-by-point responses are as follows:

Q1. The authors have established a whole-mount imaging protocol optimized for exogenous antibody labeling of the intact tooth-jawbone structure (lines 82–86). This method will be very valuable for the field. However, the red signals appear to be non-specific particularly within the dental pulp, most notably in in Figure 1 B (α-SMA) and 1C (CD31).

Response: We thank the reviewer for pointing out this issue. The non-specific red signals in the dental pulp were optical artifacts caused by density differences between dental tissues and the alveolar bone during tissue clearing. To address this, we have re-processed the imaging data and optimized parameters to minimize background intensity.

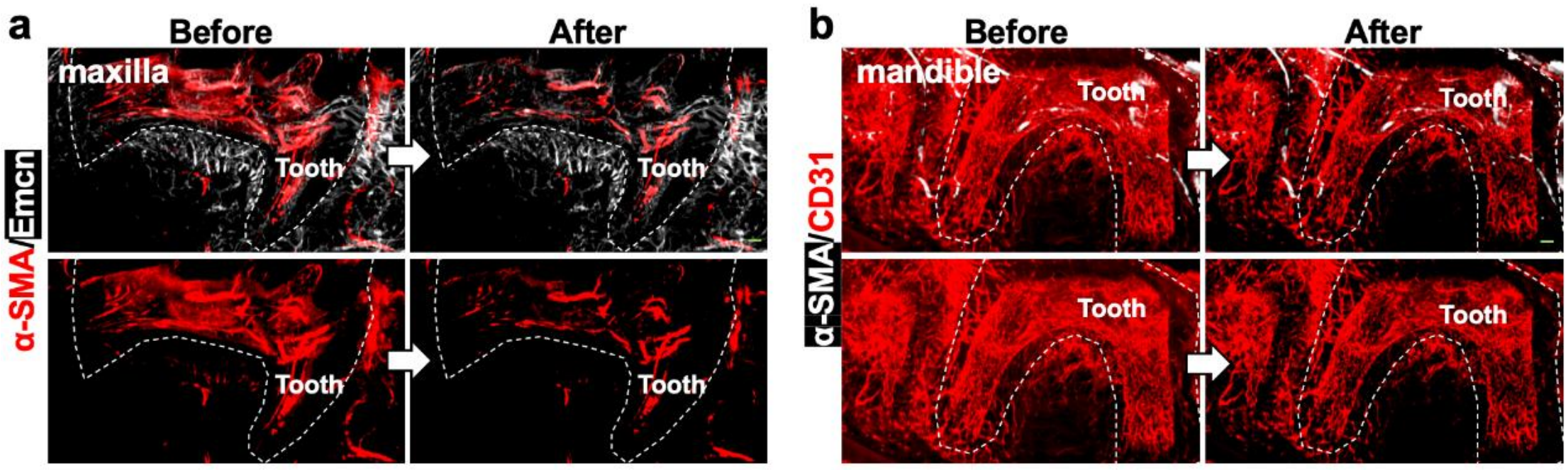

Optimization of red background signals. a Maxillary alveolar bone region before and after adjusting the red background signal for α-SMA in Figure 1b. **b** Mandibular alveolar bone region before and after adjusting the red background signal for CD31 in Figure 1c.

Based on your valuable feedback, we have updated Figure 1b and c in the revised manuscript with the background-adjusted images.

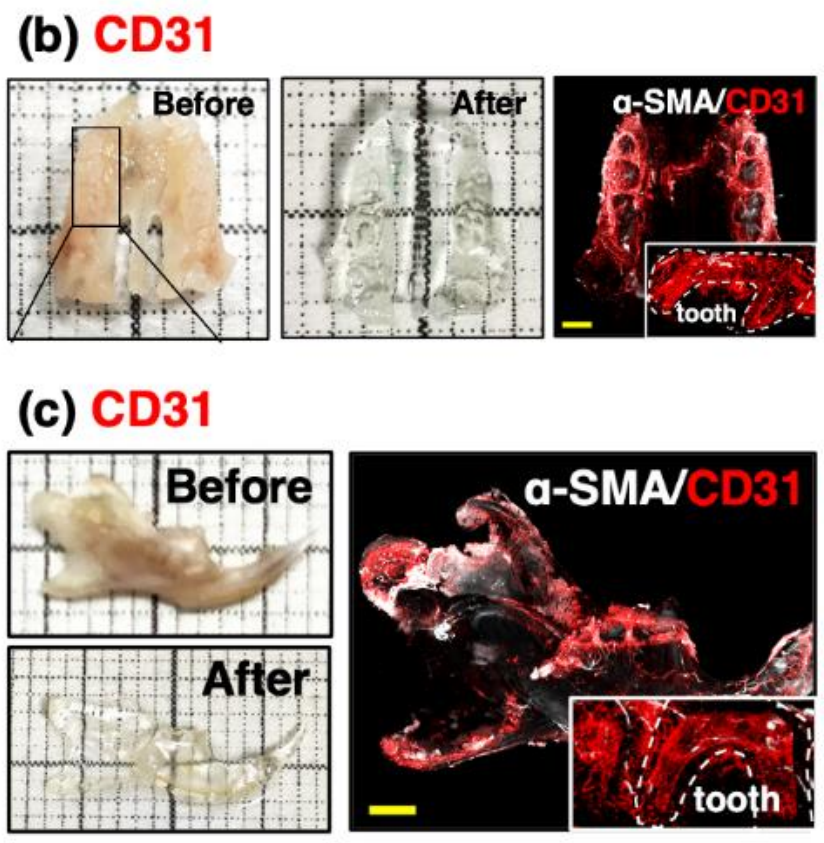

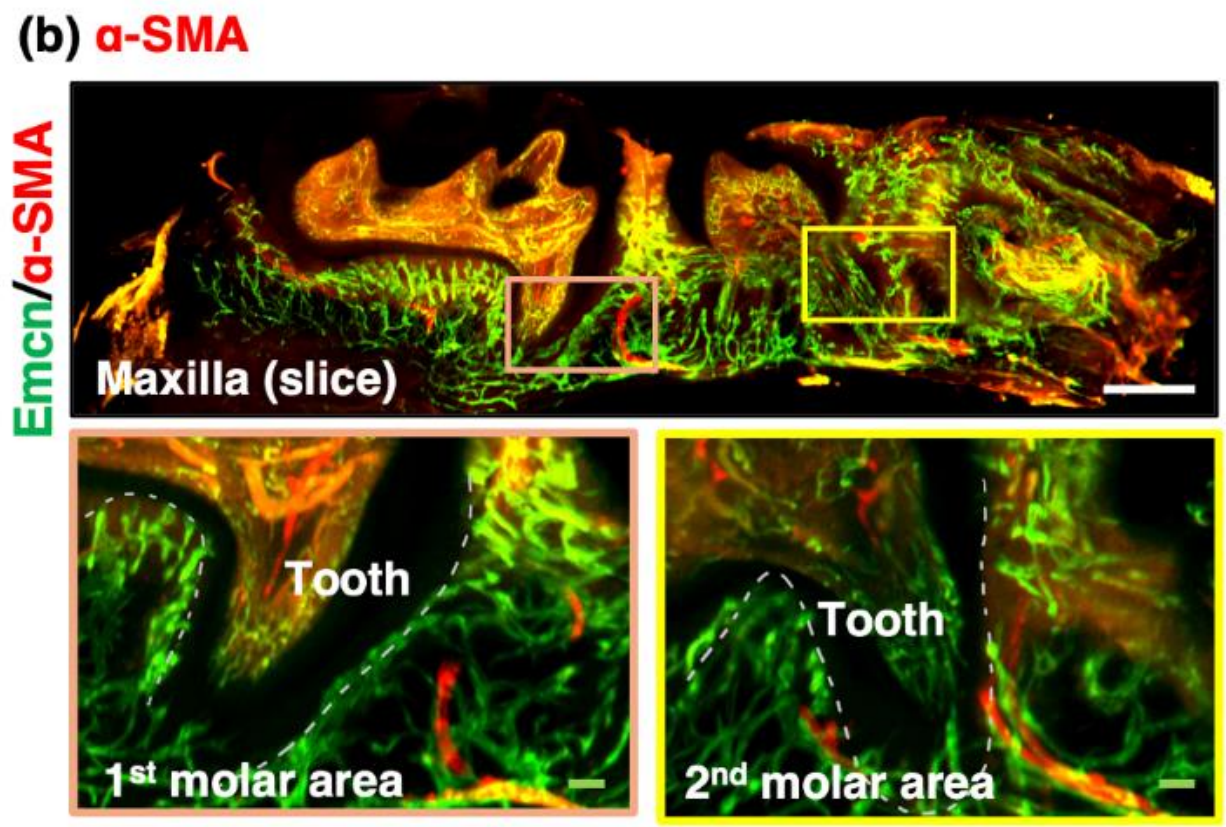

Q2. Many figures/data are missing information on the timing of sample collection. Please notate the time points consistently across all figures in the manuscript.

Response: We thank the reviewer for this comment. We have systematically updated all figures, legends, and corresponding text sections to consistently display the exact time points of sample collection.

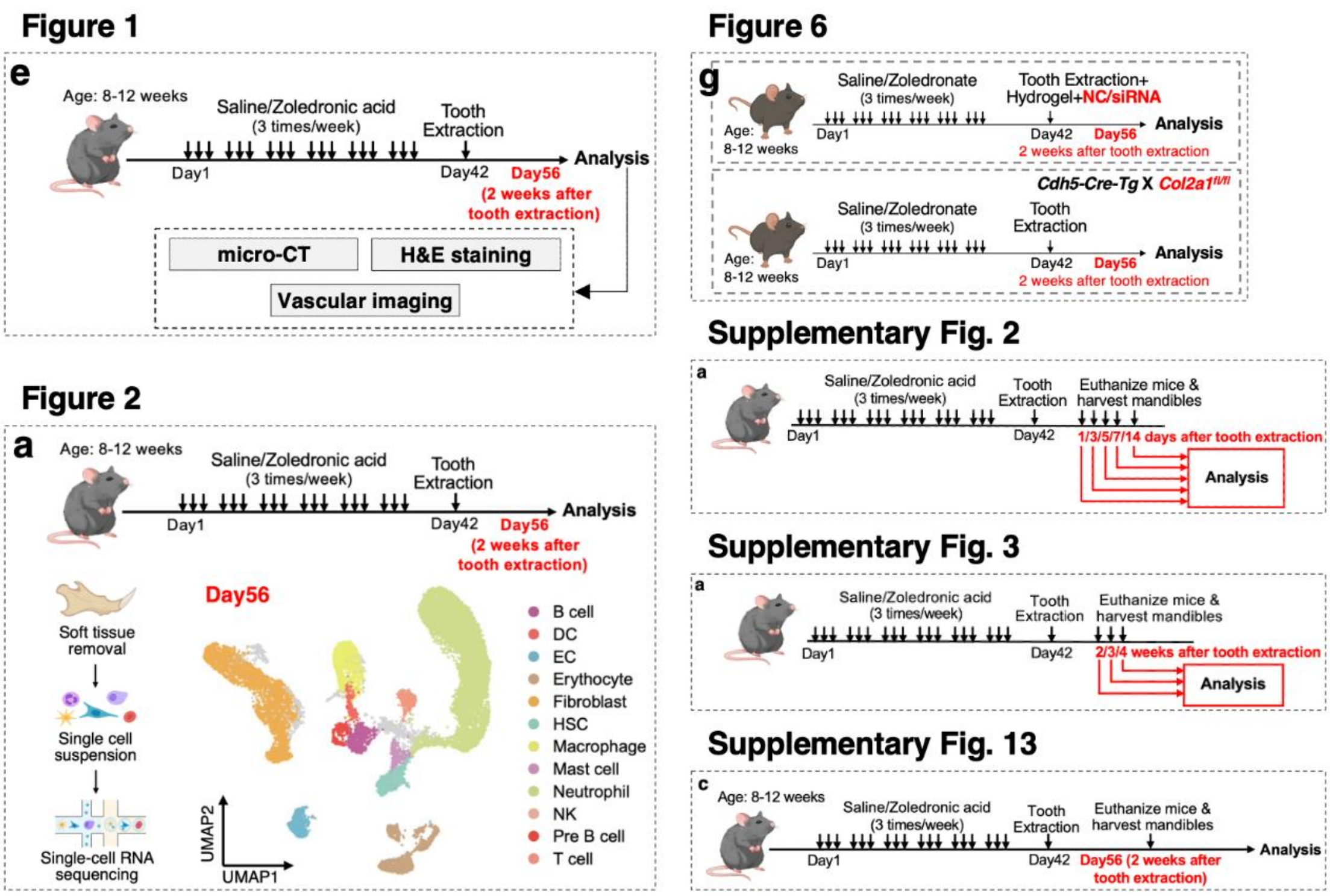

Q3. Lines 96–97: The authors reported 3 vascular subtypes with distinct spatial distributions within the mandibular alveolar bone, based on the co-expression patterns of Emcn and Podocalyxin. Could the authors explain the rationale for selecting these two specific markers to evaluate vascular heterogeneity? In addition, please clarify the spatial distribution differences between the 3 identified vascular subtypes ($Emcn^{lo}Podocalyxin^{hi}$, $Emcn^{hi}Podocalyxin^{hi}$, and $Emcn^{hi}Podocalyxin^{lo}$) in the

mandibular alveolar bone.

Response: Thank you for your constructive comments. Upon systematically re-evaluating our data, both *Emcn* and *Podxl* consistently ranked among the top five most highly specific markers for the local vascular network. Moving forward, we plan to further characterize other highly expressed endothelial markers in future studies.

Feature Name	Endothelial Average	Endothelial Log2 FC	Endothelial P-Value	Fibroblast Average	Fibroblast Log2 FC	Fibroblast P-Value	Erythocyte Average	Erythocyte Log2 FC	Erythocyte P-Value	Immune cell Average	Immune cell Log2 FC	Immune cell P-Value
Cdh5	5.931691	8.7396916	4.26E-194	0.0232662	-3.2525255	3.30E-31	0.0274016	-2.5979126	0.0006815	0.0082667	-5.6651483	3.77E-122
Ptprb	4.4731184	9.0565646	4.26E-194	0.018164	-3.1735491	3.88E-28	0.0152939	-2.9854342	0.0002418	0.0031306	-6.6442216	6.08E-135
**Emcn**	5.0648265	8.4199296	2.98E-169	0.0253071	-2.9170691	4.23E-25	0.0286761	-2.332901	0.0030484	0.0086091	-5.3991446	4.73E-108
Flt1	11.242454	8.0256154	1.64E-162	0.0492876	-3.1660408	4.60E-30	0.0356859	-3.2256856	4.67E-05	0.0406488	-4.300158	1.28E-82
**Podxl**	3.0912487	8.2534279	1.12E-156	0.0162251	-2.8575811	1.77E-23	0.0210292	-2.0718605	0.0095579	0.0063101	-5.1392383	6.13E-96
Kdr	2.5738084	8.2812549	2.43E-149	0.0147965	-2.713994	1.29E-20	0.0114705	-2.6497892	0.0016138	0.0048426	-5.2555602	8.33E-93
Plvap	3.0875961	8.1774983	1.37E-147	0.0233682	-2.3038046	2.63E-16	0.0121077	-2.8516092	0.0007071	0.0044024	-5.6815313	1.18E-104
Sox7	1.5766707	8.6023547	8.53E-147	0.0080615	-2.8511596	3.65E-20	0.0082842	-2.3489043	0.0073565	0.001761	-5.9752028	2.38E-94
Cyyr1	5.9852613	7.8790831	1.12E-145	0.0534714	-2.0878196	6.56E-15	0.0127449	-3.7791978	8.81E-06	0.0128648	-5.1164197	4.33E-101
Cldn5	2.6955591	8.8949699	5.22E-137	0.0127556	-2.9502582	1.21E-18	0.0127449	-2.5145438	0.0111773	0.0016631	-6.8211525	8.97E-100

Identification of the top 10 most highly specific marker genes defining the EC clusters.

Regarding the differences in spatial distribution, we investigated these patterns using both single-cell transcriptomics and immunofluorescence imaging. AECs, representing arteries and arterioles, correspond to the Podocalyxin^{hi} Emcn^{lo} subtype, while VECs comprise both the Podocalyxin^{hi} Emcn^{hi} and Podocalyxin^{lo} Emcn^{hi} subtypes. Spatially, the alveolar bone displays a distinct apical-to-coronal gradient characterized by decreasing Emcn and increasing Podocalyxin. Consequently, Podocalyxin^{lo} Emcn^{hi} VECs localize at the alveolar-mandibular junction, Podocalyxin^{hi} Emcn^{hi} VECs dominate the apical region, and the Podocalyxin^{hi} Emcn^{lo} arterial network restricts primarily to coronal, gingival-facing aspects. **(Page 6)**

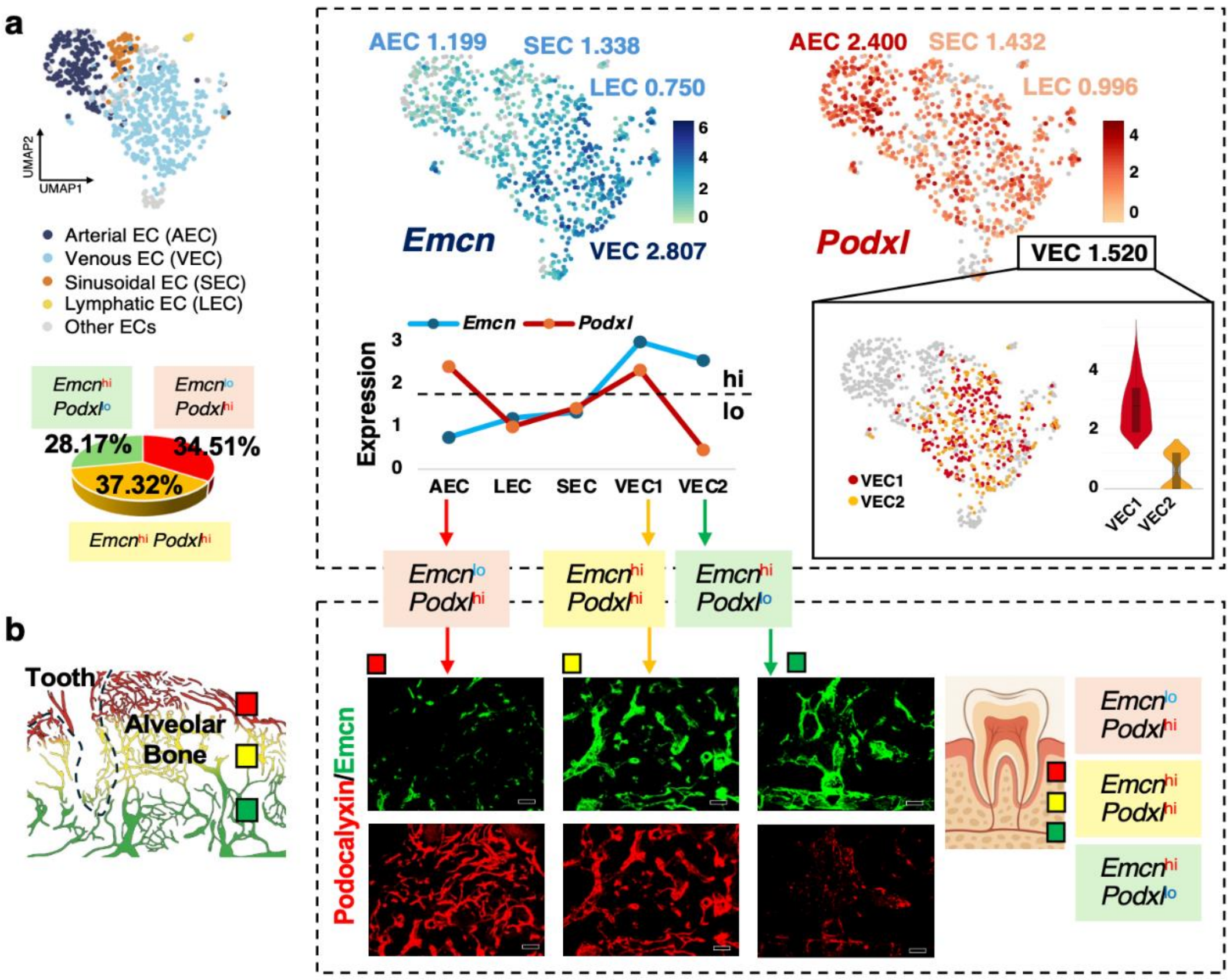

(Supplementary Fig. 8) a scRNA-seq analysis detailing the expression profiles, relative abundance, and cluster distribution of Emcn and Podxl across endothelial cell subpopulations. **b** Representative immunofluorescence images illustrating the spatial localization of $Emcn^{hi}$ and $Podocalyxin^{hi}$ vascular networks within the mandible.

Q4. In Figures 1g, 1h, and S1f, the whole-organ imaging nicely demonstrates vascular and lymphatic attenuation in BRONJ. However, to support the conclusion that endothelial cells play a central role in the pathogenic mechanism of BRONJ, the authors should clarify whether these vascular changes represent a cause rather than a consequence of bone necrosis. While it is understandable that time-course analysis may not be feasible for human samples across disease stages, the authors should take advantage of this mouse model to perform a time-course analysis following zoledronate treatment/tooth extraction to determine when vascular defects first arise and whether they precede necrotic changes.

Response: We thank the reviewer for this insightful comment. To establish the precise temporal hierarchy between vascular attenuation and bone necrosis, we performed a high-resolution time-course analysis (Days 1, 3, 5, 7, and 14 post-extraction): our time-course analysis revealed that a profound reduction in blood vessels was evident by Day 1 and persisted throughout the 14-day observation window (**Supplementary Fig. 2b**), whereas definitive histological bone necrosis (empty lacunae) was not evident until Day 14 (**Supplementary Fig. 2a**). Because microvascular collapse precedes skeletal degeneration, these findings support the notion that vascular inhibition is an upstream pathogenic event rather than a secondary consequence of bone necrosis. (**Page 4-5**)

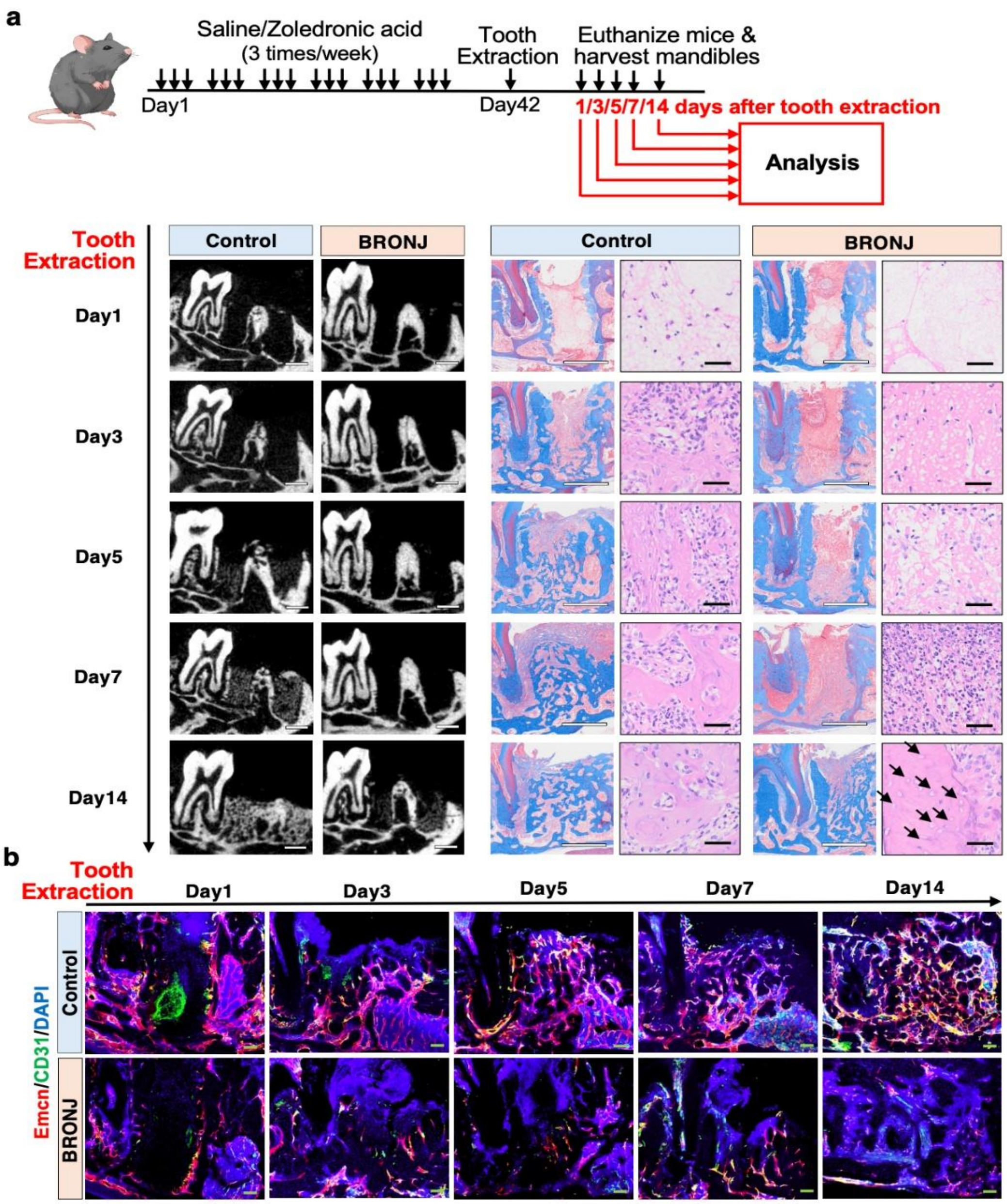

(Supplementary Fig. 2: Time-course evaluation at 1, 3, 5, 7, and 14 days post-tooth extraction.) **a** Representative micro-CT images of new bone formation, alongside histological assessments using Masson's trichrome and hematoxylin and eosin (H&E) staining in the control and BRONJ groups at the indicated time points. Arrows indicate empty osteocyte lacunae. **b** Representative immunofluorescence images of mandibular vasculature in the control and BRONJ groups at 1, 3, 5, 7, and 14 days post-extraction. Sections were immunostained for Emcn and CD31, with DAPI used for nuclear counterstaining. **Scale bars:** white, 500 µm; green, 100 µm; black, 50 µm.

Q5. A key piece of missing information concerns the timing of the single-cell transcriptomic profiling of healthy and BRONJ mouse mandibles. The authors must specify how long after zoledronate treatment/tooth extraction the samples were collected. Moreover, to elucidate the mechanisms contributing to bone defects, the single-cell experiments should be performed at an earlier stage when molecular changes are still initiating these pathological changes, rather than at day 56 (4 weeks post-

zoledronate treatment) as shown in Figure 1f, when necrotic bone defects are already well developed. If performed at earlier stages, would CellChat analysis still reveal similar significantly enhanced endothelial cell-to-fibroblast/macrophage signaling in BRONJ?

Response: We thank the reviewer for this important comment. In this study, scRNA-seq was performed 2 weeks post-extraction (day 56), following ZA pre-treatment. To better define the pathological stage represented by day 56, we performed additional histological evaluations at 3- and 4-weeks post-extraction. These analyses suggest that day 56 captures an ongoing pathological transition, with unhealed fibrous tissue and emerging empty lacunae at 2 weeks post-extraction, followed by more extensive necrosis and bone sequestrum at weeks 3 and 4. We agree that earlier time points may provide additional insight into BRONJ initiation and will incorporate such analyses in future studies. **(Page 5)**

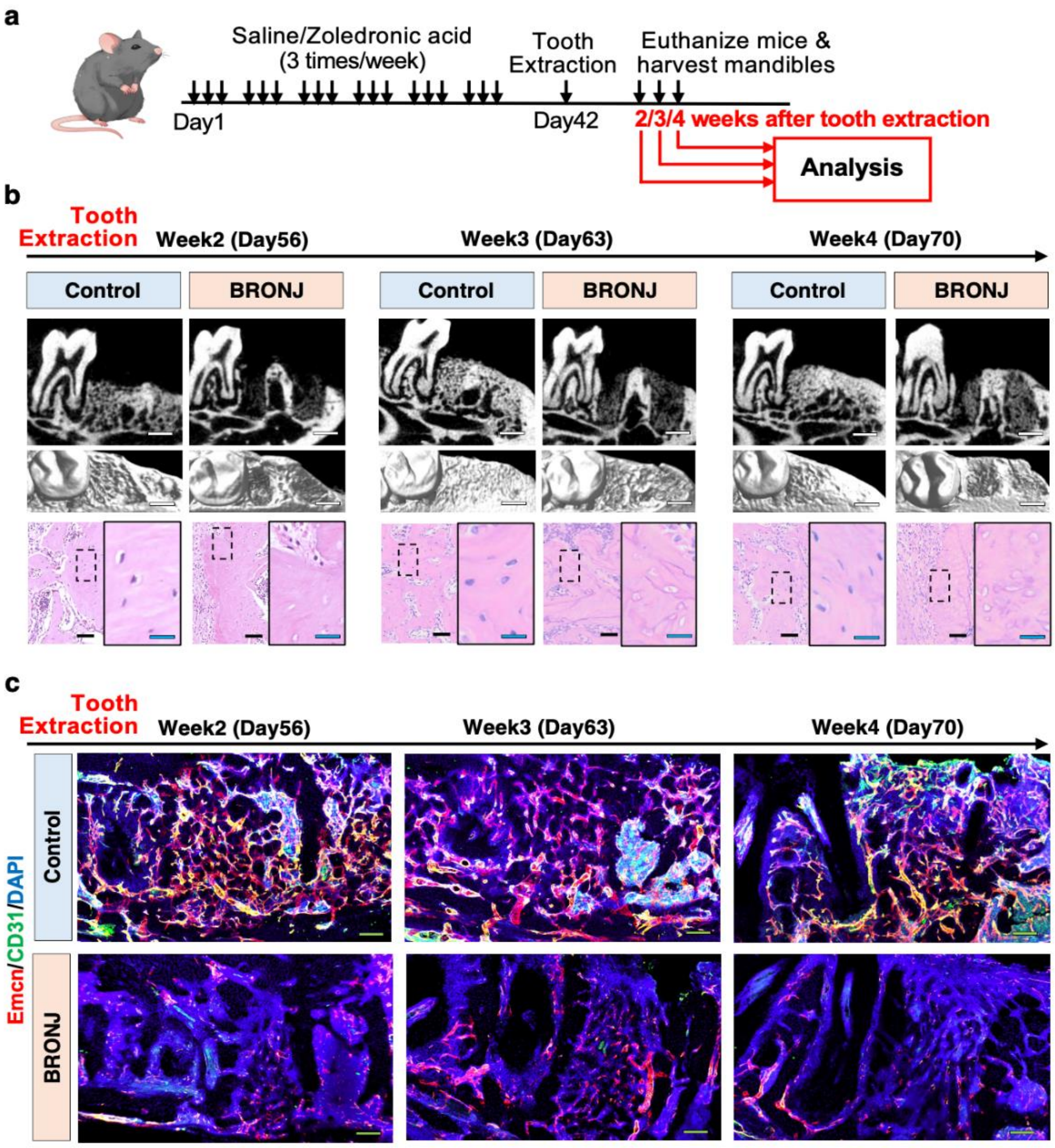

(Supplementary Fig. 3: Time-course evaluation at 2, 3, and 4 weeks post-tooth extraction.) a Schematic diagram illustrating the timeline for experimental model establishment. **b** Representative micro-CT images of new bone formation, alongside histological assessments using H&E staining

in the control and BRONJ groups at the indicated time points. **c** Representative immunofluorescence images of mandibular vasculature in the control and BRONJ groups at 2, 3, and 4 weeks post-extraction. Sections were immunostained for Emcn and CD31, with DAPI used for nuclear counterstaining. **Scale bars:** white, 500 µm; green, 100 µm; black, 50 µm; blue, 20 µm.

Q6. Using bioinformatic analysis (CellChat), the authors report elevated Col2a1 expression in endothelial cells and propose that it acts through the COL2A1-CD44/SDC4 axis in fibroblasts and macrophages, likely promoting extracellular matrix remodeling and inflammation. Because CellChat predictions are based on ligand and receptor expression rather than actual signaling activity, the authors should validate these findings by examining in vivo gene expression changes of the COL2A1-CD44/SDC4 axis ligands or receptors and, more importantly, the corresponding signaling readouts in mouse and human BRONJ tissue sections. The authors should then test whether restoring the COL2A1-CD44/SDC4 axis can rescue the bone defects by modulating matrix remodeling and inflammation to substantiate their proposed functional role.

Response: We thank the reviewer for this insightful comment. As suggested, targeted RT-qPCR confirmed a significant, concurrent upregulation of *Col2a1* and its functional receptors (*Cd44* and *Sdc4*) in the BRONJ group compared to controls. Correspondingly, Collagen II deposition was found in the BRONJ microenvironment. (**Page 10**)

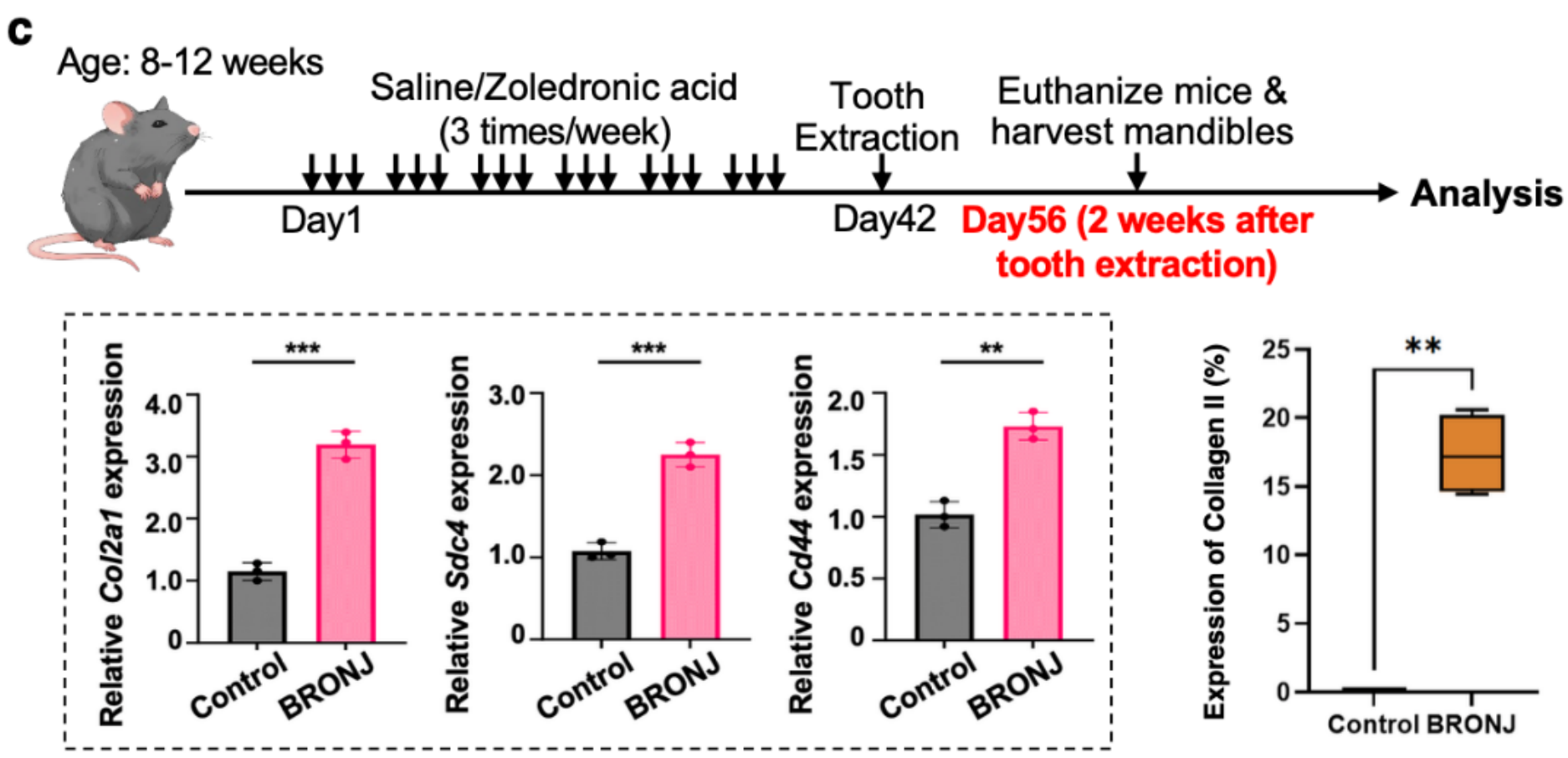

(Supplementary Fig. 13) b RT-qPCR evaluation of *Col2a1* and its associated receptors Sdc4 and Cd44 in BRONJ, alongside statistical analysis of Collagen II immunofluorescence staining (*n=4*). **P < 0.01; ***P < 0.001.

Then we engineered a localized delivery system by implanting *Col2a1*-targeting siRNA-loaded hydrogels directly into the extraction sockets of ZA-treated mice. This local intervention successfully rescued the BRONJ phenotype, significantly promoting soft tissue closure and restoring osteogenesis within the healing socket. (**Page 11**)

b

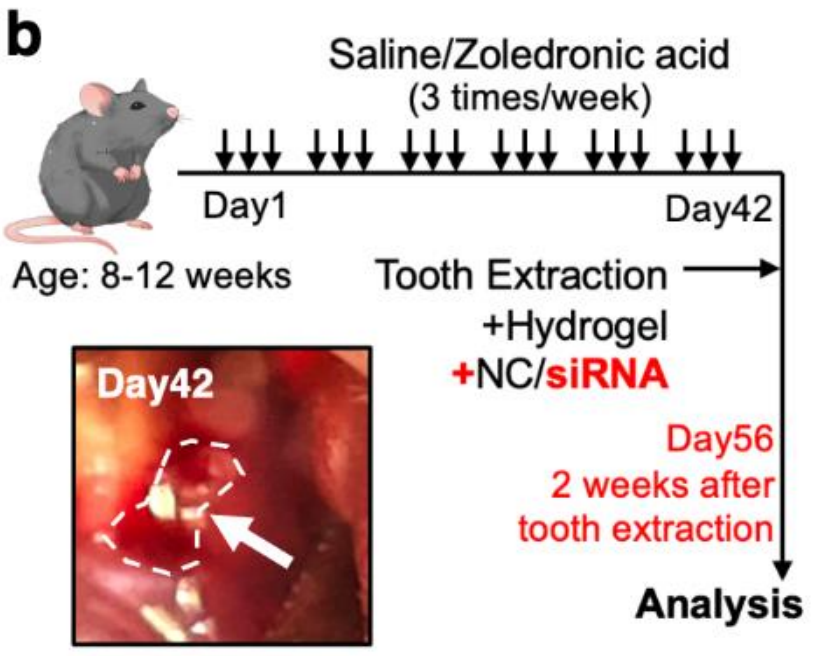

c

Name	Sequence (5'→3')	Modification
mCol2a1 siRNA sense	/i2OMeC/*/i2OMeC/*/i2OMeU/G G/i2OMeC/A/i2OMeU//i2OMeU/ G/i2OMeC//i2OMeU/GG/i2OMeC //i2OMeU//i2OMeU//i2OMeU/AA A*/i2OMeU/*/i2OMeU/	3`Cholesteryl 9mU4*5mC
mCol2a1 siRNA antisense	/i2OMeU/*/i2OMeU/*/i2OMeU/AA AG/i2OMeC//i2OMeC/AG/i2OMe C/AA/i2OMeU/G/i2OMeC//i2OMe C/AGG*/i2OMeU/*/i2OMeU/	6mU4*5mC

d

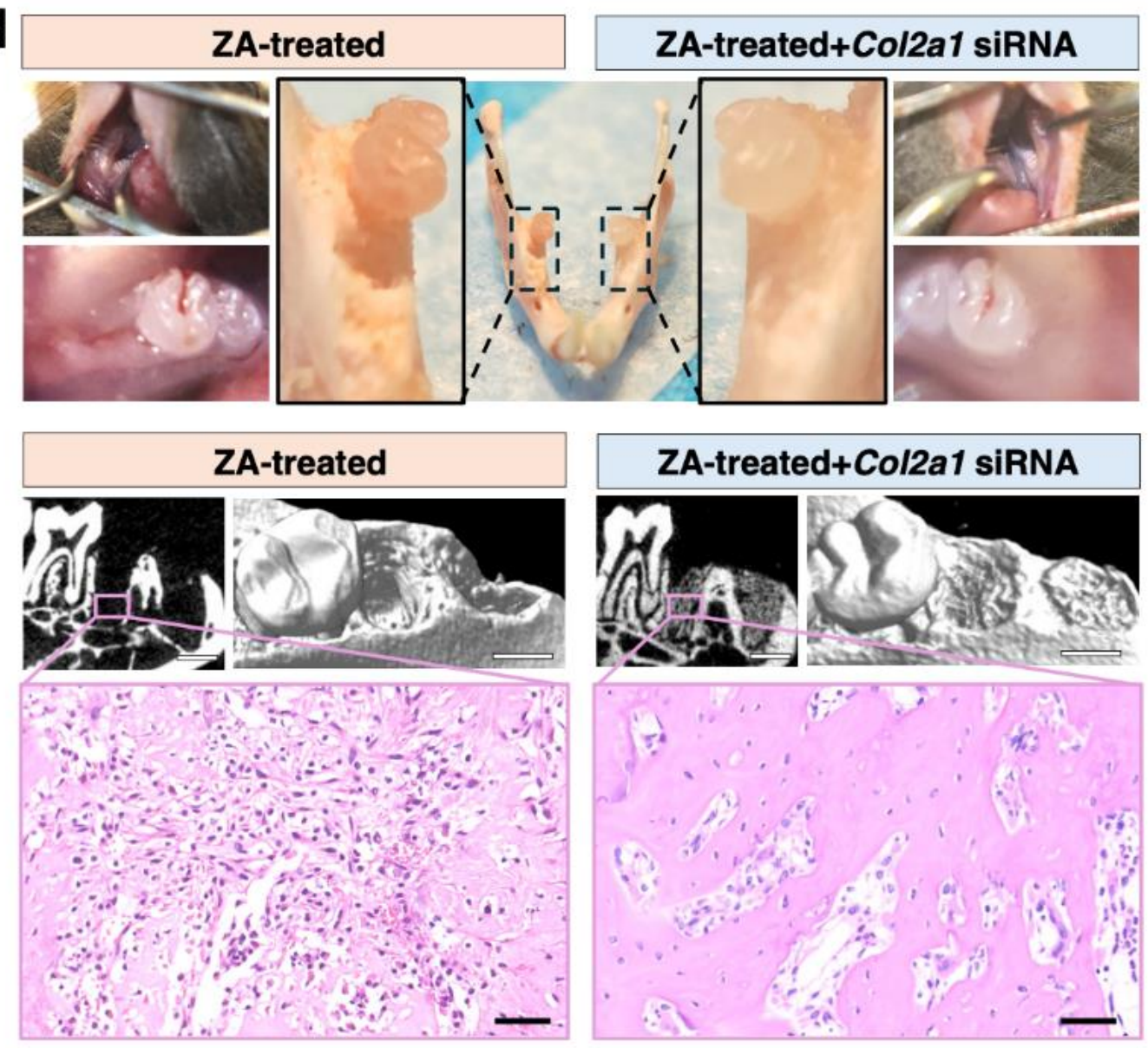

(Supplementary Fig. 15) b Schematic illustration of the local delivery of Col2a1 siRNA-loaded hydrogels into the BRONJ extraction socket. **c** Sequences and modifications of the *Col2a1* siRNA sense and antisense strands. **d** Representative macroscopic images, micro-CT reconstructions, and H&E histological evaluations comparing the treated extraction sockets with the contralateral controls**. Scale bars:** white, 500 µm; black, 50 µm.

Further, to selectively target this axis in the EC, we generated an endothelial-specific *Col2a1* conditional knockout (*Col2a1*$^{i\Delta EC}$) mouse model by crossing *Cdh5-Cre-Tg* (the Jackson Laboratory, no. 017968) and *Col2a1*$^{fl/fl}$ (Shanghai Southern Model Biotechnology Co., Ltd., NM-CKO-240983) mice. Notably, endothelial-specific deletion of *Col2a1* accelerated vascular recovery and enhanced tissue healing. These findings demonstrate that endothelial-derived *Col2a1* upregulation is a primary pathogenic driver of vascular disruption in this model. **(Page 11)**

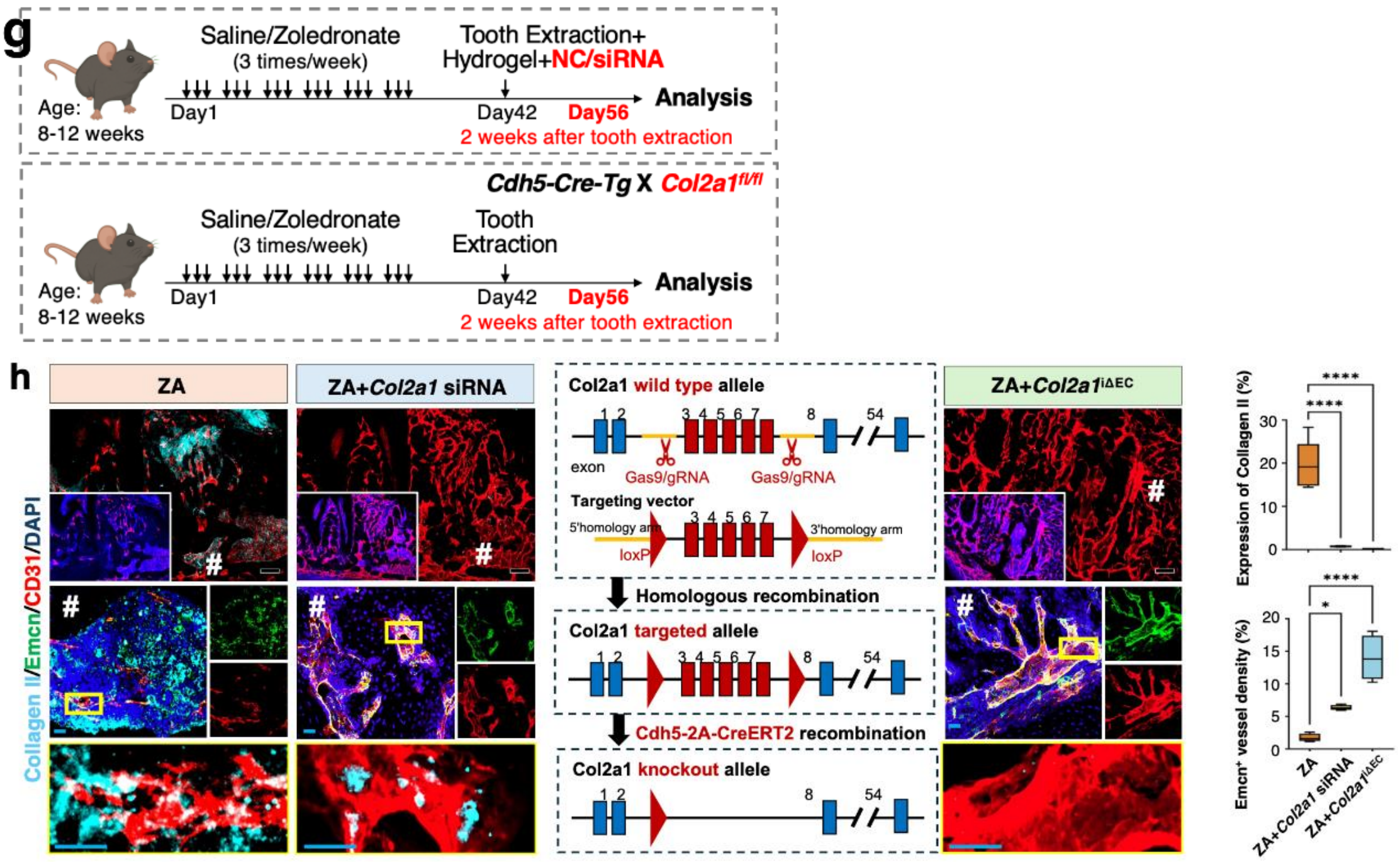

(Fig.6) g Experimental workflow: local delivery of NC or *Col2a1* siRNA via hydrogels immediately after tooth extraction in ZA-treated mice, and *Col2a1*$^{i\Delta EC}$ mice subjected to the same BRONJ induction. **h** Representative immunofluorescence images of the extraction socket, depicting the spatial distribution of blood vessels (*n=4*) and perivascular Collagen II deposition (*n=5*). Sections were immunostained for Emcn, CD31, and Collagen II, with nuclear counterstaining by DAPI. **Scale bars:** black, 50 µm; blue, 20µm. *P < 0.05, ****P< 0.0001.

Finally, to elucidate the underlying mechanisms, we performed a virtual genetic knockout of *Col2a1* within the endothelial cell populations using the scTenifoldKnk algorithm. This single-cell perturbation analysis demonstrated that the loss of endothelial *Col2a1* significantly alters downstream gene regulatory networks directly governing Angiogenesis, Inflammation, and Osteogenesis. **(Page 11)**

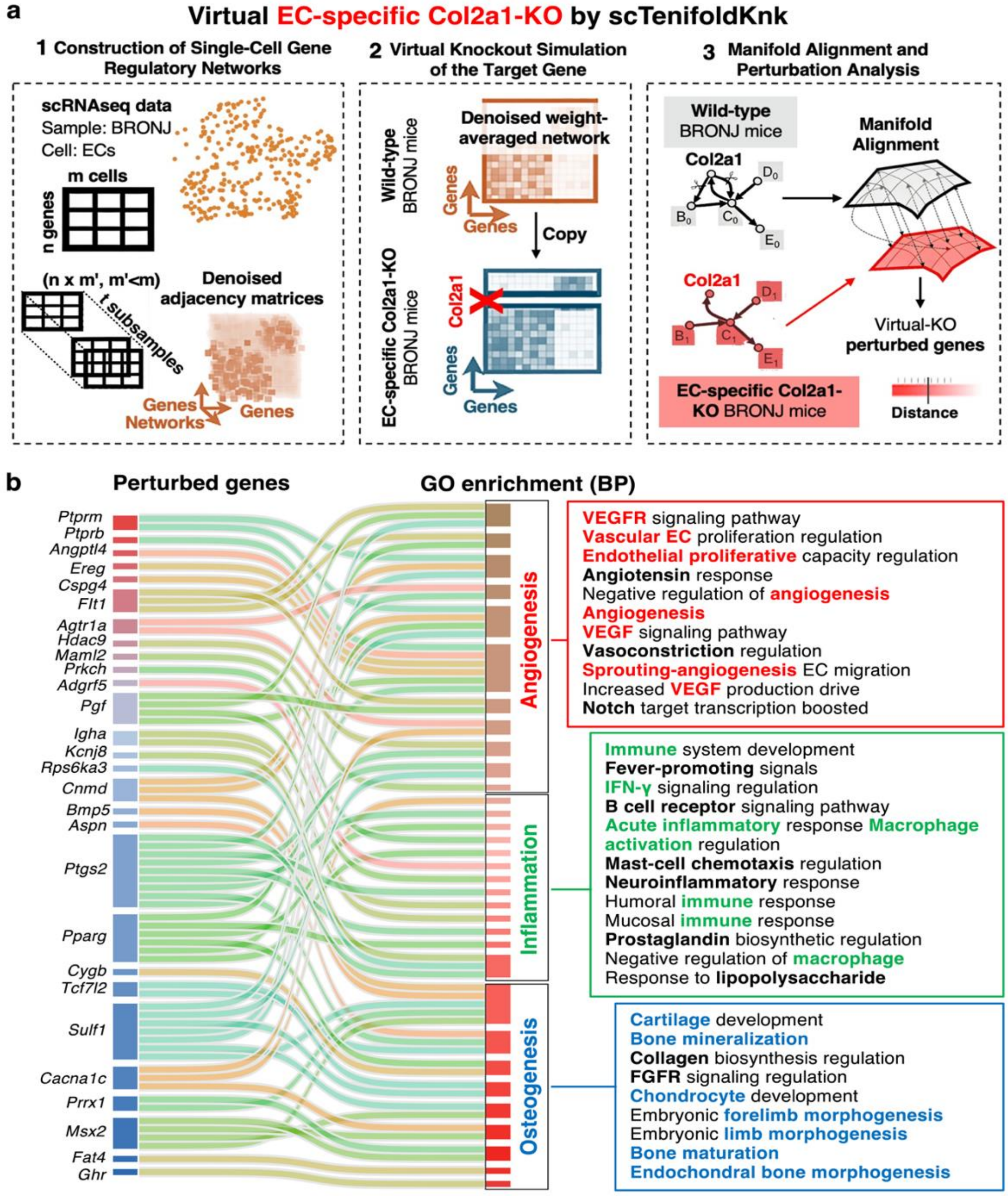

Supplementary Fig. 16: Virtual endothelial *Col2a1* knockout via scTenifoldKnk.
a Schematic illustration of the computational workflow for the virtual gene knockout analysis. **b** GO enrichment analysis of the significantly perturbed gene network following *Col2a1* deletion,

highlighting key biological processes associated with angiogenesis, inflammation, and osteogenesis.

Point-by-point response

We sincerely appreciate the editor and the reviewers' time and insightful comments. We have carefully revised the manuscript to address all the concerns raised and believe that the revised version has been significantly improved. All modifications are highlighted in yellow, and our point-by-point responses to the reviewers' comments are provided below.

Response to Reviewer 3:

In this revision, the authors have performed additional experiments, improved data quality, and added experimental procedural details, all of which significantly strengthen the study. However, some concerns remain and will need to be clarified to make the conclusions and findings more solid.

***Response*:** We sincerely appreciate your positive evaluation and constructive feedback. In this revision, we have further strengthened our study by providing better anatomical overviews and quantitative statistics of vascular remodeling, alongside comprehensive spatiotemporal and in vivo mechanistic evidence confirming the causal role of the endothelial *Col2a1-Sdc4/Cd44* axis in BRONJ pathogenesis.

Q3: Defining the in vivo locations of the three vascular subtypes by Emcn/Podocalyxin co-expression is a valuable finding. However, Supplementary Fig. 8b shows only high-magnification images of the individual expression patterns, making the anatomical distribution hard to appreciate. A lower-magnification image of the co-expression patterns, with higher-magnification images as insets, comparable to the schematic in Supplementary Fig. 8b, would be necessary.

***Response*:** We sincerely appreciate the reviewer's constructive suggestion. To make the anatomical distribution and co-expression patterns readily apparent, we have updated **Supplementary Fig. 8b** to include low-magnification overview images alongside high-magnification insets and corresponding schematics. **(Page 6)**

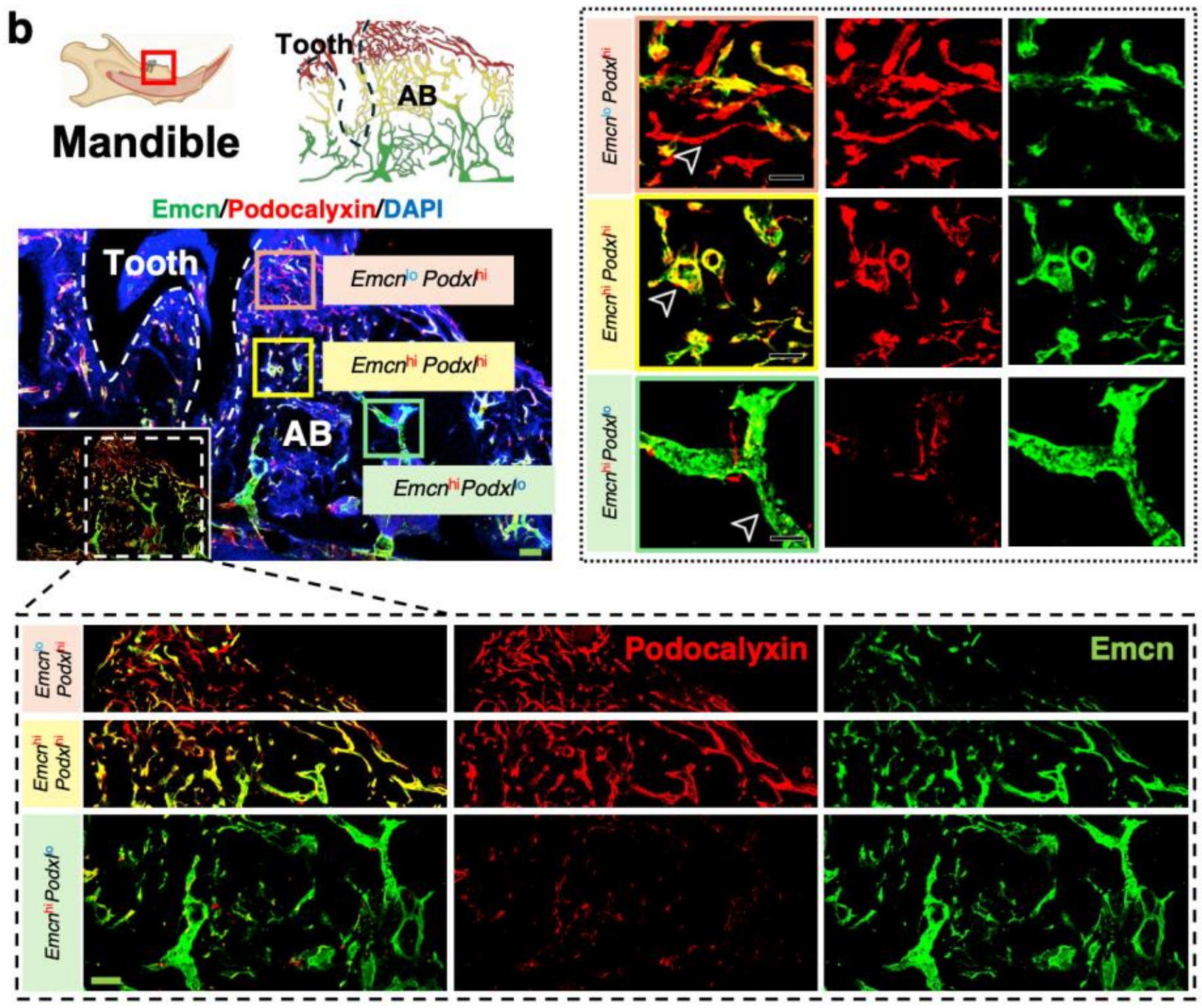

(Supplementary Fig. 8) **b** Representative immunofluorescence images illustrating the spatial localization and abundance of Emcn^{hi} and Podxl^{hi} vascular networks within the mandible. The right and bottom panels show high- and low-magnification views, respectively. **Scale bars:** green, 100 µm; black, 50 µm.

<u>Q4: Quantitative statistical analysis, in addition to the images, is needed to evaluate the change in vascularity in Supplementary Fig. 2b.</u>

Response**:** We agree with the reviewer's suggestion. We have performed a quantitative statistical analysis of vessel density changes over time across the Control and BRONJ groups (*n* = *5*). The quantitative statistical graphs, along with informative schematic diagrams illustrating the dynamic vascular changes over the course of alveolar socket healing, have been added as **Supplementary Fig. 2c**. **(Page 5)**

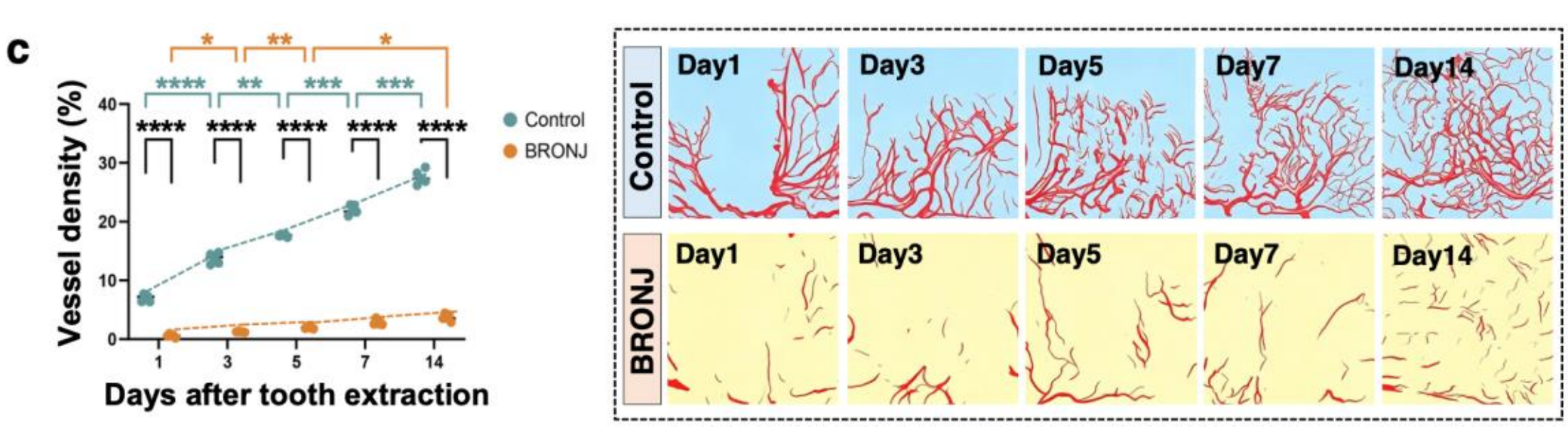

(Supplementary Fig. 2) **c** Statistical analysis of vessel density changes over time in the Control and BRONJ groups (*n*=*5*), and schematic diagrams of vascular changes. **Scale bars:** white, 500 µm; green, 100 µm; black, 50 µm. *P < 0.05; **P < 0.01; ***P < 0.001; ****P < 0.0001.

Q5: It is understandable that the authors may not be able to perform additional scRNA-seq experiments at earlier time points. Since the authors already have time-course sections from day 1 to day 14 post tooth extraction, could the authors still try to evaluate whether the endothelial cell-to-fibroblast/macrophage signaling identified by CellChat is already significantly enhanced before definitive histological bone necrosis becomes evident? This would help establish that endothelial cell-to-fibroblast/macrophage signaling is a contributing factor to the necrosis rather than a downstream event following necrosis.

***Response*:** We thank the reviewer for this important suggestion. To determine whether endothelial crosstalk precedes tissue necrosis, we evaluated the kinetics of *Col2a1–Sdc4/Cd44* signaling from Days 1 to 14 post-extraction using Collagen I and CD68 as markers for fibroblasts and macrophages, respectively (**Supplementary Fig. 14**). Notably, between Days 1 and 7, and well before definitive histological bone necrosis appeared at Day 14, the BRONJ group exhibited significantly higher endothelial Col2a1 expression, stronger Sdc4 expression on perivascular Collagen I^+ fibroblasts, and increased CD44 levels on $CD68^+$ macrophages compared with the control group. These spatiotemporal dynamics confirm that endothelial-to-fibroblast/macrophage signaling is an upstream driver of BRONJ pathogenesis rather than a secondary consequence of tissue necrosis. **(Page 10)**

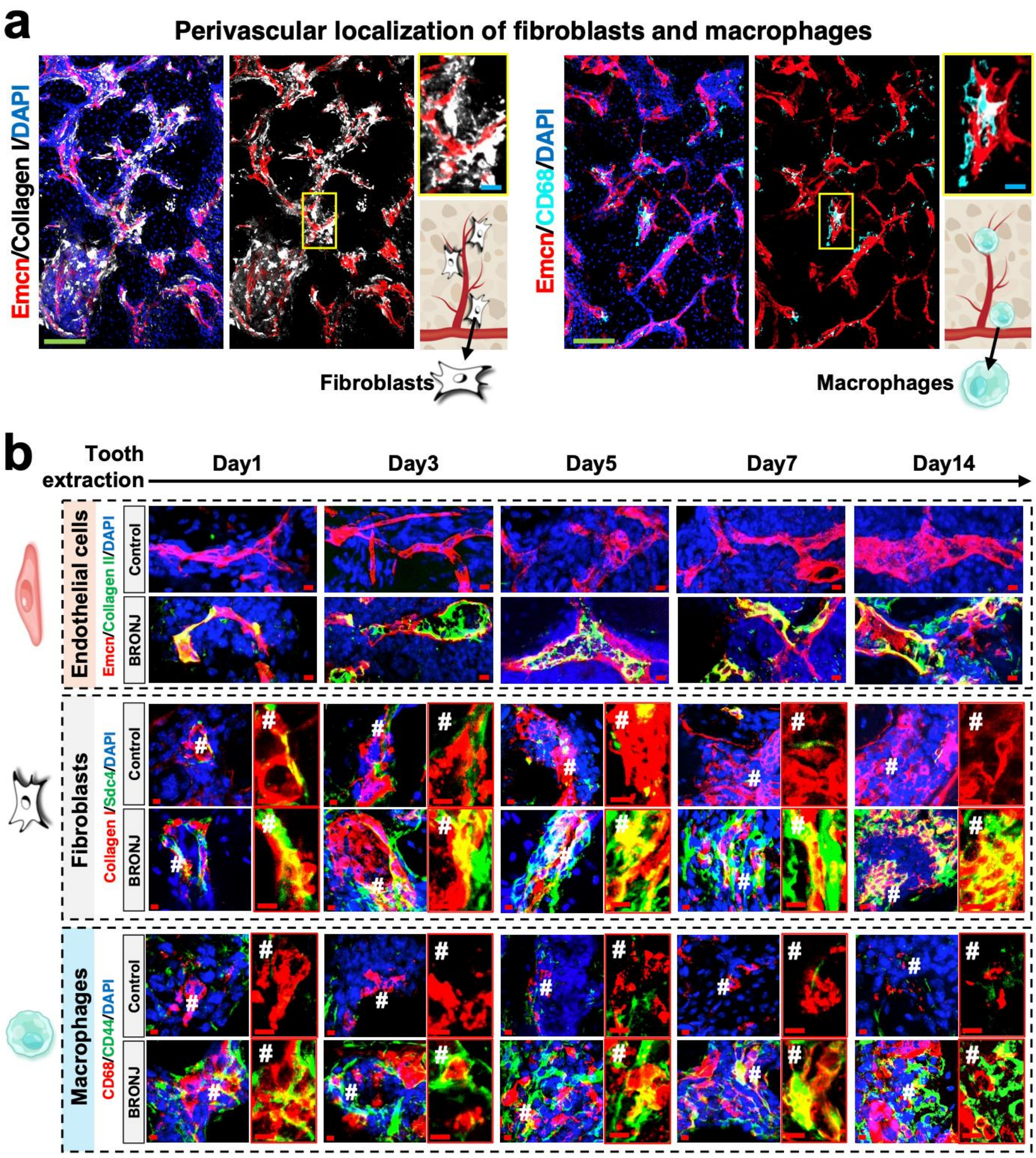

Supplementary Fig. 14: Spatial and temporal characterization of *Col2a1-Sdc4/Cd44* signaling. **a** Perivascular distribution of Collagen I^{+} fibroblasts and CD68^{+} macrophages around Emcn^{+} blood vessels (nuclei counterstained with DAPI). **b** Time-course immunolabeling of endothelial Col2a1 ligand and its receptors Sdc4 (fibroblasts) and Cd44 (macrophages) at days 1, 3, 5, 7, and 14 after tooth extraction. **Scale bars:** green, 100 µm; blue, 20 µm; red, 5 µm.

Q6: Since the authors propose that endothelial-derived Col2a1 acts through the COL2A1–CD44/SDC4 axis in fibroblasts and macrophages, can they confirm that the upregulation of Col2a1 and its receptors (Cd44 and Sdc4) occurs in these fibroblasts and macrophages, preferably using histological sections?

More importantly, the two powerful in vivo tools the authors have used—localized Col2a1-targeting siRNA hydrogel delivery and endothelial-specific Col2a1 conditional knockout mice—should be explored further to dissect the mechanism of the COL2A1–CD44/SDC4 axis in BRONJ. For example, does reduced Col2a1 restore the expression of its receptors Cd44/Sdc4 and, in addition to vascular morphological recovery,

eventually reduce necrosis (e.g., by histology)? Moreover, since the authors bioinformatically predict that Col2a1 deletion perturbs gene networks governing angiogenesis, inflammation, and osteogenesis (Supplementary Fig. 16), could they validate whether this prediction holds in vivo using the existing histology sections for both localized silencing and genetic deletion (Figure 6h)?

***Response*:** We appreciate this valuable suggestion. Firstly, our time-course histological analysis has confirmed the co-upregulation of endothelial *Col2a1* and its receptors (*Sdc4* on fibroblasts and *Cd44* on macrophages) in BRONJ (**Supplementary Fig. 14b**). **(Page 10)**

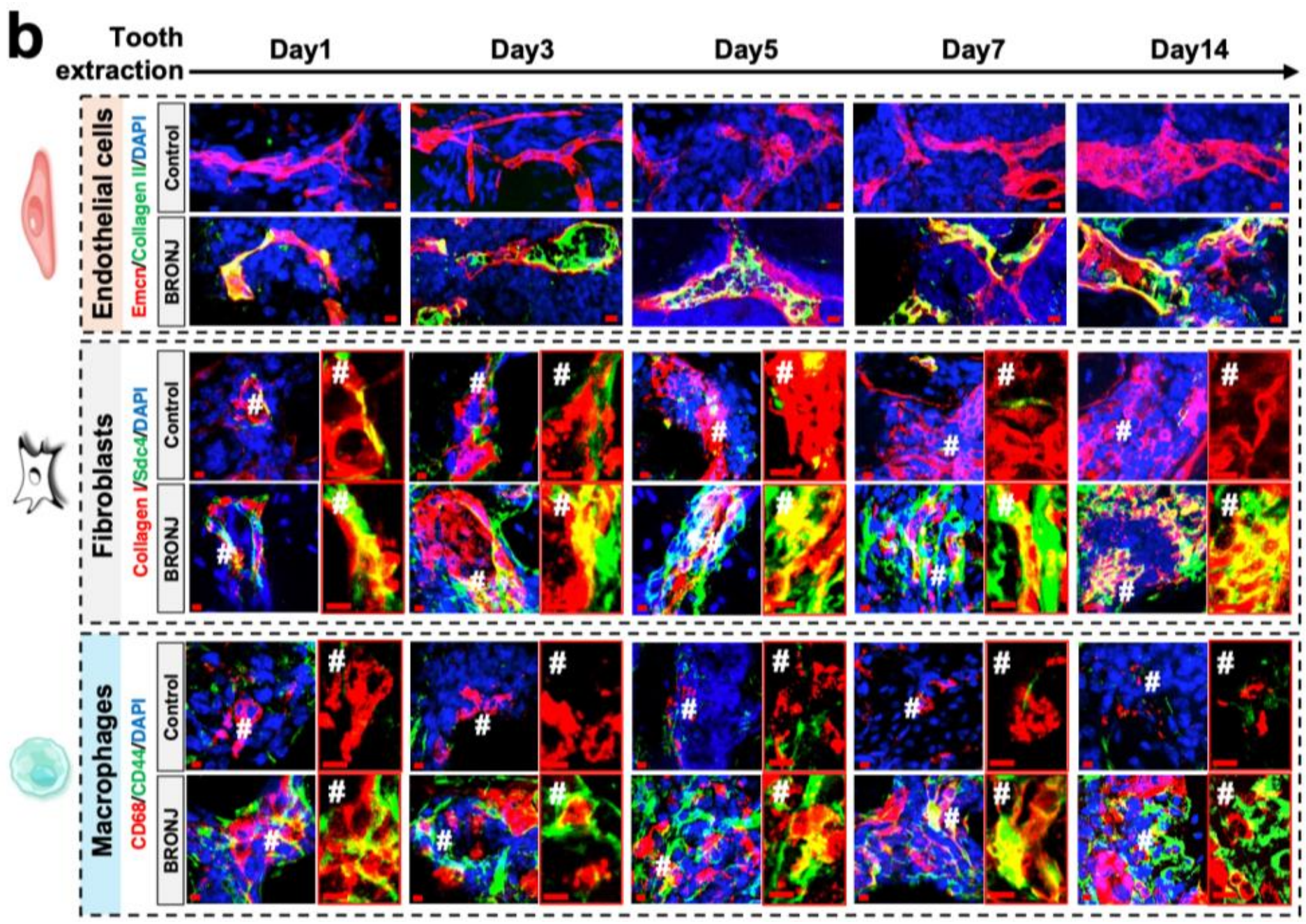

(Supplementary Fig. 14) b Time-course immunolabeling of endothelial *Col2a1* ligand and its receptors Sdc4 (fibroblasts) and Cd44 (macrophages) at days 1, 3, 5, 7, and 14 after tooth extraction.

Additionally, we validated our bioinformatic predictions in vivo using both localized *Col2a1* siRNA delivery and $Col2a1^{i\Delta EC}$ mice (**Supplementary Fig. 18**). Targeted Col2a1 disruption significantly promoted osteogenesis (BMP2$^+$ and RUNX2$^+$ osteoblasts, and OPN expression) and functional angiogenesis while suppressing bone necrosis and inflammation (CD68$^+$ infiltration, IL-1β, and TNF-α) (**Supplementary Fig. 18a, b**). Mechanistically, downregulating endothelial Col2a1 markedly reduced downstream Sdc4 expression on fibroblasts and CD44 on macrophages (**Supplementary Fig. 18c**). Together, these in vivo findings confirm that targeted disruption of the endothelial COL2A1–CD44/SDC4 axis restores osteo-angiogenesis and attenuates BRONJ pathogenesis. **(Page 11)**

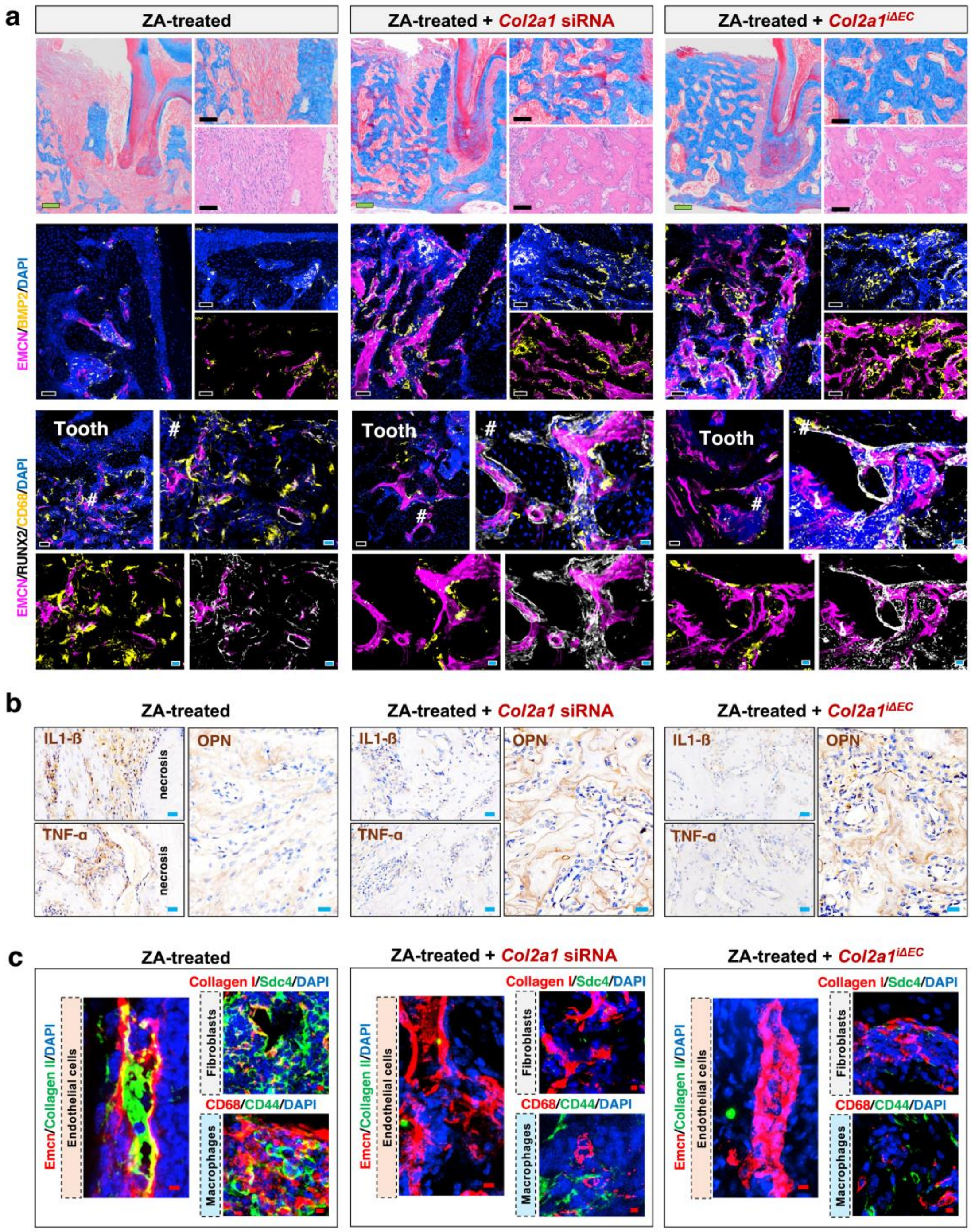

Supplementary Fig. 18: Effects of *Col2a1* disruption on osteogenesis, inflammation, and ligand-receptor interactions in ZA-treated alveolar bone.

a Representative histological and immunofluorescence images of $BMP2^{+}$ and $RUNX2^{+}$ osteoblasts, as well as $CD68^{+}$ inflammatory cells, in the alveolar bone across the ZA-treated, ZA-treated+*Col2a1* siRNA, and ZA-treated+*Col2a1*$^{i\Delta EC}$ groups. **b** Immunohistochemical staining of pro-inflammatory cytokines (IL-1β and TNF-α) and the osteogenic marker osteopontin (OPN) in the alveolar bone. **c** Representative immunofluorescence images illustrating the expression of the endothelial *Col2a1* ligand and its receptors, Sdc4 (on fibroblasts) and CD44 (on macrophages), across the three groups. **Scale bars:** green, 100 µm; black, 50 µm; blue, 20 µm; red, 5 µm.
